# Real-world effectiveness of maternal genetically inactivated acellular pertussis vaccination in protecting infants under three months of age: a case-control study using concurrent community-based and test-negative controls

**DOI:** 10.1007/s15010-026-02868-x

**Published:** 2026-07-27

**Authors:** Farooq Phiriyasart, Virasakdi Chongsuvivatwong, Ponlagrit Kumwichar

**Affiliations:** 1 Su-ngai Kolok Hospital, Narathiwat Provincial Public Health Office, Su- ngai Kolok, Narathiwat Thailand; 2 Department of Epidemiology, Faculty of Medicine, Prince of Songkla University, Hat Yai, Songkhla Thailand

**Keywords:** Maternal immunization, Pertussis, Vaccine effectiveness, Genetically inactivated pertussis vaccine, aPgen, Infant, Real-world evidence, Recombinant pertussis vaccine

## Abstract

**Purpose:**

Infants too young for primary vaccination face the highest risk of severe pertussis and death. While maternal vaccination is the primary protective strategy, the real-world vaccine effectiveness (VE) of acellular pertussis (aP) vaccines in Thailand (including recombinant aP (aP_gen_)) remains unknown. We aimed to evaluate the VE of the aP vaccine in protecting infants under 3 months of age against symptomatic pertussis.

**Methods:**

This case-control study was conducted in the Narathiwat Province, Thailand, during a pertussis outbreak between September 2023 and October 2024. Participants included 33 symptomatic, polymerase chain reaction (PCR)-confirmed cases, 330 asymptomatic, community-matched controls (for primary comparison), and 41 symptomatic, test-negative controls (for sensitivity simulation). Maternal vaccination with aP vaccine during pregnancy was evaluated against no pertussis vaccination. The main outcome measures were symptomatic PCR-confirmed pertussis. VEs were estimated using a logistic regression model with inverse probability of treatment weighting.

**Results:**

All vaccinated mothers (98 of 404) received aP_gen_ (94% for standalone aP_gen_). The VE of the maternal aP_gen_ vaccination against symptomatic-PCR confirmed pertussis was 95.3% (95% CI: 64.7%, 99.4%; *p* = 0.003). A post-hoc E-value sensitivity analysis indicated that it was unlikely that any residual or unmeasured confounders could explain away the statistically significant VE suggesting a robustness of the observed VE.

**Conclusion:**

Maternal aP_gen_ vaccination during pregnancy was highly likely to provide robust VE against symptomatic-PCR confirmed pertussis in infants during the first three months of life. These findings support the maternal pertussis vaccination with recombinant aP vaccines as effective strategy in Thailand and could be considered for other regions.

**Supplementary Information:**

The online version contains supplementary material available at https://doi.org/10.1007/s15010-026-02868-x.

## Introduction

Pertussis, also referred to as whooping cough, poses a significant threat to infants too young to receive primary immunization, who face the highest risk of severe complications and death [1, 2]. Maternal vaccination is the primary strategy for protecting neonates from infectious diseases during the early months of life [3]. Administering a pertussis vaccine to pregnant women in the second or third trimester boosts maternal immunoglobulin G (IgG) antibodies, which are transplacentally transferred to the fetus and provide passive immunity to bridge the gap from the day of birth [4–9].

Acellular pertussis (aP) vaccination during pregnancy has been endorsed by the World Health Organization and was implemented in several countries to reduce pertussis-related morbidity and mortality among young infants in 2015 [10]. In 2023, Thailand integrated maternal aP vaccination into its national practice guidelines [11]. The aP vaccines in the Thai market are the imported aP vaccines containing chemically detoxified pertussis toxin (aP_chem_) and aP_gen_, a two-component vaccine containing 5 µg of recombinant, genetically detoxified pertussis toxin (PT) and 5 µg of filamentous hemagglutinin (FHA), manufactured locally which is licensed as a monovalent aP vaccine (standalone aP_gen_ or Pertagen^®^) or part of a combined tetanus-diphtheria-pertussis vaccine (TdaP_gen_ or Boostagen^®^) [12].

A previous study showed that pregnant women receiving the aP_gen_ vaccine had significantly higher concentrations of anti-PT antibodies compared with those receiving the aP_chem_ vaccine. Similarly, infants born to mothers receiving the aP_gen_ vaccine exhibited significantly higher anti-PT antibody concentrations at birth and at 2 months than those born to mothers receiving the aP_chem_ vaccine [13]. While the traditionally used aP_chem_ vaccines given during pregnancy have demonstrated effectiveness in reducing the incidence of neonatal pertussis in observational studies [5, 14, 15], the clinical implications of the higher titers elicited by the aP_gen_ vaccine warrant further investigation, particularly given that a definitive correlate of protection has not yet been established [13]. Furthermore, no significant adverse events were reported, supporting a favorable safety profile for the aP_gen_ vaccine [13–16].

In mid-2023, Thailand’s National Health Security Office integrated maternal aP vaccination into the Universal Health Coverage scheme [16]. Thailand is the first country in the world to introduce a pertussis-only (standalone aP) vaccine for use during pregnancy. Within the public sector practically implemented later in 2024, the aP_gen_ (Pertagen^®^) vaccine is administered during pregnancy either alone or with the tetanus-diphtheria vaccine as indicated by the individual’s vaccination history and national guidelines, whereas private prenatal clinics typically provide a single dose of the TdaP_gen_ (Boostagen^®^) [12]. While the vaccine effectiveness (VE) of the aP_chem_ vaccine during pregnancy has been extensively demonstrated globally [1, 4, 5, 9, 17, 18], but the real-world VE in Thailand where the aP_gen_ and aP _chem_ vaccine used in pregnancy for protecting infants remains to be elucidated [13].

Between September 2023 and October 2024, a pertussis outbreak occurred in Narathiwat Province, located in the Deep South of Thailand [19–22]. This region presents a unique sociopolitical landscape where vaccine hesitancy is exceptionally high, largely driven by concerns of potential adverse effects associated with the whole-cell pertussis vaccine used in the national vaccination schedule [21]. Despite assurances from healthcare professionals, local perceptions persist that vaccine-related illnesses could lead to functional impairments and subsequent loss of income [21, 23]. Consequently, between September 2023 and May 2024, over 70% of children under the age of 1 year remain completely unvaccinated against pertussis. Furthermore, the coverage of maternal aP vaccination has never been formally evaluated, suggesting a systemic insufficiency of prenatal immunization [19].

Therefore, this study aimed to evaluate the VE of maternal pertussis vaccination during pregnancy in protecting infants during their first three months of life during an active outbreak in Narathiwat Province, Thailand. The primary and secondary objectives were to determine VE against symptomatic pertussis and laboratory-confirmed pertussis, respectively. To the best of our knowledge, this study represents the first global evaluation of this vaccine technology in real-world settings and provides a critical context for measuring clinical protection [24, 25].

## Methods

### Ethics statements

This study was approved by the Human Research Ethics Committee of Prince of Songkla University (PSU) (approval number REC 68-383-18-1) and conducted in accordance with the Declaration of Helsinki. All participants provided informed consent after receiving a comprehensive briefing on the study objectives prior to enrollment. Each participant received a stipend of 200 baht to compensate for their time.

### Study setting

During the outbreak, aP vaccination during pregnancy was provided as one of three-pronged campaign of the Department of Disease Control. According to the national clinical guidelines of Thailand, the aP vaccine is recommended for pregnant individuals as a single dose administered from 16 weeks of gestation onward [11]. Subsequently, infants follow the national Expanded Programme on Immunization (EPI) in Thailand, receiving the combined diphtheria, tetanus, and whole-cell pertussis (DTwP) vaccine at 2, 4, and 6 months of age. Alternatively, parents may opt for the diphtheria, tetanus, acellular pertussis (DTaP) vaccine through private healthcare facilities [26].

Active surveillance was conducted in the Narathiwat Province between September 2023 and October 2024 during a pertussis outbreak [19–22]. All infants under 3 months of age presenting with rhinorrhea, cough, inspiratory whooping, or apnea underwent nasopharyngeal swabbing. While infants presenting only with rhinorrhea were not classified as emergent cases requiring immediate assessment, they underwent diagnostic testing if they had a history of exposure to patients with confirmed pertussis within 24 h of the index patient being identified. Specimens were transported using Regan–Lowe medium to the Regional Medical Sciences Center 12 Songkhla for testing.

Pertussis cases were laboratory confirmed using an in-house real-time polymerase chain reaction (PCR) assay targeting the *IS481*, *hIS1001*, and *pIS1001* sequences. DNA amplification was performed using the CFX96 Real-Time System (Bio-Rad, Hercules, CA, USA). A sample was defined as positive based on a positive result for *IS481* concurrent with negative results for *hIS1001* and *pIS1001*. The assay demonstrated a sensitivity of 87.5 (95% CI 80.2–93.1) and a specificity of 99.7% (95% CI 99.4–100) compared with the culture gold standard. Notably, this assay did not include testing for the ptxS1 (pertussis toxin subunit S1) gene.

All laboratory findings were recorded in the Digital Disease Surveillance (DDS) database managed by the Division of Epidemiology, Department of Disease Control, under the Ministry of Public Health of Thailand [22]. All laboratory-confirmed cases required supplementary reports to evaluate the onset of illness, symptomatic presentation, and clinical outcomes. This protocol was mandatory across all health jurisdictions in the Narathiwat Province, with a requirement to report results to the DDS within one week. The responsible health personnel were required to submit “No Case Notification” during periods of no detected activity to reconfirm the absence of cases to and ensure the integrity of the surveillance data [27].

### Study design

This study employed a case-control design utilizing two distinct control groups: concurrent community control and test-negative control. The symptomatic PCR-confirmed cases and symptomatic test-negative controls were identified and retrieved from the DDS database. In contrast, the concurrent controls were selected from the community based on the specific geographical and temporal parameters of each case. Specifically, these controls were defined as infants with no history of illness at all during the first 3 months after birth, born in the same month, and residing in the same district as the index case. The community control group was recruited in a 1:10 matching ratio to maximize statistical power.

The VE of the pertussis vaccination during pregnancy in preventing symptomatic-PCR confirmed pertussis was estimated by comparing maternal aP_gen_ vaccination status between the symptomatic laboratory-confirmed cases and concurrent asymptomatic community controls. Additionally, symptomatic laboratory-confirmed cases were compared with test-negative controls to preliminary evaluate the VE against laboratory-confirmed pertussis infection.

### Participant recruitment for cases and controls

Infant participants were categorized into three groups: Symptomatic PCR-confirmed cases, asymptomatic concurrent community controls, and symptomatic test-negative controls. Cases comprised infants aged 0–3 months with symptomatic PCR-confirmed pertussis across all hospitals in Narathiwat Province between September 2023 and October 2024. Utilizing hospital records from the same facility, community controls with no history of illness at all during their first 3 months of life were matched to each case by district, birth month, and birth hospital. A case-to-control ratio of 1:10 was employed; in instances where more than 10 eligible controls were identified within a district, the 10 infants with birth dates closest to the case were selected. Test-negative controls comprised infants who presented with symptoms meeting the suspected symptomatic pertussis but subsequently yielded negative PCR results.

The at-risk date for symptomatic PCR-positive cases and test-negative controls was defined as the 14 days preceding symptom onset. The index date determining the at-risk date for each community-matched control was assigned to match with the specific onset-anchored index date of their corresponding symptomatic PCR-confirmed case. The at-risk date was categorized into four distinct at-risk periods as quarters of the year: Q4-2023, Q1-2024, Q2-2024, and Q3-2024.

Because the participants were infants, data were collected via structured interviews with their parents, who served as the primary respondent. The mother was prioritized as the respondent; however, the father was interviewed if the mother screened positive for postpartum depression (Edinburgh Postnatal Depression Scale score > 6) [28]. After the primary respondent was identified, written informed consent was obtained from the parent, which included permission to collect interview data and authorized the study team to review and record their medical data.

### Data collection

We interviewed the parents of each infant to identify where the mother received prenatal care and to confirm that the infant had no illness at all during their first three months of life. These interviews also provided data on maternal, infant, and household characteristics. Specifically, we collected data on the infant’s age in months during the outbreak, sex, underlying medical conditions, vaccination status during the first 3 months of life, gestational age at birth, birth weight, mode of delivery, and breastfeeding status. We also recorded the timing of prenatal care initiation, maternal influenza vaccination status, maternal age, parity, household exposure to smoking or vaping, average household income, and presence of school-aged siblings at home. All data were collected and managed using an online questionnaire via REDCap (version 14.0.32) [29], hosted by the Faculty of Medicine, PSU (Supplementary Appendix 2).

### Maternal aP vaccination confirmation

We conducted site visits at each prenatal care facility to verify maternal aP vaccination status. Medical records were reviewed to determine if mothers had received pertussis vaccination during pregnancy and what vaccine they received: the standalone aP_gen_, or TdaP_gen_ or Tdap_chem_ vaccines or were unvaccinated. The receipt of either aP_gen_ or TdaP_gen_ was categorized as maternal aP_gen_ vaccination. Additionally, we recorded the specific timing of vaccination prior to delivery. We cross-referenced the data for mothers vaccinated in other provinces using the “Pink Book” held by the participants. This document is the national standard maternal and child health handbook in Thailand and serves as the official record for immunizations and maternal care [30].

### Statistical analysis

Participant characteristics are summarized using descriptive statistics across the case and control groups. We utilized inverse probability treatment weighting (IPTW) using the generalized boosted model to balance the at-risk period and characteristics of the maternal aP vaccinated and unvaccinated groups and address potential confounding [31, 32], or differential access to vaccination. Weights were stabilized to ensure the robustness of the estimates, and models were tuned to minimize the standardized mean difference (SMD) across all variables [33]. SMD < 0.1 was defined as the threshold for achieving an acceptable balance of characteristics between the groups [34]. We generated a histogram to examine the distribution of the estimated IPTW and evaluate its performance. We specifically assessed the presence of extreme weights, defined as any stabilized weight value exceeding 10 [35].

Subsequently, we employed weighted logistic regression analysis to compare symptomatic PCR-positive cases versus asymptomatic community controls and symptomatic cases versus symptomatic test-negative controls. The model incorporated IPTW to adjust for confounders, resulting in weighted odds ratios (wORs). VEs were then estimated using the formula (1-wOR)×100% [36].

We also performed a supplemental analysis to characterize clinical presentations and outcomes, including pneumonia, the requirement for intubation, intensive care unit (ICU) admission, and mortality, among the confirmed cases. This analysis was stratified by age and vaccination status for infants under 3 months of age.

All analyses were performed using the epiDisplay (version 3.5.0.2) [37], WeightIt (version 1.0.0) [38], and tidyverse (version 1.3.1) [39] packages in R language and environment version 4.1.1 (R Foundation for Statistical Computing, Vienna, Austria). Statistical significance was established at a two-sided *P* < 0.05.

### Sample size

Sample size calculation was based on the reported VE of maternal aP vaccination against infant pertussis, estimated to be approximately 75% (pooled value in the literature) [1, 4, 5, 9, 17, 18]. The probability of pertussis exposure among children aged 0–3 months in the Narathiwat Province during the outbreak was approximately 40% [19], corresponding to an expected odds ratio (OR) of 0.25 for pertussis in infants born to vaccinated mothers compared with those born to unvaccinated mothers. Assuming a significance level (α) of 0.05, statistical power of 80%, and case-to-control ratio of 1:10, the required sample size was calculated to be 25 cases and 250 controls based on the formula proposed by Parker (2005) [40]. We sought to recruit the maximum possible number of symptomatic PCR-confirmed cases to ensure adequate statistical power. Consequently, 33 cases were enrolled alongside 330 asymptomatic community controls.

A post-hoc power analysis was performed to evaluate the statistical robustness of the findings. We calculated the effect size using Kish formula to account for small-sample estimation inflation [41, 42]. Subsequently, statistical power was estimated based on the effect size using the Wald-test analytical approximation for logistic regression [43]. If the power falls below 80%, the study conclusions and corresponding sensitivity analyses will focus mainly on the lower bound of the 95% CI rather than the point estimate. This approach serves to mitigate the risk of overestimation resulting from small-sample estimation inflation.

### Sensitivity analysis

Sensitivity evaluation using E-value was performed as a post-hoc analysis. We calculated the E-value and non-null E-value and plotted their corresponding curves to evaluate the potential impact of residual or post-vaccination confounding [44]. The non-null E-value in this study represents the minimum strength of confounding required to explain a VE > 50%. We then performed multivariable logistic regression to assess the association between covariates that remained unbalanced after IPTW and maternal aP vaccination status and being an symptomatic PCR-confirmed pertussis case. The adjusted odds ratios (aORs) for these unbalanced variables were plotted against the E-value curves. Any variable plotting above the curve indicated a confounder strong enough to explain the observed protective effect of the aP_gen_ vaccine [44]. Consequently, such variables were adjusted for in a final multivariable logistic regression model to control for residual confounding, from which the VE was re-estimated as 1-aOR.

Given that the specificity of the PCR assay approached 100%, we assumed no false-positive result. However, the sensitivity was 87.5% and the corresponding false-negative rate was 12.5%, approximately 5 true symptomatic cases were estimated to have been misclassified into the symptomatic test-negative control group. Therefore, we simulated all possible scenarios where 5 randomly selected individuals from the test-negative control group were reclassified into the symptomatic case group and compared against the asymptomatic community controls to address this potential outcome misclassification. We then re-estimated the VE against symptomatic pertussis using aORs derived from a multivariable logistic regression model. The model was adjusted for confounding characteristic variables that exhibited imbalance in the unweighted sample, defined by a SMD > 0.1 prior to IPTW for comparison between symptomatic cases and asymptomatic community-matched controls. Subsequently, the estimates across all simulated scenarios were pooled using Rubin’s rules [45], stratified by number of infants born to vaccinated mother.

## Results

### Patient characteristics

A total of 404 participants were categorized into three distinct cohorts: 33 cases, 330 community-matched controls, and 41 test-negative controls (Table 1). Most mothers were aged between 20 and 34 years (77.5%) and accessed prenatal care at public health facilities (94.3%) in the first trimester. Notably, a marked disparity was observed in maternal pertussis vaccination rates, with 25.7% (*n* = 85) infants in the community-control group and 29.3% (*n* = 12) in the test-negative control group born to mothers having received pertussis vaccine during pregnancy compared to only 3.0% of the cases (*n* = 1). None of the vaccinated mothers received the aP_chem_ vaccine. No mothers were living with human immunodeficiency virus, and only one mother in the community control group had diabetes mellitus before pregnancy. Underlying diseases were omitted from Table 1 as no other participants presented with comorbidities.

**Table 1:** Maternal, infant, and household characteristics for the case, community-matched control, and negative control groups

Characteristics	Case (*n* = 33)	Community-control (*n* = 330)	Negative control (*n* = 41)	Total (*N* = 404)
	*n* (%)	*n* (%)	*n* (%)	*N* (%)
Maternal				
Maternal age at start of pregnancy (years)				
<20	1 (3.0)	24 (7.3)	2 (4.9)	27 (6.7)
20–34	24 (72.8)	254 (77.0)	35 (85.3)	313 (77.5)
≥35	8 (24.2)	52 (15.7)	4 (9.8)	64 (15.8)
Parity				
0	3 (9.1)	57 (17.3)	16 (39.0)	76 (18.8)
1	16 (48.5)	115 (34.8)	14 (34.1)	145 (35.9)
2	9 (27.3)	72 (21.8)	7 (17.1)	88 (21.8)
3	1 (3.0)	48 (14.6)	2 (4.9)	51 (12.6)
≥ 4	4 (12.1)	38 (11.5)	2 (4.9)	44 (10.9)
Initiation trimester of prenatal care				
First	21 (63.6)	229 (69.4)	26 (63.4)	276 (68.3)
Second	12 (36.4)	87 (26.4)	13 (31.7)	112 (27.7)
Third	0 (0)	14 (4.2)	2 (4.9)	16 (4.0)
Type of health service provider for prenatal care				
Public facility	31 (93.9)	314 (95.2)	36 (87.8)	381 (94.3)
Private clinic	2 (6.1)	16 (4.8)	5 (12.2)	23 (5.7)
Maternal aP receipt				
Not received	32 (97.0)	245 (74.3)	29 (70.7)	306 (75.8)
Standalone aP_gen_ (Pertagen)	1 (3.0)	80 (24.2)	11 (26.9)	92 (22.7)
TdaP_gen_ (Boostagen)	0 (0)	5 (1.5)	1 (2.4)	6 (1.5)
Maternal flu vaccine receipt	14 (42.4)	187 (56.7)	22 (53.7)	223 (55.2)
Gestational age at Maternal aP_gen_ vaccine receipt (weeks)				
Not received	32 (97.0)	245 (74.3)	29 (70.7)	306 (75.8)
24–31 (early)	1 (3.0)	42 (12.7)	5 (12.2)	48 (11.8)
≥32 (middle to later)	0 (0)	43 (13.0)	7 (17.1)	50 (12.4)
Gestational age at birth (weeks)				
<37	4 (12.1)	29 (8.8)	6 (14.6)	39 (9.7)
37–41	29 (87.9)	297 (90.0)	35 (85.4)	361 (89.3)
>41	0 (0)	4 (1.2)	0 (0)	4 (1.0)
Type of delivery				
Vaginal	23 (69.7)	284 (86.1)	26 (63.4)	333 (82.4)
Cesarean section	10 (30.3)	46 (13.9)	15 (36.6)	71 (17.6)
Lactation				
Breast	18 (54.5)	216 (65.4)	21 (51.2)	255 (63.1)
Formula	5 (15.2)	31 (9.4)	7 (17.1)	43 (10.7)
Mixed	10 (30.3)	83 (25.2)	13 (31.7)	106 (26.2)
Infant				
Male	19 (57.6)	202 (61.2)	27 (65.9)	248 (61.4)
Age (months)				
1	4 (12.1)	40 (12.1)	13 (31.7)	57 (14.1)
2	7 (21.2)	70 (21.2)	8 (19.5)	85 (21.0)
3	22 (66.7)	220 (66.7)	20 (48.8)	262 (64.9)
Birth weight (g), mean ± SD	3011.8 (462.6)	3083.3 (395.6)	2950.3 (500.3)	3064.0 (414.1)
At-risk period				
Q4-2023	12 (36.4)	139 (42.1)	14 (34.1)	165 (40.8)
Q1-2024	11 (33.3)	92 (27.9)	20 (48.8)	123 (30.5)
Q2-2024	8 (24.2)	79 (23.9)	6 (14.7)	93 (23.0)
Q3-2024	2 (6.1)	20 (6.1)	1 (2.4)	23 (5.7)
Residential area in Narathiwat province during at-risk period				
West zone (high risk)	16 (48.5)	160 (48.5)	23 (56.1)	199 (49.3)
East zone (low risk)	17 (51.5)	170 (51.5)	18 (43.9)	205 (50.7)
Receipt of 1^st^ dose of pertussis vaccine before at-risk date				
None	20 (60.6)	136 (41.2)	19 (46.3)	175 (43.3)
DTaP	1 (3.0)	16 (4.8)	0 (0)	17 (4.2)
DTwP	12 (36.4)	178 (54.0)	22 (53.7)	212 (52.5)
Household				
Household income				
≤ 20 percentile	4 (12.1)	37 (11.2)	1 (2.4)	42 (10.4)
21–40 percentile	12 (36.4)	158 (47.9)	21 (51.3)	191 (47.3)
41–60 percentile	11 (33.3)	99 (30.0)	8 (19.5)	118 (29.2)
61–80 percentile	5 (15.2)	34 (10.3)	11 (26.8)	50 (12.4)
>80 percentile	1 (3.0)	2 (0.6)	0 (0)	3 (0.7)
Number of children in household				
1	5 (15.2)	60 (18.2)	14 (34.1)	79 (19.6)
2	10 (30.3)	116 (35.1)	8 (19.5)	134 (33.1)
3	8 (24.2)	86 (26.1)	13 (31.8)	107 (26.5)
≥ 4	10 (30.3)	68 (20.6)	6 (14.6)	84 (20.8)
School-aged siblings				
0	9 (27.3)	92 (27.9)	19 (46.3)	120 (29.8)
1	10 (30.3)	120 (36.4)	12 (29.3)	142 (35.1)
≥ 2	14 (42.4)	118 (35.8)	10 (24.4)	142 (35.1)
Cohabiting with smoker	20 (60.6)	201 (60.9)	22 (53.7)	243 (60.1)

*aP*_gen,_Standalone recombinant, acellular pertussis vaccine, *DTaP,* pediatric diphtheria, tetanus, and acellular pertussis vaccine, *DTwP,* pediatric diphtheria, tetanus, and whole-cell pertussis, *SD* standard deviation, *TdaP*_gen_combined tetanus-diphtheria-recombinant acellular pertussis vaccine

In total, 20 of the 33 cases **(**60.6%**)** had not received their first dose of the pertussis vaccine before the at-risk date, compared with 41.2% of the community controls. Two of the unvaccinated individuals (10%) were only one month of age and thus had not yet reached the scheduled age for pertussis immunization according to the national EPI. The remaining 18 cases (90%) remained unvaccinated due to parental refusal. Among the 98 infants born to mothers receiving the maternal aP_gen_ vaccine, 48 (49.0%) were vaccinated at the gestational age 24–30 weeks and 50 (51.0%) at age > 31 weeks. The minimum interval between vaccination and birth was 4 weeks. The interval between maternal vaccination and symptom onset was 18 weeks in the only infant born to a vaccinated mother. In the cohort, 13 (13.3%) infants were preterm, including 1 (1.0%) at 35 weeks and 12 (12.2%) at 36 weeks.

Most households (60.1%) included a cohabiting smoker, and a substantial portion of the population fell within the 21–40 income percentile. Additionally, 42.4% of the cases resided in households with two or more school-aged siblings.

### Balance of covariates

As detailed in Table 2, baseline covariates, including the at-risk period, residential area, and lactation status, demonstrated significant imbalance between maternal vaccinated and unvaccinated participants during the evaluation of VE against symptomatic pertussis. Similarly, Table 3 indicates that the at-risk period, residential area, infant age, infant sex, preterm birth, maternal influenza vaccination during pregnancy, type of prenatal clinic, and the number of children in the household were poorly balanced when assessing VE against PCR-confirmed pertussis. Therefore, a sensitivity analysis using E-values was conducted to evaluate the potential impact of this imbalance variables on the observed findings. Diagnostic assessments of the weighting process, illustrated in Online Resource 1 and Online Resource 2, confirm the stability of the model. These figures indicate an absence of extreme weights derived from the GBM estimation, meaning that the estimated effects of maternal aP_gen_ vaccination were not driven by a small number of influential observations, which maintains the robustness of the comparative results between the case and control groups.

**Table 2:** Comparison of absolute SMDs before and after IPTW for maternal acellular pertussis vaccination status among cases and community controls

Variables	Pre-weighting			Post-weighting (pseudo-population)		
	Receipt of maternal aP_gen_ vaccination (*n* = 86)	Non-receipt of maternal aP_gen_ vaccination (*n* = 277)	SMD	Receipt of maternal aP_gen_ vaccination (*n* = 49.5)	Non-receipt of maternal aP_gen_ vaccination (*n* = 260.3)	SMD
	n (%)	n (%)		n (%)	n (%)	
**Maternal**						
Age at start of pregnancy (years)			0.001			0.017
< 20	6 (7.0)	19 (6.9)		2.6 (5.2)	18.0 (6.9)	
20–34	70 (81.4)	208 (75.1)		41.7 (84.2)	197.1 (75.7)	
≥ 35	10 (11.6)	50 (18.0)		5.2 (10.6)	45.2 (17.4)	
Initiation trimester of prenatal care			0.073			0.014
First	64 (74.4)	186 (67.1)		34.1 (68.9)	175.8 (67.5)	
Second	21 (24.4)	78 (28.2)		15.0 (30.3)	73.0 (28.1)	
Third	1 (1.2)	13 (4.7)		0.4 (0.8)	11.5 (4.4)	
Type of health service provider for prenatal care			0.103			0.052
Public facility	75 (87.2)	270 (97.5)		45.0 (90.8)	250.1 (96.1)	
Private clinic	11 (12.8)	7 (2.5)		4.5 (9.2)	10.2 (3.9)	
Maternal flu vaccine receipt	58 (67.4)	143 (51.6)		28.5 (57.5)	137.2 (52.7)	0.048
Gestational age at birth (weeks)			0.018			< 0.001
< 37	9 (10.5)	24 (8.7)		4.5 (9.2)	23.6 (9.1)	
37–41	76 (88.3)	250 (90.2)		44.4 (89.6)	233.7 (89.8)	
> 41	1 (1.2)	3 (1.1)		0.6 (1.2)	3.0 (1.1)	
Type of delivery			0.042			0.014
Vaginal	70 (81.4)	237 (85.6)		41.6 (84)	222.2 (85.4)	
Cesarean section	16 (18.6)	40 (14.4)		7.9 (16)	38.1 (14.6)	
Lactation			0.176			0.141
Breast	67 (77.9)	167 (60.3)		38.0 (76.7)	163.0 (62.6)	
Formula	8 (9.3)	28 (10.1)		4.5 (9.2)	25.7 (9.9)	
Mixed	11 (12.8)	82 (29.6)		7.0 (14.1)	71.6 (27.5)	
** Infant**						
Male	53 (61.6)	168 (60.6)	0.010	29.1 (58.7)	159.6 (61.3)	0.026
Birth weight (g), mean±SD	3058.7±359.2	3058.7±359.2	0.061	3043.6±340.7	3071.6±410.2	0.072
Age (months)			0.009			0.007
1	11 (12.8)	33 (11.9)		5.6 (11.3)	31.3 (12)	
2	6 (7.0)	71 (25.6)		3.8 (7.7)	59.4 (22.8)	
3	69 (80.2)	173 (62.5)		40.1 (81.0)	169.6 (65.2)	
At-risk period			0.265			0.168
Q4-2023	37 (43.0)	114 (41.1)		21.7 (43.9)	111.9 (43.0)	
Q1-2024	7 (8.1)	96 (34.7)		6.6 (13.3)	78.4 (30.1)	
Q2-2024	28 (32.6)	59 (21.3)		15.9 (32.1)	58.9 (22.6)	
Q3-2024	14 (16.3)	8 (2.9)		5.3 (10.7)	11.1 (4.3)	
Residential area in Narathiwat province during at-risk period			0.239			0.102
West zone (high risk)	26 (30.2)	150 (54.2)		20.2 (40.8)	132.9 (51.0)	
East zone (low risk)	60 (69.8)	127 (45.8)		29.3 (59.2)	127.4 (49.0)	
Receipt of 1^st^ dose of pertussis vaccine before at-risk period			0.031			0.025
None	36 (41.9)	120 (43.3)		22.3 (45)	109.5 (42.1)	
DTaP	2 (2.3)	15 (5.4)		1.3 (2.6)	13.3 (5.1)	
DTwP	48 (55.8)	142 (51.3)		25.9 (52.4)	137.5 (52.8)	
**Household**						
Income			0.026			0.040
≤ 20 percentile	8 (9.3)	33 (11.9)		4.1 (8.2)	31.8 (12.2)	
21–40 percentile	46 (53.5)	124 (44.8)		30.2 (61)	119.6 (46)	
41–60 percentile	21 (24.4)	89 (32.1)		10.1 (20.3)	80.8 (31)	
61–80 percentile	11 (12.8)	28 (10.1)		5.1 (10.5)	25.7 (9.9)	
> 80 percentile	0 (0)	3 (1.1)		0 (0)	2.4 (0.9)	
Number of children			0.052			0.057
1	12 (14.0)	53 (19.1)		6.2 (12.6)	47.7 (18.3)	
2	36 (41.9)	90 (32.5)		18.6 (37.6)	88.1 (33.9)	
3	25 (29.1)	69 (24.9)		14.1 (28.4)	65.4 (25.1)	
≥ 4	13 (15.0)	65 (23.5)		10.6 (21.4)	59.1 (22.7)	
School-aged siblings			0.060			0.083
0	20 (23.3)	81 (29.2)		10 (20.2)	74.2 (28.5)	
1	37 (43.0)	93 (33.6)		20.8 (42.1)	91 (35)	
≥ 2	29 (33.7)	103 (37.2)		18.7 (37.7)	95.1 (36.5)	
Cohabiting with smoker	54 (62.8)	167 (60.3)	0.025	33.3 (67.3)	160.8 (61.8)	0.055

aP_gen_ standalone recombinant, acellular pertussis vaccine, *DTaP, pediatric* diphtheria, tetanus, and acellular pertussis vaccine, *DTwP, pediatric* diphtheria, tetanus, and whole-cell pertussis, *IPTW* inverse probability of treatment weighting, *SMD* standardized mean difference

**Table 3:** Comparison of absolute SMDs before and after IPTW for maternal acellular pertussis vaccination status among cases and negative controls

Variables	Pre-weighting			Post-weighting (pseudo-population)		
	Receipt of maternal aP_gen_ vaccination (*n* = 13)	non-receipt of maternal aP_gen_ vaccination (*n* = 61)	SMD	Receipt of maternal aP_gen_ vaccination (*n* = 5.8)	Non-receipt of maternal aP_gen_ vaccination (*n* = 57.7)	SMD
	n (%)	n (%)		n (%)	n (%)	
**Maternal**						
Age at start of pregnancy (years)			0.049			0.047
< 20	0 (0)	3 (4.9)		0 (0)	2.7 (4.7)	
20–34	12 (92.3)	47 (77.1)		5.0 (86.9)	44.7 (77.5)	
≥ 35	1 (7.7)	11 (18.0)		0.8 (13.1)	10.3 (17.8)	
Initiation trimester of prenatal care			0.163			0.17
First	10 (76.9)	37 (60.6)		4.5 (78.1)	35.2 (61)	
Second	3 (23.1)	22 (36.1)		1.3 (21.9)	20.6 (35.7)	
Third	0 (0)	2 (3.3)		0 (0)	1.9 (3.3)	
Type of health service provider for prenatal care			0.165			0.188
Public facility	10 (76.9)	57 (93.4)		4.3 (74.5)	53.8 (93.4)	
Private clinic	3 (23.1)	4 (6.6)		1.5 (25.5)	3.8 (6.6)	
Maternal flu vaccine receipt	8 (61.5)	28 (45.9)		3.4 (58.9)	26.5 (46)	0.129
Gestational age at birth (weeks)			0.209			0.133
< 37	4 (30.8)	6 (9.8)		1.4 (23.7)	6 (10.4)	
37–41	9 (69.2)	55 (90.2)		4.4 (76.3)	51.7 (89.6)	
> 41	0 (0)	0 (0)		0 (0)	0 (0)	
Type of delivery			0.037			0.062
Vaginal	9 (69.2)	40 (65.6)		4.1 (72.1)	38 (65.9)	
Cesarean section	4 (30.8)	21 (34.4)		1.6 (27.9)	19.7 (34.1)	
Lactation			0.014			0.039
Breast	7 (53.8)	32 (52.5)		2.8 (49.1)	30.6 (53.0)	
Formula	2 (15.4)	10 (16.4)		0.9 (15.6)	9.4 (16.3)	
Mixed	4 (30.8)	19 (31.1)		2.0 (35.3)	17.7 (30.7)	
**Infant**						
Male	10 (76.9)	36 (59)	0.179	4.3 (75.3)	34.5 (59.8)	0.155
Age (months)			0.095			0.1
1	4 (30.8)	13 (21.3)		1.8 (30.9)	12.1 (21.0)	
2	1 (7.7)	14 (23)		0.6 (9.8)	13.2 (22.9)	
3	8 (61.5)	34 (55.7)		3.4 (59.3)	32.4 (56.1)	
At-risk period			0.228			0.149
Q4-2023	6 (46.2)	20 (32.8)		2.4 (41.6)	18.8 (32.6)	
Q1-2024	3 (23.1)	28 (45.9)		1.8 (30.6)	26.3 (45.5)	
Q2-2024	3 (23.1)	11 (18.0)		1.3 (22.6)	10.5 (18.3)	
Q3-2024	1 (7.6)	2 (3.3)		0.3 (5.2)	2.1 (3.6)	
Residential area in Narathiwat province during at-risk period			0.201			0.202
West zone (high risk)	9 (69.2)	30 (49.2)		4.0 (69.7)	28.5 (49.5)	
East zone (low risk)	4 (30.8)	31 (50.8)		1.7 (30.3)	29.1 (50.5)	
Receipt of 1^st^ dose of pertussis vaccine before at-risk period			0.016			0.015
None	4 (30.8)	35 (57.4)		2.2 (37.8)	32.5 (56.3)	
DTaP	0 (0)	1 (1.6)		0 (0)	0.9 (1.5)	
DTwP	9 (69.2)	25 (41.0)		3.6 (62.2)	24.3 (42.2)	
**Household**						
Income			0.082			0.08
≤ 20 percentile	0 (0)	5 (8.2)		0 (0)	4.6 (8.0)	
21–40 percentile	6 (46.1)	27 (44.3)		2.5 (43.4)	25.5 (44.1)	
41–60 percentile	3 (23.1)	16 (26.2)		1.5 (25.2)	14.7 (25.5)	
61–80 percentile	4 (30.8)	12 (19.7)		1.8 (31.4)	11.9 (20.6)	
> 80 percentile	0 (0)	1 (1.6)		0 (0)	1.0 (1.8)	
Number of children			0.248			0.216
1	6 (46.2)	13 (21.3)		2.5 (43.1)	13.1 (22.7)	
2	2 (15.4)	16 (26.2)		0.9 (15.5)	15.1 (26.1)	
3	4 (30.8)	17 (27.9)		2.0 (34.5)	15.8 (27.5)	
≥ 4	1 (7.6)	15 (24.6)		0.4 (6.9)	13.7 (23.7)	
School-aged siblings			0.288			0.249
0	8 (61.5)	20 (32.8)		3.4 (59.3)	19.8 (34.4)	
1	2 (15.4)	20 (32.8)		0.8 (13.7)	18.4 (31.8)	
≥ 2	3 (23.1)	21 (34.4)		1.6 (27)	19.5 (33.8)	
Cohabiting with smoker	8 (61.5)	34 (55.7)	0.058	3.6 (63.3)	32.3 (56.0)	0.073

aP_gen_ standalone recombinant, acellular pertussis vaccine, *DTaP* pediatric diphtheria, tetanus, and acellular pertussis vaccine, *DTwP* pediatric diphtheria, tetanus, and whole-cell pertussis, *IPTW* inverse probability of treatment weighting, *SMD* standardized mean difference

### Vaccine effectiveness

Table 4 shows that the VE of aP_gen_ vaccine remained high and consistent across the two control models. In the comparison of cases with community-matched controls, maternal vaccination demonstrated a VE of 95.3% (95% confidence interval [CI]: 64.7%, 99.4%; *p* = 0.003) against symptomatic PCR-confirmed pertussis. Similarly, the comparison of cases with test-negative controls yielded a VE of 95.0% (95% CI: 58.8%, 99.4%; *p* = 0.005) against laboratory-confirmed pertussis infection. The analytical Wald-test approximation yielded a statistical power of 59.7% for the community-matched model and 64.8% for the test-negative model.

**Table 4:** Maternal aP_gen_ vaccine effectiveness estimates for case versus community-matched controls and case versus test-negative controls

Control	Pseudo-population (*n*)				OR (95%CI)	VE (95% CI)	*P* value
	Vaccinated		Unvaccinated				
	Case	Control	Case	Control			
Univariate analysis (actual population)							
Community-matched	1	85	32	245	0.09 (0.01, 0.62)	91.0% (38.3%, 99.1%)	0.019
Test-negative	1	12	32	29	0.08 (0.01, 0.62)	92.4% (38.3%, 99.1%)	0.016
Weighted logistic regression							
Community-matched	0.3	49.2	30.9	229.4	0.05 (0.01, 0.35)	95.3% (64.7%, 99.4%)	0.003
Test-negative	0.8	12.2	26.2	33.8	0.05 (0.01, 0.41)	95.0% (58.8%, 99.4%)	0.005
Weighted logistic regression with multivariable adjustment for residual confounders							
Community-matched^a^	0.3	49.2	30.9	229.4	0.04 (0.01, 0.32)	95.9% (67.8%, 99.5%)	0.002
Test-negative^b^	0.8	12.2	26.2	33.8	0.03 (0.00, 0.54)	96.7% (46.5%, 99.8%)	0.016
Sensitivity analysis for VE against symptomatic pertussis including test-negative controls as cases							
All five false negatives involved infants born to unvaccinated mothers (Possible scenarios = 118,*755)*							
Community-matched^c^	1	85	37	245	0.06 (0.00, 0.49)	93.8% (51.1%, 99.2%)	0.008
One of five false negatives were infants born to vaccinated mothers (Possible scenarios = 285,*012)*							
Community-matched^c^	2	85	36	245	0.13 (0.03, 0.61)	86.7% (38.3%, 97.1%)	0.010
Two of five false negatives were infants born to vaccinated mothers (Possible scenarios = 241,164)							
Community-matched^c^	3	85	35	245	0.21 (0.06, 0.79)	78.6% (21.1%, 94.2%)	0.021
Three of five false negatives were infants born to vaccinated mothers (Possible scenarios = 89,320)							
Community-matched^c^	4	85	34	245	0.31 (0.10, 0.98)	69.4% (1.6%, 90.5%)	0.047
Four of five false negatives were infants born to vaccinated mothers (Possible scenarios = 14,355)							
Community-matched^c^	5	85	33	245	0.41 (0.14, 1.20)	59.1% (− 19.9%, 86.0%)	0.103
All five false negatives were infants born to vaccinated mothers (Possible scenarios = 792)							
Community-matched^c^	6	85	32	245	0.53 (0.20, 1.44)	46.9% (− 44.4%, 80.5%)	0.215

*aP*_gen_ standalone recombinant, acellular pertussis vaccine, *CI* confidence interval, *OR* odds ratio, *VE* vaccine effectiveness

^a^Multivariable logistic regression adjusted for at-risk period

^b^Multivariable logistic regression adjusted for at-risk period, age of infant, prenatal care clinic, and household children

^c^Multivariable logistic regression adjusted for at-risk period, residential area (West versus East zone), and lactation status

### Sensitivity analysis

#### E-value analysis

Online Resource 3 demonstrates that at a 95% CI lower bound E-value of 5.2, no observed confounding variable could explain away the statistically significant VE against symptomatic pertussis. Conversely, a 95% CI lower bound non-null E-value of 2.2 suggested that a VE threshold > 50% could be explained away by residual imbalance of the at-risk period between infants born to vaccinated and unvaccinated mothers. However, Table 4 shows that after re-calculating the VE to adjust for this at-risk period, the VE increased to 95.9% (95% CI: 67.8, 99.5), which indicates that imbalance in the at-risk period previously underestimated the lower bound of VE against pertussis.

Online Resource 4 demonstrates that at a 95% CI lower bound E-value of 4.3, the observed VE against PCR-confirmed pertussis could be explained away by residual imbalances in infant age, number of household children, type of prenatal clinic, and at-risk period. After re-calculating the VE to adjust for these imbalanced covariates, the estimate increased to 96.7% (95% CI: 46.5%, 99.8%); however, the lower bound of the 95% CI remained below 50%, falling just short of the clinically significant threshold for VE against PCR-confirmed pertussis (Table 4).

### Sensitivity analysis for non-differential outcome misclassification

Our sensitivity analysis accounting for potential outcome misclassification demonstrated that the robustness of the VE against symptomatic pertussis depended heavily on the maternal vaccination status of the misclassified cases (Table 4). If all five false-negative individuals were assumed to be infants born to unvaccinated mothers, the VE remained robust, with the lower bound of the 95% CI > 50%. Conversely, if one, two, or three of these false-negative individuals were infants born to vaccinated mothers, the VE estimate remained statistically significant but the lower bound fell below 50%. If four or five of the false-negative individuals were born to vaccinated mothers, the VE estimate was no longer robust.

### Clinical characteristics of the cases

All 33 infants with symptomatic PCR-confirmed pertussis presented with rhinorrhea and had documented exposure to a confirmed pertussis case in the Narathiwat Province. Six infants with isolated rhinorrhea in the absence of other symptoms (aged 2–3 months) did not require inpatient admission. Table 5 illustrates the clinical burden of pertussis among infants born to mothers who did not receive the aP_gen_ vaccine (*n* = 32). In the first month of life, 75% infants required mechanical ventilation and 50% died. Cough was a consistent clinical finding across all age groups. Conversely, the single case involving an infant born to an aP_gen_-vaccinated mother who presented with cough and required hospitalization but did not develop pneumonia, necessitated intensive respiratory support, but did not result in a lethal outcome. The frequency of apnea in intubated and mechanically ventilated infants was not recorded, potentially leading to an underreporting of this condition.

**Table 5:** Clinical presentation and outcomes of laboratory-confirmed pertussis in infants by maternal aP_gen_ vaccination status and infant age

Clinical presentation and outcome	Non-receipt of maternal aP_gen_ vaccination			Receipt of maternal aP_gen_ vaccination
	Age			Age
	1 Month *n* (%)	2 Months *n* (%)	3 Months *n* (%)	3 Months *n* (%)
Total, N	4	7	21	1
Symptoms				
Cough	3 (75%)	2 (29%)	16 (76%)	1 (100%)
Whooping	3 (75%)	2 (29%)	7 (33%)	0 (0%)
Cyanosis	0 (0%)	2 (29%)	2 (10%)	0 (0%)
Apnea*	2 (50%)	0 (0%)	0 (0%)	0 (0%)
Seizure	1 (25%)	0 (0%)	0 (0%)	0 (0%)
Duration from the first symptom onset to hospitalization (days), median (IQR)	6 (4, 9)	5 (3, 6)	6 (3, 7)	10 (N/A)
Clinical outcomes				
Required hospitalization	4 (100%)	6 (86%)	16 (76%)	1 (100%)
Pneumonia	3 (75%)	2 (29%)	7 (33%)	0 (0%)
Required endotracheal intubation and mechanical ventilation	3 (75%)	1 (14%)	3 (14%)	0 (0%)
Intensive care unit admission	2 (50%)	0 (0%)	3 (14%)	0 (0%)
Dead	2 (50%)	0 (0%)	1 (5%)	0 (0%)
Length of hospital stay (days), median (IQR)	7 (2, 14)	2 (1, 8)	1 (1, 4)	4 (N/A)

*aP*_gen_ standalone recombinant, genetically inactivated acellular pertussis vaccine, *N/A* only one value

*Apnea in intubated and mechanically ventilated infants was not recorded; these infants were classified as having no apnea in the percentage calculation

Table 6 shows that among 32 cases born to unvaccinated mothers, outcomes were most severe in the 20 who had not received a primary infant pertussis vaccine dose. In this subgroup, 9 (45%) infants developed pneumonia, 5 (25%) required mechanical ventilation, and 3 (15%) died.

**Table 6:** Clinical presentation and outcomes of laboratory-confirmed pertussis in infants by maternal aP_gen_ vaccination status and receipt of 1^st^ dose of pertussis vaccine before at-risk period

Clinical presentation and outcome	Non-receipt of maternal aP_gen_ vaccination			Receipt of maternal aP_gen_ vaccination
	Receipt of 1st dose of pertussis vaccine			Receipt of 1st dose of pertussis vaccine
	DTaP	DTwP	None	DTwP
Total, N	1	11	20	1
Symptoms				
Cough	1 (100%)	7 (64%)	13 (65%)	1 (100%)
Whooping	0 (0%)	3 (27%)	9 (45%)	0 (0%)
Cyanosis	0 (0%)	4 (36%)	0 (0%)	0 (0%)
Apnea*	0 (0%)	0 (0%)	2 (10%)	0 (0%)
Seizure	0 (0%)	0 (0%)	1 (5%)	0 (0%)
Duration from the first symptom onset to hospitalization (days), median (IQR)	7 (N/A)	5 (4, 6)	6 (4, 8)	10 (N/A)
Clinical outcomes				
Required hospitalization	1 (100%)	9 (82%)	16 (80%)	1 (100%)
Pneumonia	0 (0%)	3 (27%)	9 (45%)	0 (0%)
Required endotracheal intubation and mechanical ventilation	0 (0%)	2 (18%)	5 (25%)	0 (0%)
Intensive care unit admission	0 (0%)	2 (18%)	3 (15%)	0 (0%)
Dead	0 (0%)	0 (0%)	3 (15%)	0 (0%)
Length of hospital stay (days), median (IQR)	1 (N/A)	1 (1, 13)	2 (1, 4)	4 (N/A)

*aP*_gen_ standalone recombinant, acellular pertussis vaccine, *DTaP pediatric* diphtheria, tetanus, and acellular pertussis vaccine, *DTwP, pediatric* diphtheria, tetanus, and whole-cell pertussis, *N/A* only one value

*Apnea in intubated and mechanically ventilated infants was not recorded; these infants were classified as having no apnea in the percentage calculation

## Discussion

In this study, maternal pertussis vaccination during pregnancy with the aP_gen_ vaccine demonstrated a high and consistent VE of approximately 95% of protecting 0–3 months old infants against symptomatic and PCR-confirmed pertussis during an outbreak in Thailand. This is the first study reporting on the effectiveness of maternal aP_gen_ vaccination during pregnancy protecting young infants against pertussis. As pertussis is a notifiable disease in Thailand and active surveillance was employed during the outbreak, we were in the position to conduct this retrospective post-implementation vaccine effectiveness study. This study provides evidence supporting the use of this vaccine in the Thai maternal pertussis vaccination program.

While the high point estimate of a VE of 95% may reflect small-sample inflation, as evidenced by a power of < 80%, the lower bound of the 95% CI remained clinically meaningful, > the 50% threshold. Therefore, an E-value analysis and multivariable logistic regression adjustment for residual imbalance were performed as a post-hoc analysis to evaluate the impact of residual confounding after IPTW. The lower bound of the 95% CI remained above 50% for VE against symptomatic pertussis in the community controls, confirming a robust and clinically significant protective effect [46]. Conversely, the lower bound of the 95% CI for PCR-confirmed pertussis in the test-negative controls just fell below 50% (46.5%), reducing the clinical certainty for this endpoint.

Approximately 30% of the infants in the test-negative control group were born to vaccinated mothers (Table 1). Considering a sensitivity of the pertussis PCR of 87.5%, the most plausible distribution of the potential five false-negative cases would allocate 0–2 cases to the vaccinated group with robust VE (78.6-93.8%) in the same range of VEs observed in other studies [1, 4, 5, 9, 17, 18]. However, scenarios in which three or more false negatives occurred in the vaccinated group (representing 60% or more of the misclassified cases) are highly implausible. Based on these findings, it is unlikely that PCR misclassification completely invalidated the statistically significant VE observed against symptomatic pertussis.

The final adjusted VE against symptomatic PCR-confirmed pertussis of 95.9% (95% CI: 67.8%, 99.5%) for the aP_gen_ vaccine was comparable to the estimates published in the literature for the aP_chem_ vaccine. While the aP_chem_ vaccine has shown significant success globally, meta-analyses and observational studies often report a VE ranging from 69 to 93% [1, 4, 5, 9, 17, 18]. Although unmeasured confounding regarding infection risk may exist, an E-value of 5.2 suggests it is implausible that residual confounding entirely explains away the statistically significant VE against symptomatic pertussis. This indicated a robustness of the observed VE of aP_gen_ vaccination during pregnancy.

This study has some strengths. First, this study was conducted during an active pertussis outbreak in a high-risk region with low baseline maternal and childhood vaccination coverage, which established an environment for evaluating clinical protection. In the absence of these conditions, conducting an effectiveness study would require high numbers of infants tested to get sufficient numbers of cases, while an experimental efficacy trial would present significant ethical challenges, as withholding a recommended vaccine from a control group would be considered ethically impermissible [47]. Second, we conducted sensitivity analyses to assess the robustness of our results regarding confounding and outcome misclassification.

However, one major limitation of the study was the relatively small sample size of confirmed cases (*n* = 33). The post-hoc power analysis indicated that the study was technically underpowered to detect smaller effect sizes, with a calculated power of 59.7% and 64.8% for the asymptomatic community control and symptomatic test-negative control models. Secondly, although the probability of PCR misclassification is low, its potential to attenuate the statistically significant VE against symptomatic pertussis cannot be entirely ruled out. Thirdly, the active surveillance system was predominantly structured to capture clinically evident cases, thereby introducing a potential surveillance bias that may have missed milder, undetected forms of pertussis in the community. If infants born to vaccinated mothers primarily experienced mild symptomatic infections that did not prompt a hospital visit, this differential under-ascertainment would artificially reduce the case count in the vaccinated group, potentially overestimating the true VE. However, mild cases with undetectable symptoms do not pose a significant public health burden, as these infants remain asymptomatic without progressing to severe disease, as evidenced by parental confirmation that the infants experienced no illness at all. Consequently, omitting these undetectable infections does not compromise the clinical relevance of our VE estimates, which focus specifically on preventing the severe symptomatic forms of pertussis that drive public health concern.

Previous clinical trials indicate that TdaP_gen_ and aP_gen_ induce somewhat different geometric mean concentrations or titers for PT and FHA [12, 13]. In this study, these two recombinant aP vaccines were analyzed collectively as the two formulations contain the same pertussis vaccine components and induce comparable immune responses and primarily due to sample size constraints. However, most vaccinated women (94%) received the standalone aP_gen_ vaccine. Consequently, the observed VEs predominantly reflect protection elicited by aP_gen_, which constituted the majority of the sample, rather than TdaP_gen_. Hence, comparing these findings to external immunogenicity data should be interpreted with this in mind.

In conclusion, maternal aP_gen_ vaccination during pregnancy is highly likely to provide robust VE against symptomatic PCR-confirmed pertussis in infants during the first three months of life, demonstrating a VE at least comparable to that reported in other studies of aP_chem_ vaccine. This study supports the use of aP_gen_ vaccine in maternal vaccination programs as implemented by the Thai government in the national immunization schedule after the pertussis outbreak. In countries with domestic manufacturing capabilities for the aP_gen_ vaccine, its utilization in pregnant women offers a viable strategy for vaccine security especially during outbreak.

## Supplementary Information

Below is the link to the electronic supplementary material.

Supplementary Material 1

Supplementary Material 2

## Data Availability

We have provided R codes and de-identified data for all analyses in our study in the form of a Jupyter notebook file. This file can be accessed through Github 48.
