# Supplementary material for: Real-world effectiveness of maternal genetically inactivated acellular pertussis vaccination in protecting infants under three months of age: a case-control study using concurrent community-based and test-negative controls
Source: Infection. 2026 Jul 27;54(5):2469–83. doi: 10.1007/s15010-026-02868-x (PMC13727804; doi:10.1007/s15010-026-02868-x)
Supplement: Supplementary file 1 [file 15010_2026_2868_MOESM2_ESM.pdf]

DEPARTMENT OF EPIDEMIOLOGY
FACULTY OF MEDICINE
PRINCE OF SONGKLA UNIVERSITY

mother_home

โปรดกรอกข้อมูลที่จำเป็นให้ครบ

ก่อนกรอกข้อมูลกรุณาตรวจสอบว่าท่านไม่ได้กรอกผ่าน LINE ซึ่งจะบันทึกข้อมูลไม่ได้ !!!

- 1. ปกติเมื่อแสกน QR code จะปรากฏ link ให้กด ดังภาพ

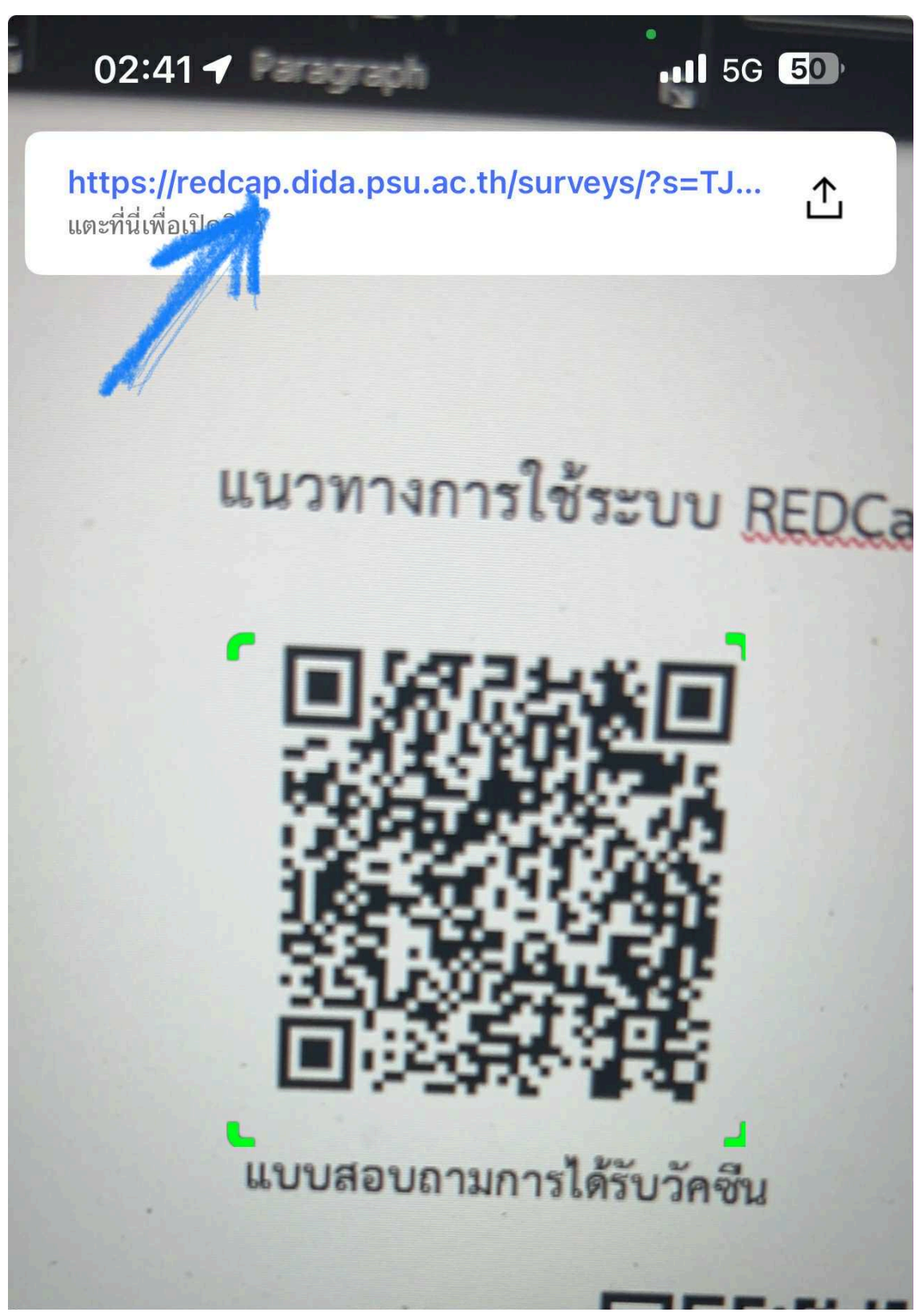

- 2. กดปุ่มจุด 3 จุดขวาล่าง เพื่อเปิดแบบกรอกข้อมูลใน Default browser

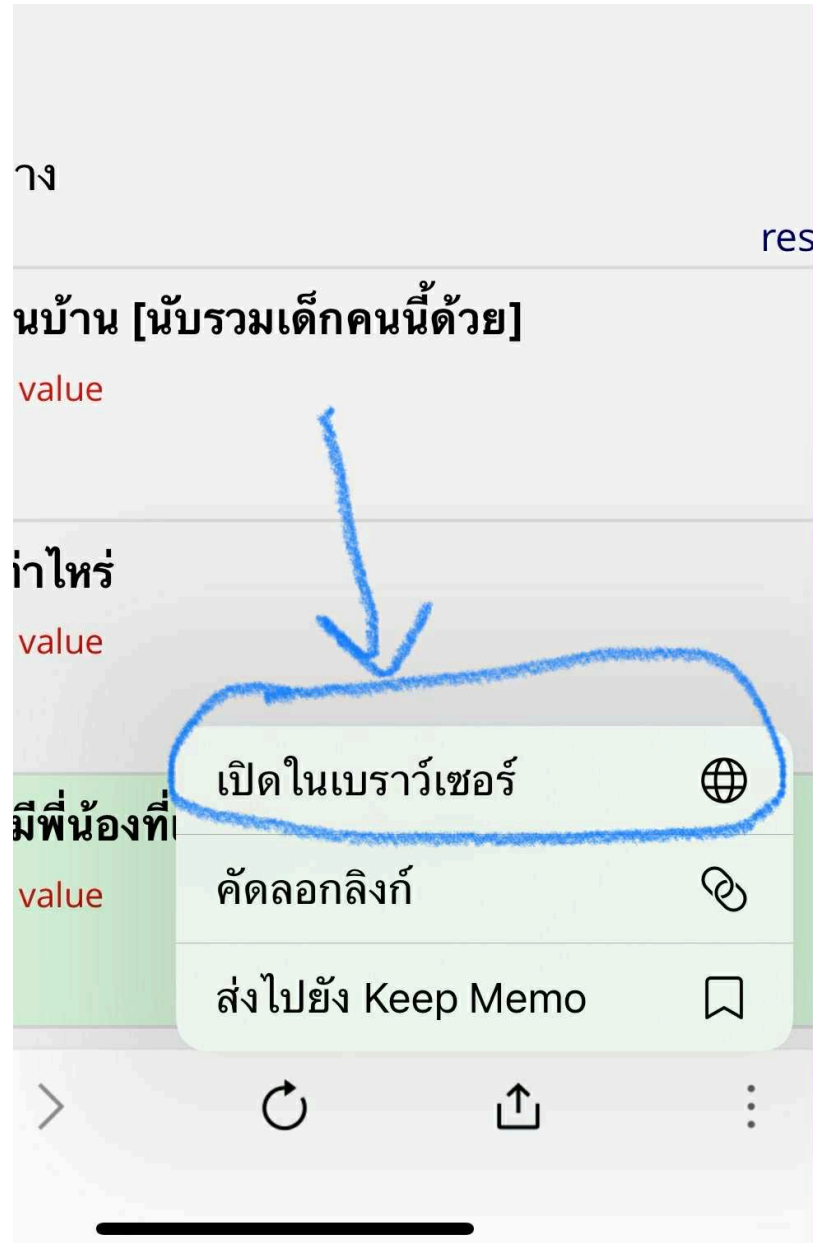

- 3. สำหรับมือถือ
    - # iPhone ควรเปิดใน Safari
    - # Android ควรเปิดใน Chrome
    - **หากไม่ใช่ให้ copy url ไปเปิดใน browser ที่ถูกต้อง**

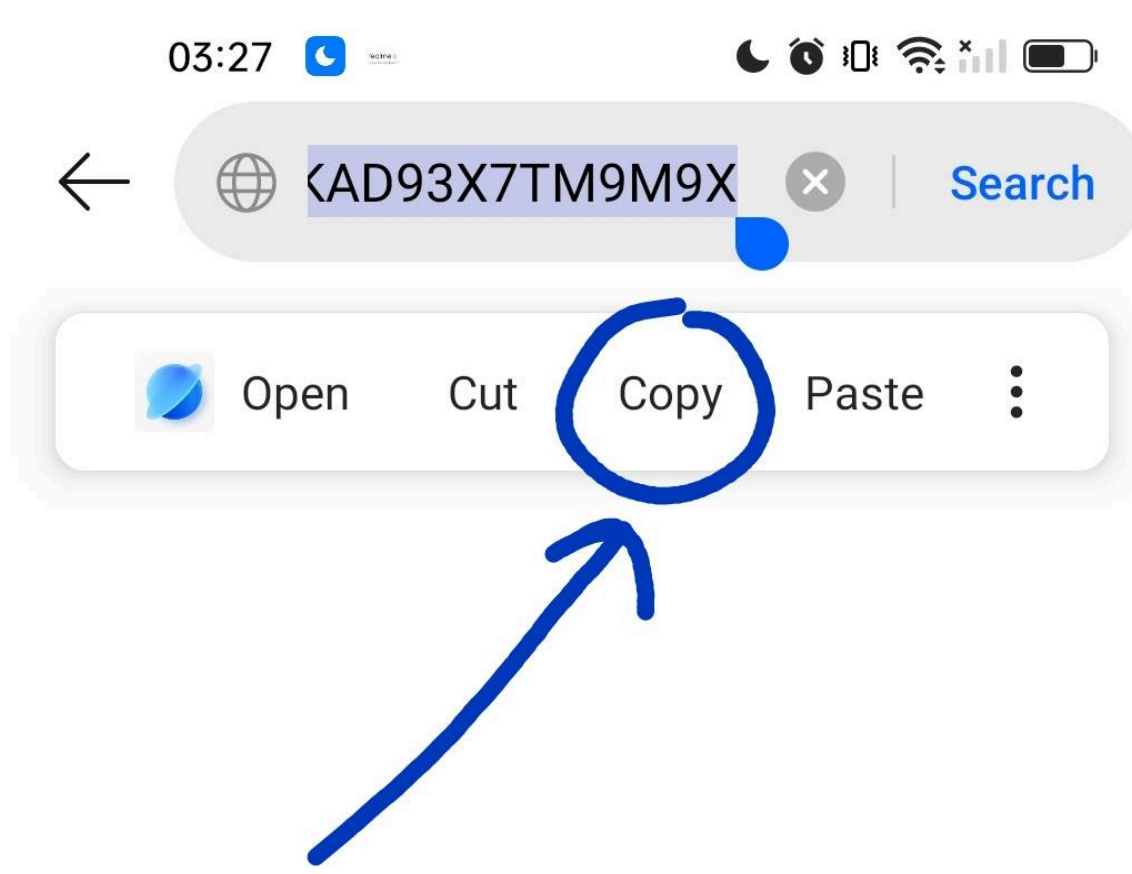

เลือก ID

* must provide value

ผู้ให้ข้อมูลเป็น
* must provide value

- ◯ มารดา
- ◯ บิดา

อายุมารดาตอนคลอดบุตร (กี่ปี)
* must provide value

ตอนคลอดบุตรอายุครรภ์กี่สัปดาห์ [ใส่ 50 หากไม่ทราบ/จำไม่ได้]
* must provide value

วิธีคลอด
* must provide value

- ◯ คลอดเอง
- ◯ ผ่าคลอด

โรคประจำตัวก่อนตั้งครรภ์
* must provide value

- ☐ ไม่มี
- ☐ หอบหืด
- ☐ เบาหวาน
- ☐ ความดันโลหิตสูง
- ☐ โรคหลอดเลือดหัวใจ
- ☐ อื่น ๆ

ได้ฝากครรภ์หรือไม่
* must provide value

- ◯ ไม่ฝาก
- ◯ ฝาก

รายได้ครอบครัวโดยเฉลี่ยต่อเดือนของพ่อแม่ [นับเฉพาะเงินเดือนของพ่อกับแม่รวมกัน]
* must provide value

- ◯ < 5,000 บาท
- ◯ 5,000-9,999 บาท
- ◯ 10,000-19,999 บาท
- ◯ 20,000-49,999 บาท
- ◯ ≥50,000 บาท
- ◯ ไม่ประสงค์จะตอบ

การสูบบุหรี่/บุหรี่ไฟฟ้า ภายในบ้าน
* must provide value

- ◯ ไม่มี
- ◯ มี

บันทึก PDF ก่อนกดปุ่ม submit !!!

# - สำหรับ iPhone

02:45
◀ LINE
5G 49

redcap.dida.psu.ac.th

- (●) ไม่
- ( ) ได้

res

มีสมุดบันทึกการฉีดวัคซีนมั้ย (เล่มสีชมพู)

* must provide value

- (●) ไม่มี
- ( ) มี

res

การให้นมบุตรช่วง 3 เดือนแรกเกิด

* must provide value

- (●) นมแม่
- ( ) นมผง
- ( ) ทั้ง 2 อย่าง

res

มีพี่น้องกี่คนในบ้าน [นับรวมเด็กคนนี้ด้วย]

* must provide value

1

เป็นลูกคนที่เท่าไหร่

* must provide value

1

ตอนแรกเกิด มีพี่น้องที่เข้าโรงเรียนแล้วกี่คน

* must provide value

0

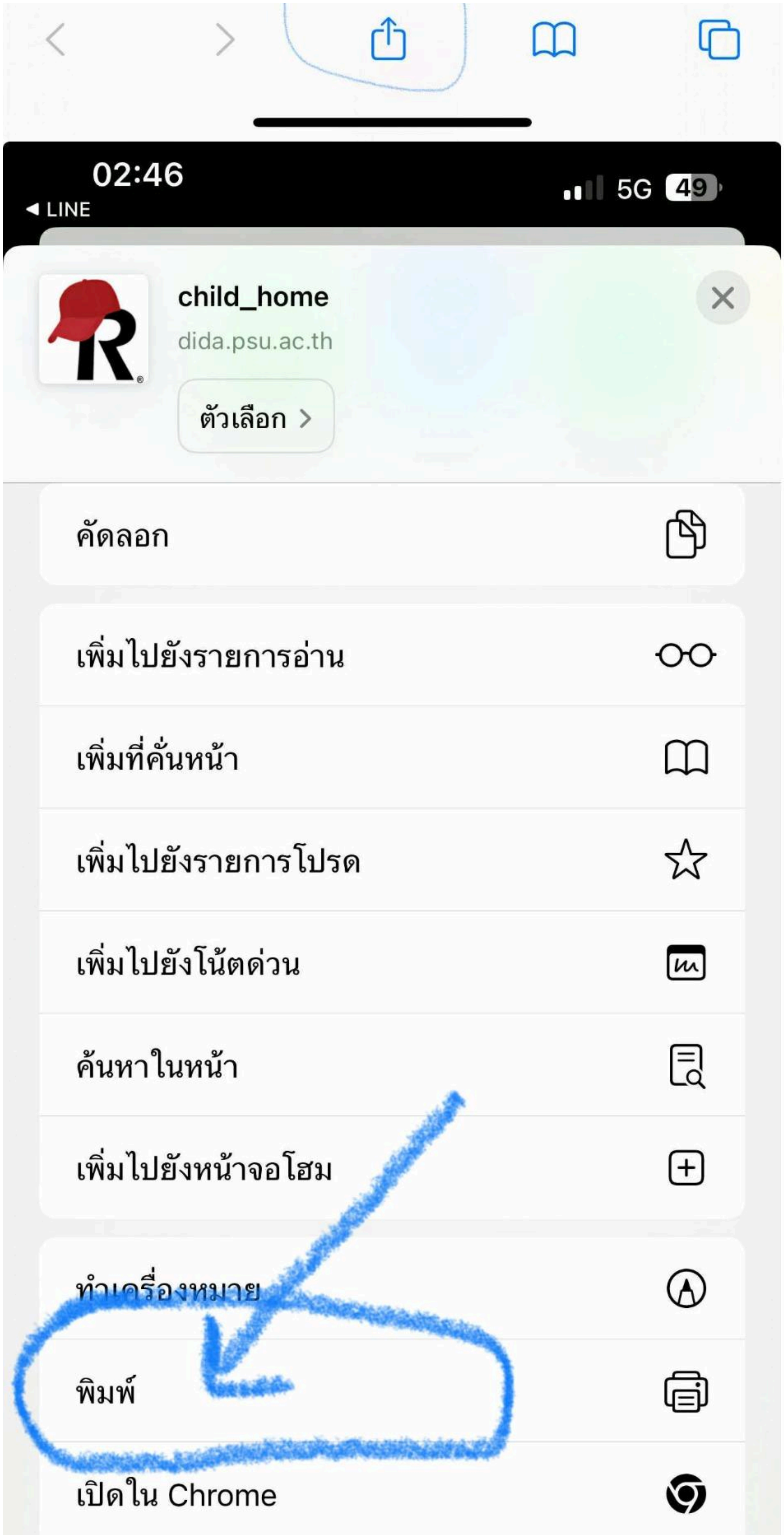
02:46
◀ LINE
5G 49
child_home
dida.psu.ac.th
ตัวเลือก >
คัดลอก
เพิ่มไปยังรายการอ่าน
เพิ่มที่คั่นหน้า
เพิ่มไปยังรายการโปรด
เพิ่มไปยังโน้ตด่วน
ค้นหาในหน้า
เพิ่มไปยังหน้าจอโฮม
ทำเครื่องหมาย
พิมพ์
เปิดใน Chrome

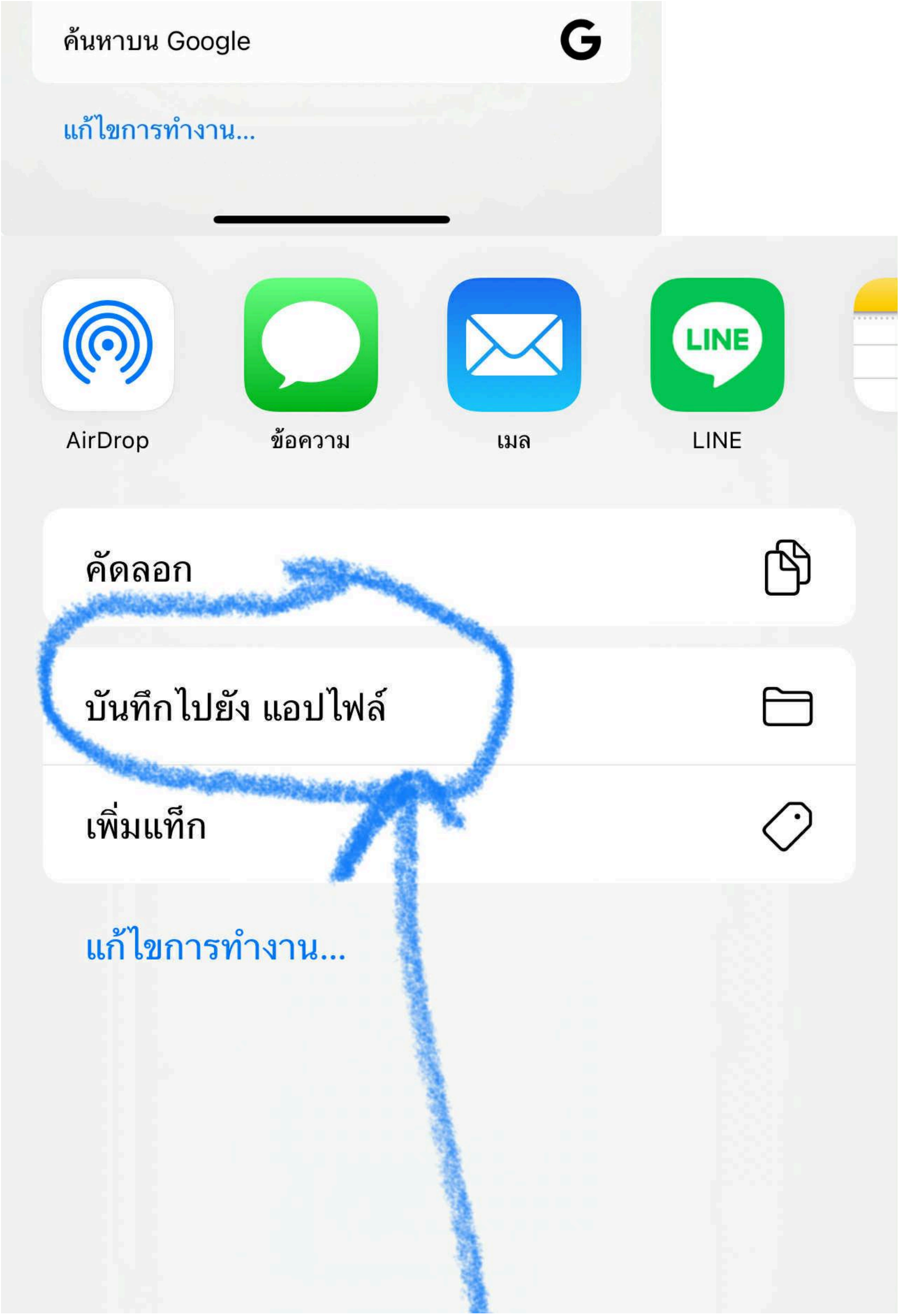
ค้นหาบน Google
แก้ไขการทำงาน...
AirDrop
ข้อความ
เมล
LINE
LINE
คัดลอก
บันทึกไปยัง แอปไฟล์
เพิ่มแท็ก
แก้ไขการทำงาน...

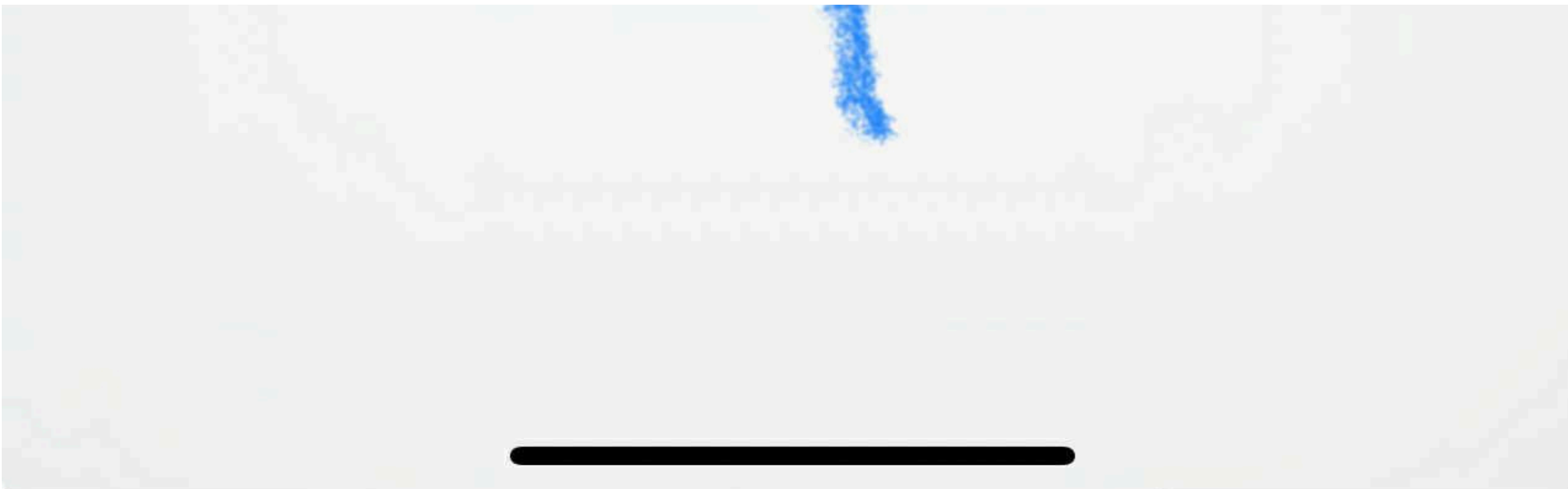

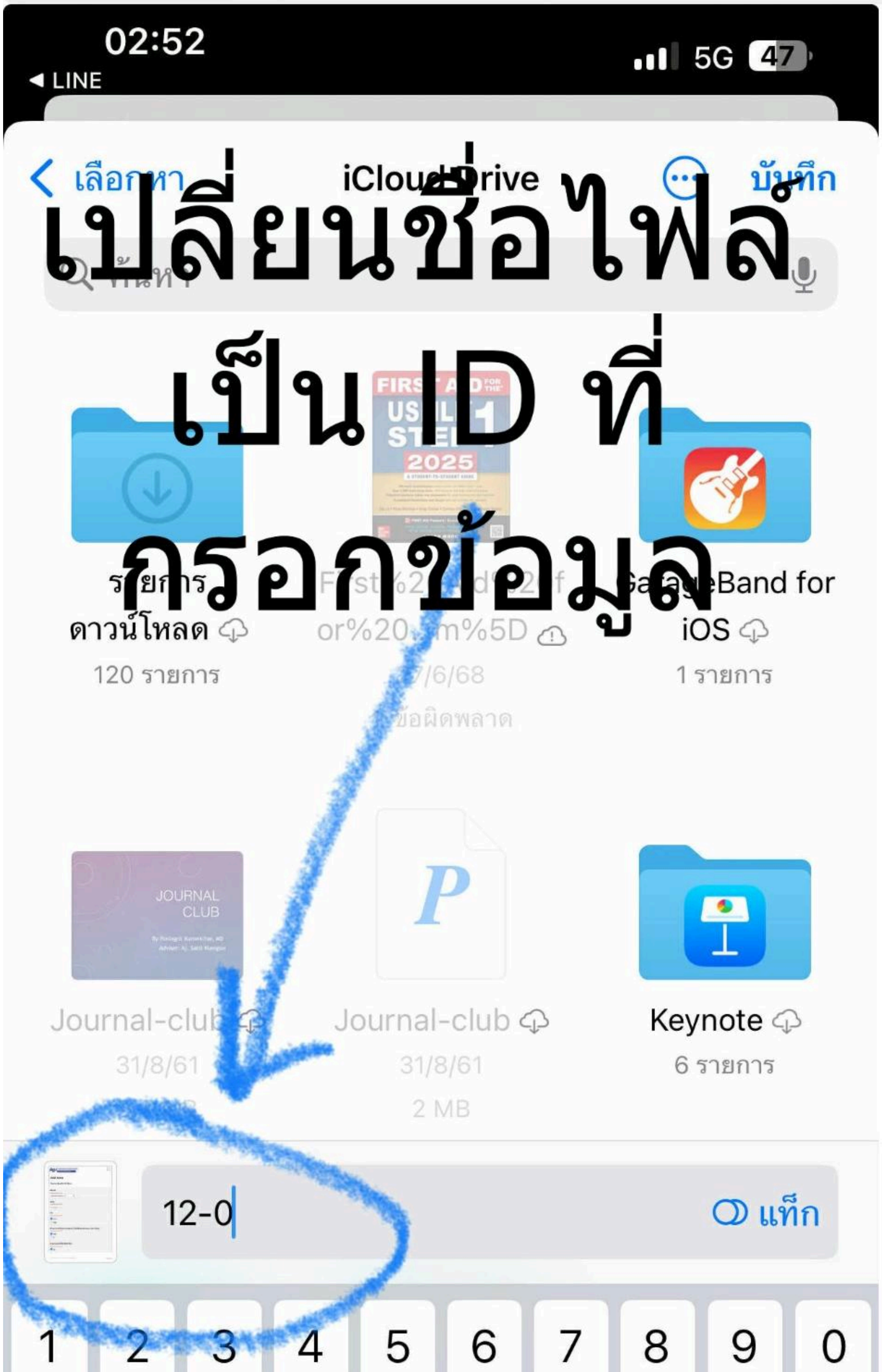
02:52
◀ LINE
5G 47
เลือกหา
iCloud Drive
บันทึก
เปลี่ยนชื่อไฟล์
เป็น ID ที่
กรอกข้อมูล
ดาวน์โหลด
120 รายการ
GarageBand for
iOS
1 รายการ
JOURNAL
CLUB
Journal-club
31/8/61
Journal-club
31/8/61
2 MB
Keynote
6 รายการ
12-0
แท็ก
1 2 3 4 5 6 7 8 9 0

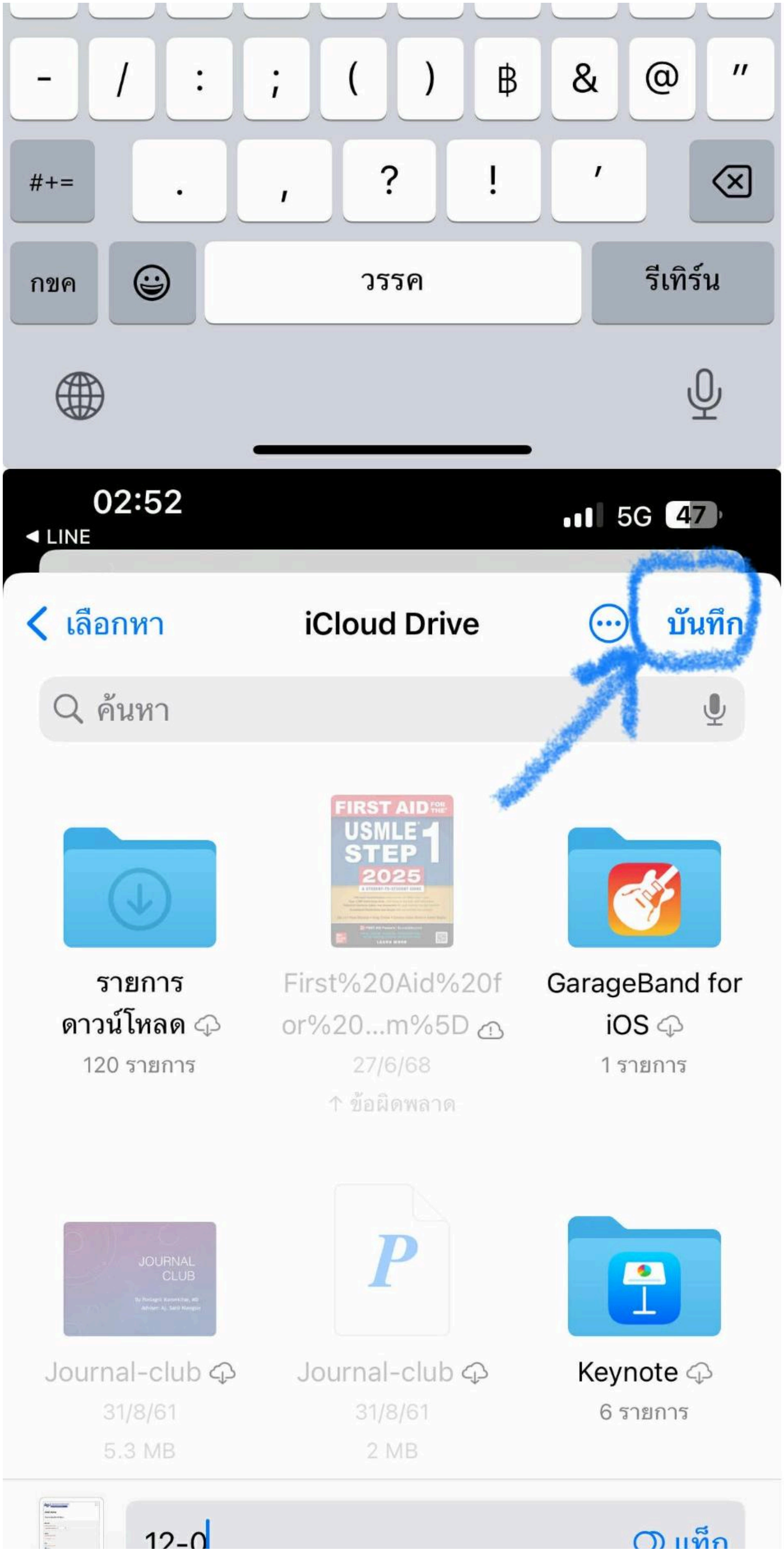

- / : ; ( ) ฿ & @ "
#+= . , ? ! '
กขค
วรรค
รีเทิร์น
02:52
◀ LINE
5G 47
เลือกหา
iCloud Drive
บันทึก
ค้นหา
FIRST AID FOR THE USMLE STEP 1 2025
รายการ ดาวน์โหลด
120 รายการ
First%20Aid%20f or%20...m%5D
27/6/68
ข้อผิดพลาด
GarageBand for iOS
1 รายการ
JOURNAL CLUB
Journal-club
31/8/61
5.3 MB
Journal-club
31/8/61
2 MB
Keynote
6 รายการ
12-0
แท็ก

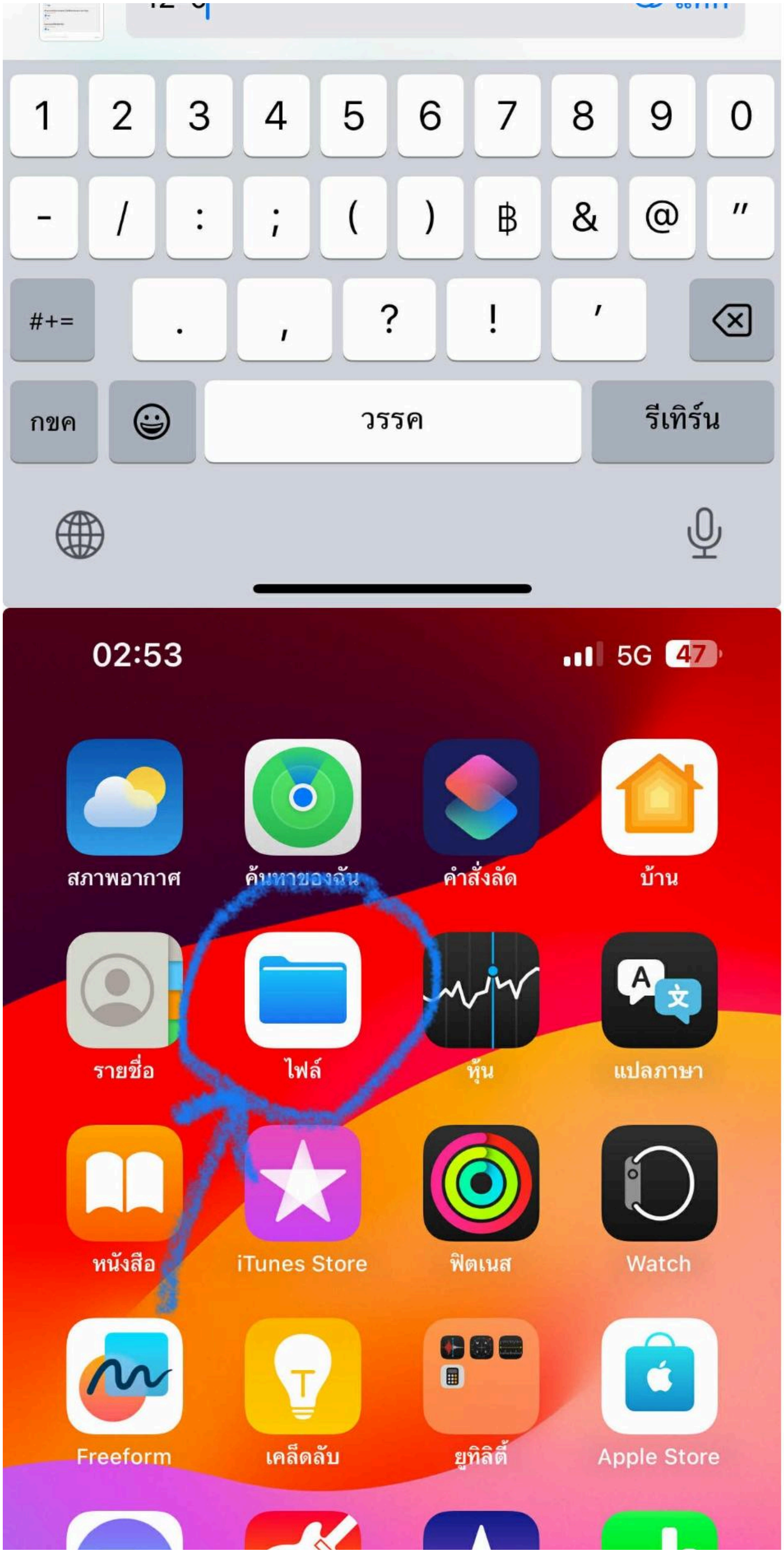
1 2 3 4 5 6 7 8 9 0
- / : ; ( ) ฿ & @ "
#+= . , ? ! '
กขค
วรรค
รีเทิร์น
02:53
5G 47
สภาพอากาศ
ค้นหาของฉัน
คำสั่งลัด
บ้าน
รายชื่อ
ไฟล์
หุ้น
แปลภาษา
หนังสือ
iTunes Store
ฟิตเนส
Watch
Freeform
เคล็ดลับ
ยูทิลิตี้
Apple Store

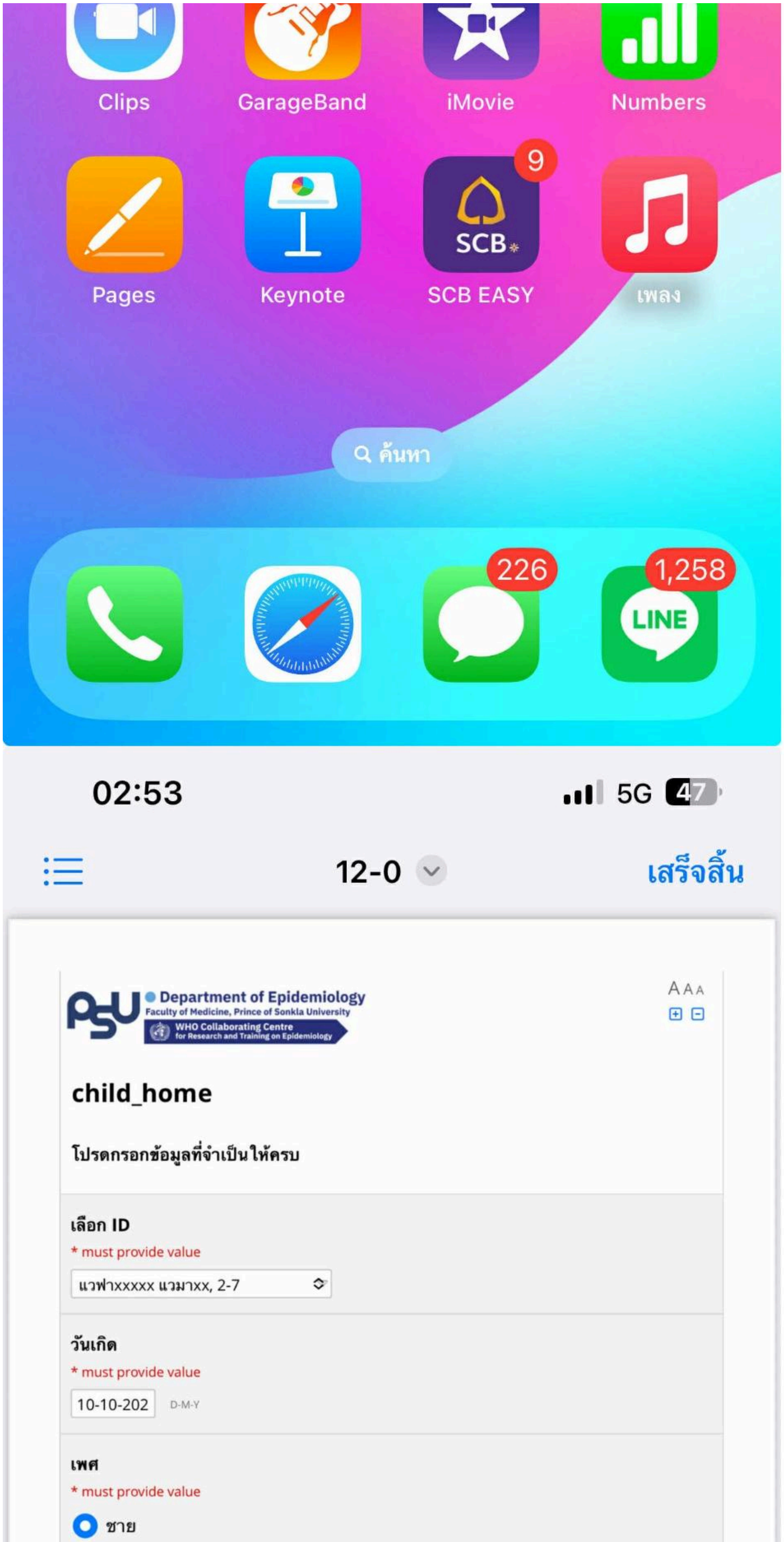
Clips
GarageBand
iMovie
Numbers
9
SCB
Pages
Keynote
SCB EASY
เพลง
ค้นหา
226
1,258
LINE
02:53
5G 47
12-0
เสร็จสิ้น
Department of Epidemiology
Faculty of Medicine, Prince of Sonkla University
WHO Collaborating Centre for Research and Training on Epidemiology
child_home
โปรดกรอกข้อมูลที่จำเป็นให้ครบ
เลือก ID
* must provide value
แวฬาxxxxx แวมาxx, 2-7
วันเกิด
* must provide value
10-10-202
D-M-Y
เพศ
* must provide value
ชาย

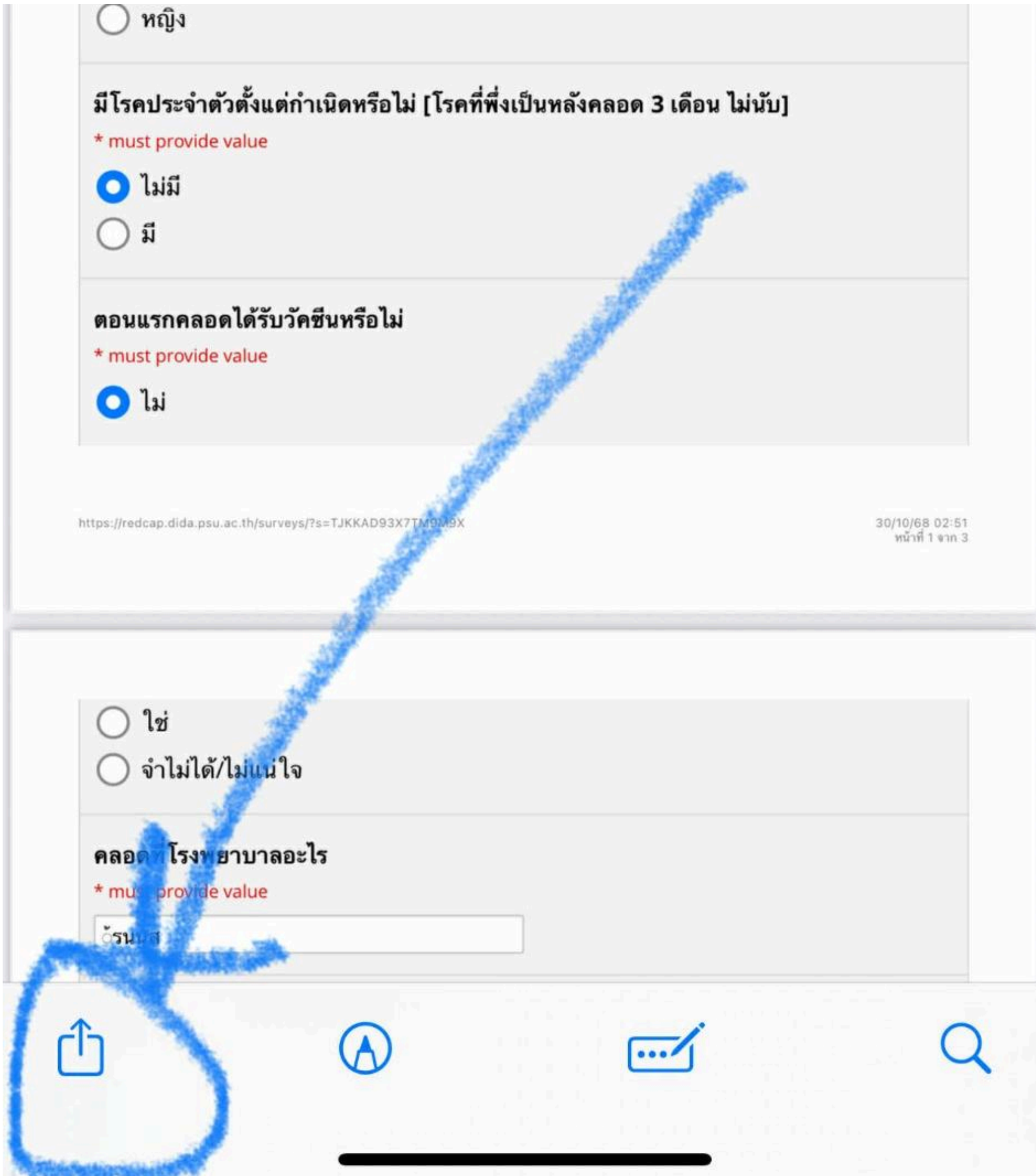
หญิง
มีโรคประจำตัวตั้งแต่กำเนิดหรือไม่ [โรคที่พึ่งเป็นหลังคลอด 3 เดือน ไม่นับ]
* must provide value
ไม่มี
มี
ตอนแรกคลอดได้รับวัคซีนหรือไม่
* must provide value
ไม่
30/10/68 02:51
หน้าที่ 1 จาก 3
ใช่
จำไม่ได้/ไม่แน่ใจ

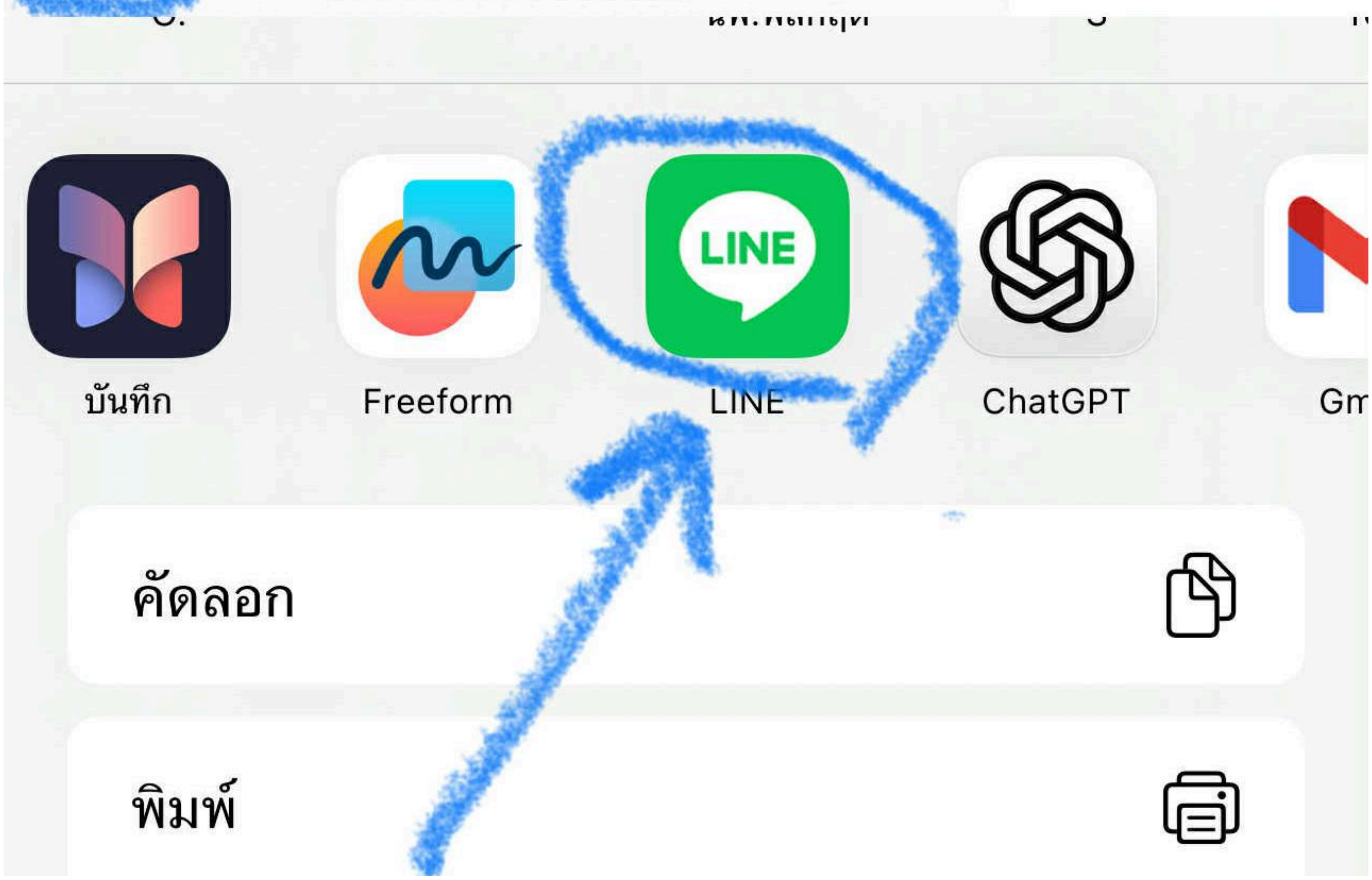
บันทึก
Freeform
LINE
ChatGPT
คัดลอก
พิมพ์

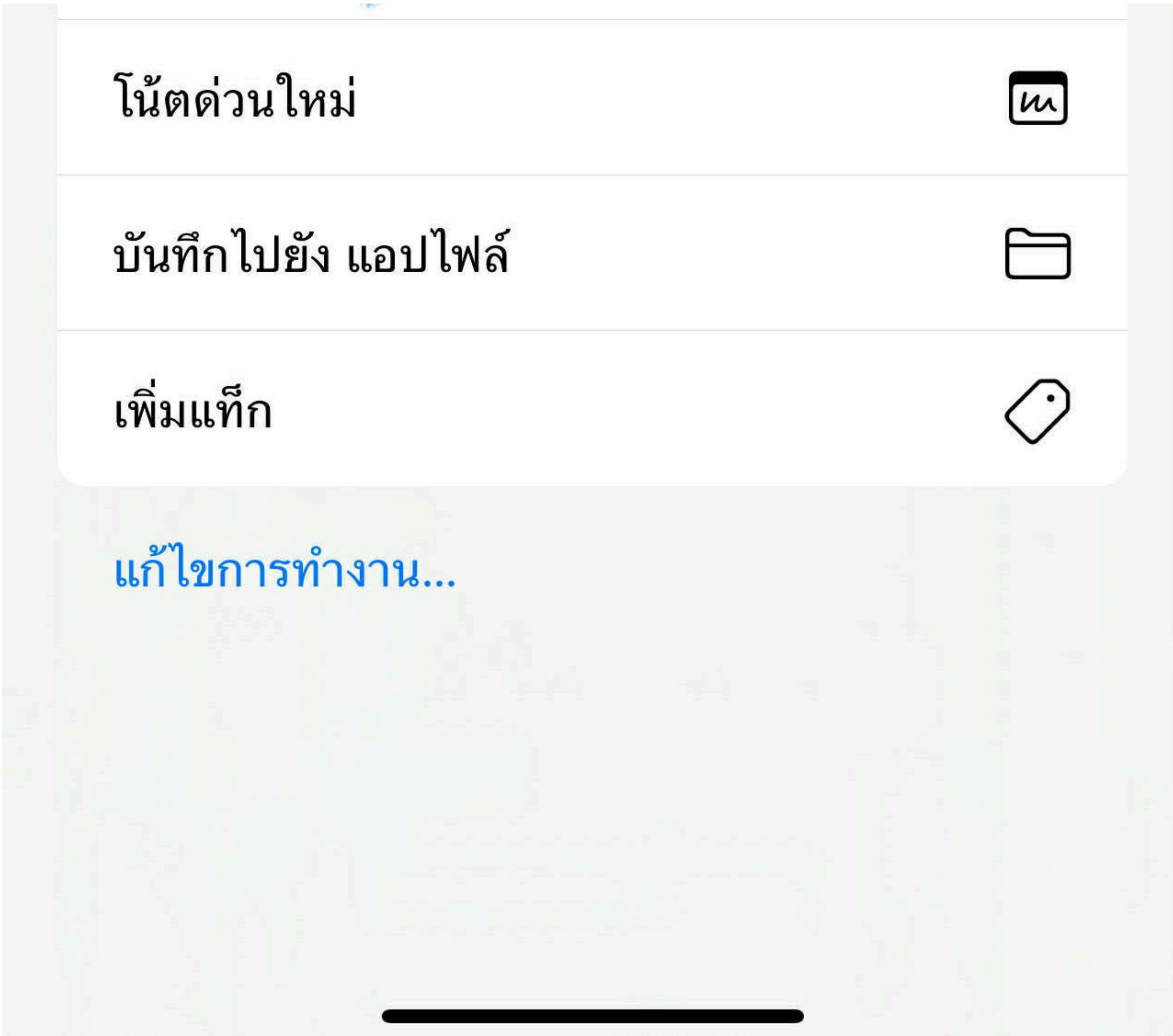

- สำหรับ Android

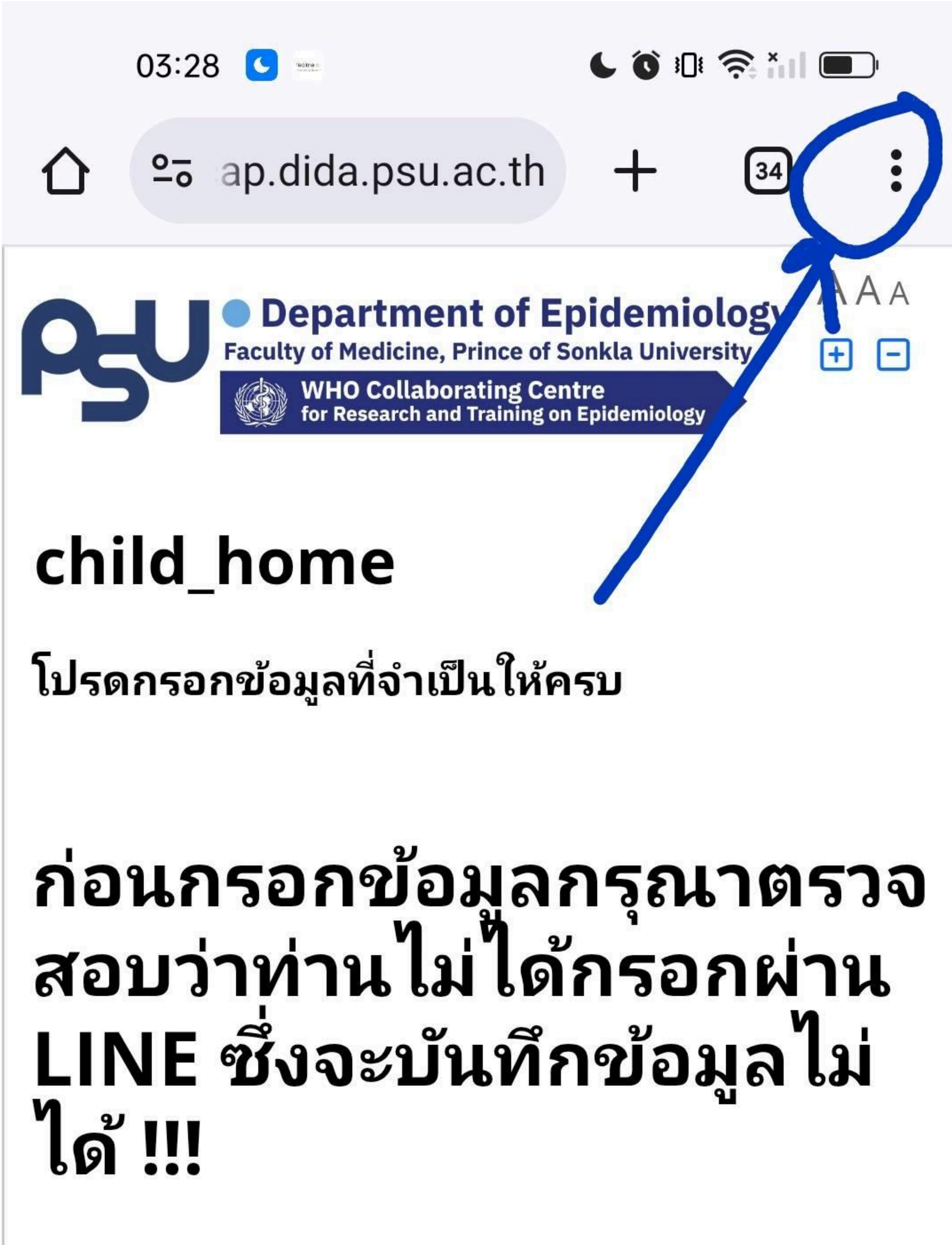
03:28
ap.dida.psu.ac.th
34
A A A
Department of Epidemiology
Faculty of Medicine, Prince of Sonkla University
WHO Collaborating Centre
for Research and Training on Epidemiology
child_home
โปรดกรอกข้อมูลที่จำเป็นให้ครบ
ก่อนกรอกข้อมูลกรุณาตรวจสอบว่าท่านไม่ได้กรอกผ่าน LINE ซึ่งจะบันทึกข้อมูลไม่ได้ !!!

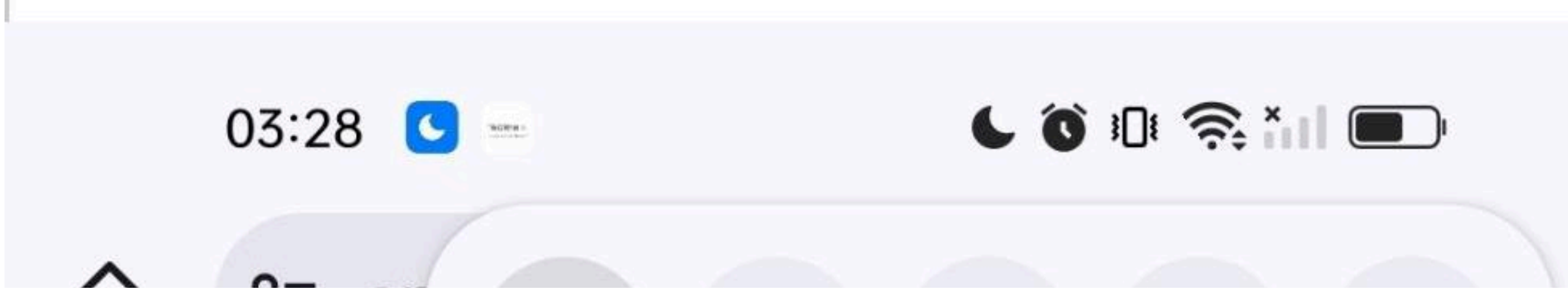
03:28

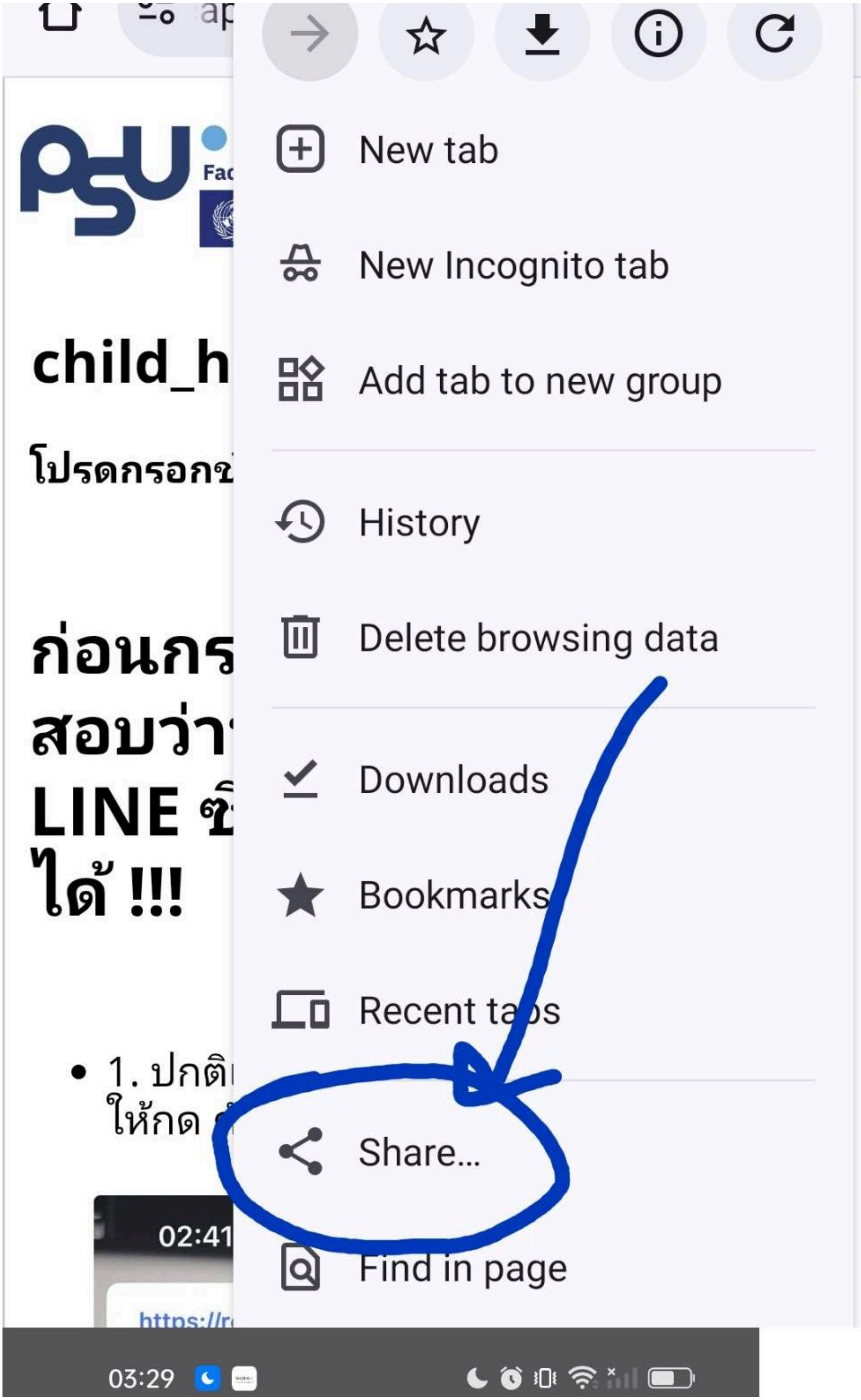
New tab
New Incognito tab
Add tab to new group
History
Delete browsing data
Downloads
Bookmarks
Recent tabs
Share...
Find in page
child_h
โปรดกรอกข
ก่อนกร
สอบว่า
LINE ซ
ได้ !!!
1. ปกติ
ให้กด
02:41
03:29

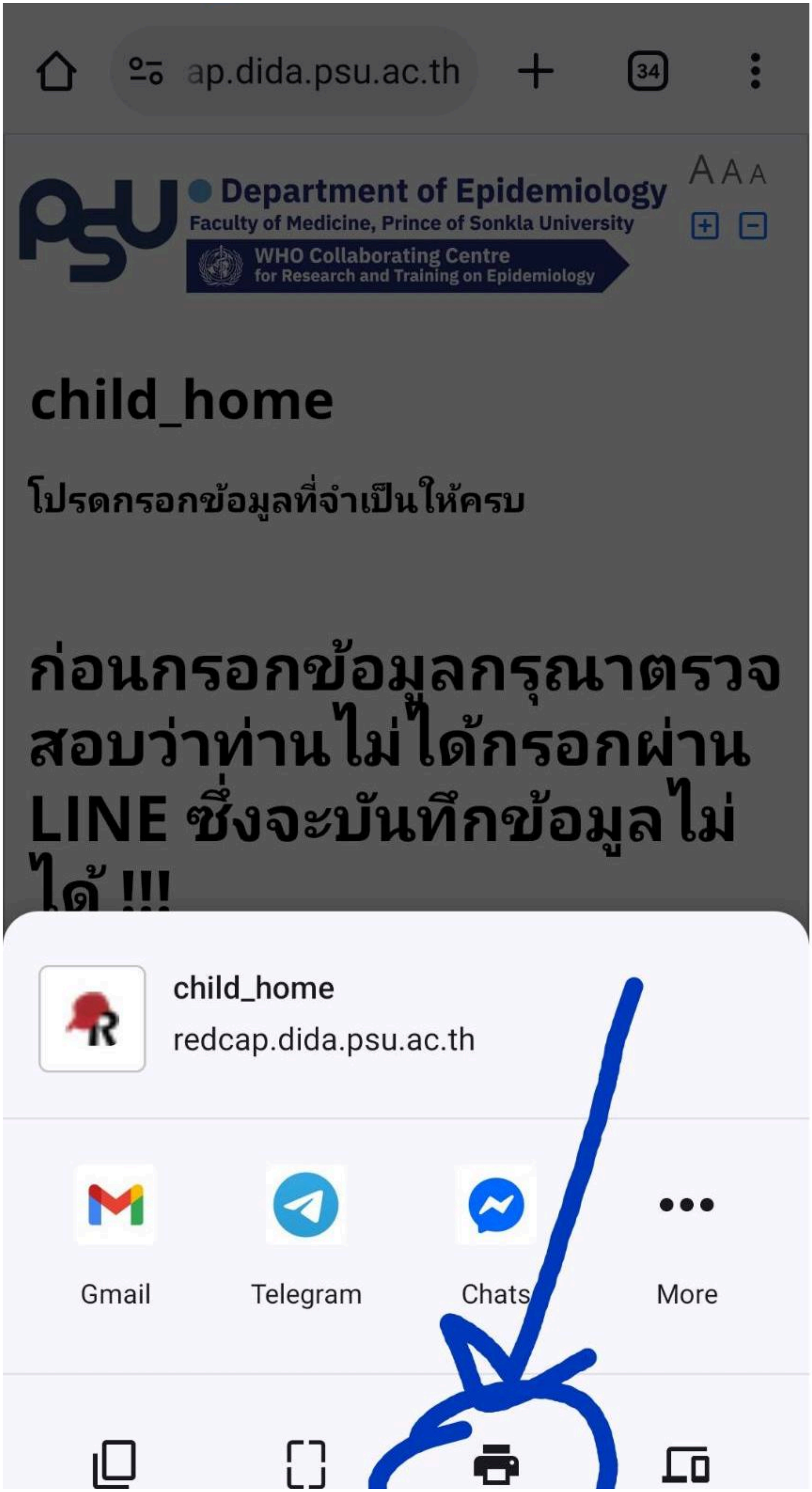
ap.dida.psu.ac.th
34
AAA
Department of Epidemiology
Faculty of Medicine, Prince of Sonkla University
WHO Collaborating Centre
for Research and Training on Epidemiology
child_home
โปรดกรอกข้อมูลที่จำเป็นให้ครบ
ก่อนกรอกข้อมูลกรุณาตรวจสอบว่าท่านไม่ได้กรอกผ่าน LINE ซึ่งจะบันทึกข้อมูลไม่ได้ !!!
child_home
redcap.dida.psu.ac.th
Gmail
Telegram
Chats
More

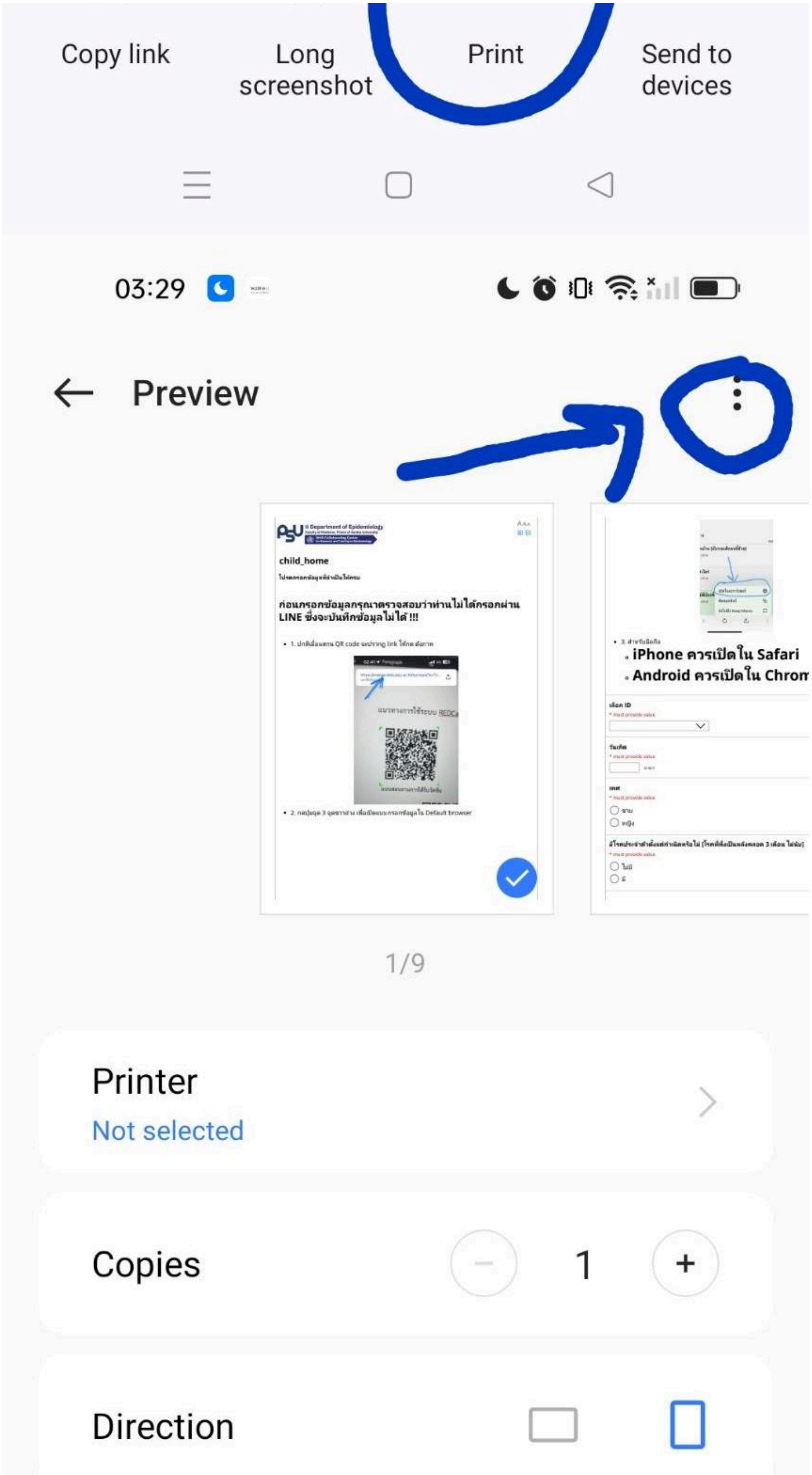
Copy link
Long screenshot
Print
Send to devices
03:29
Preview
child_home
ก่อนกรอกข้อมูลกรุณาตรวจสอบว่าท่านไม่ได้กรอกผ่าน LINE ซึ่งจะบันทึกข้อมูลไม่ได้ !!!
iPhone ควรเปิดใน Safari
Android ควรเปิดใน Chrom
1/9
Printer
Not selected
Copies
1
Direction

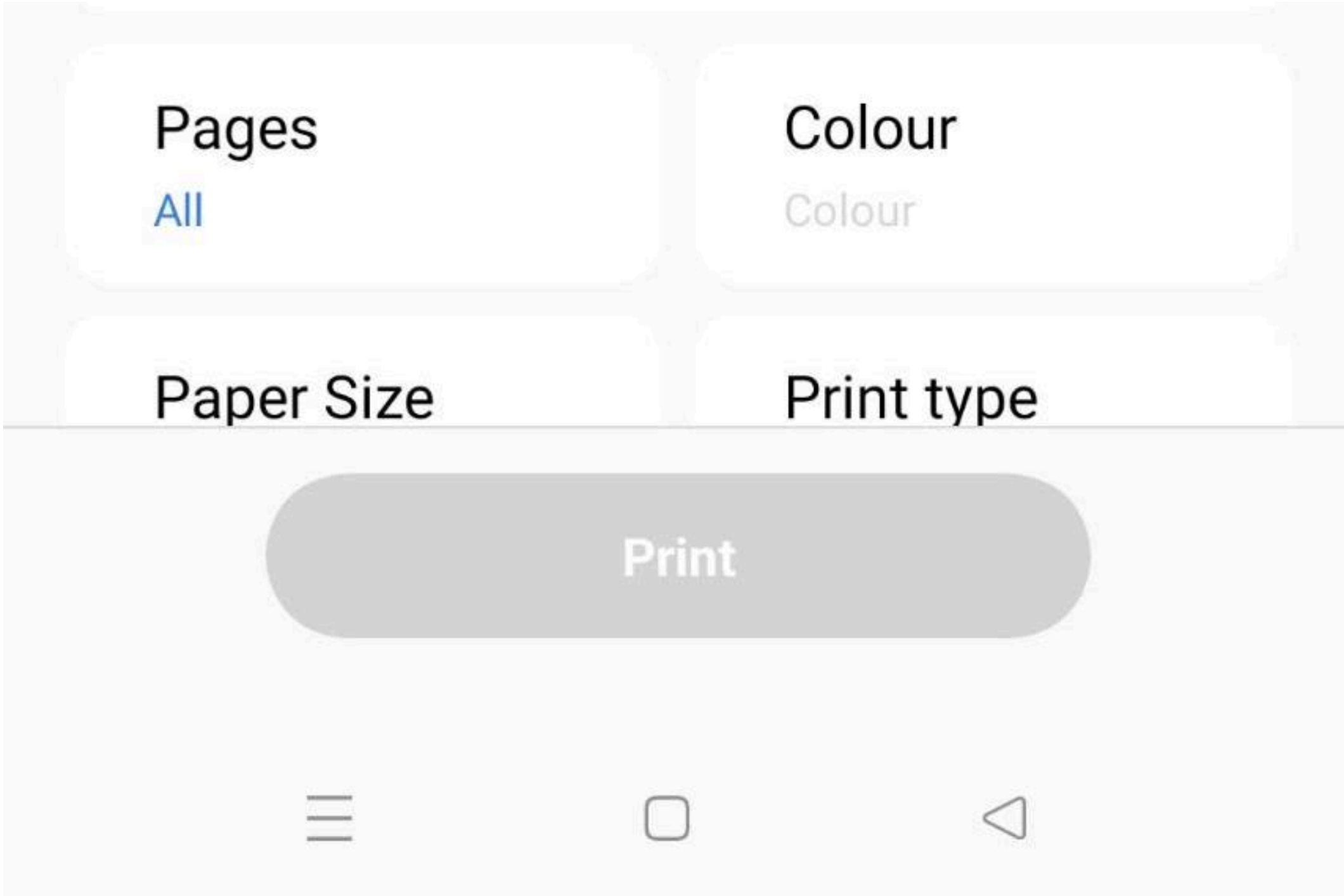
Pages
All
Colour
Colour
Paper Size
Print type
Print

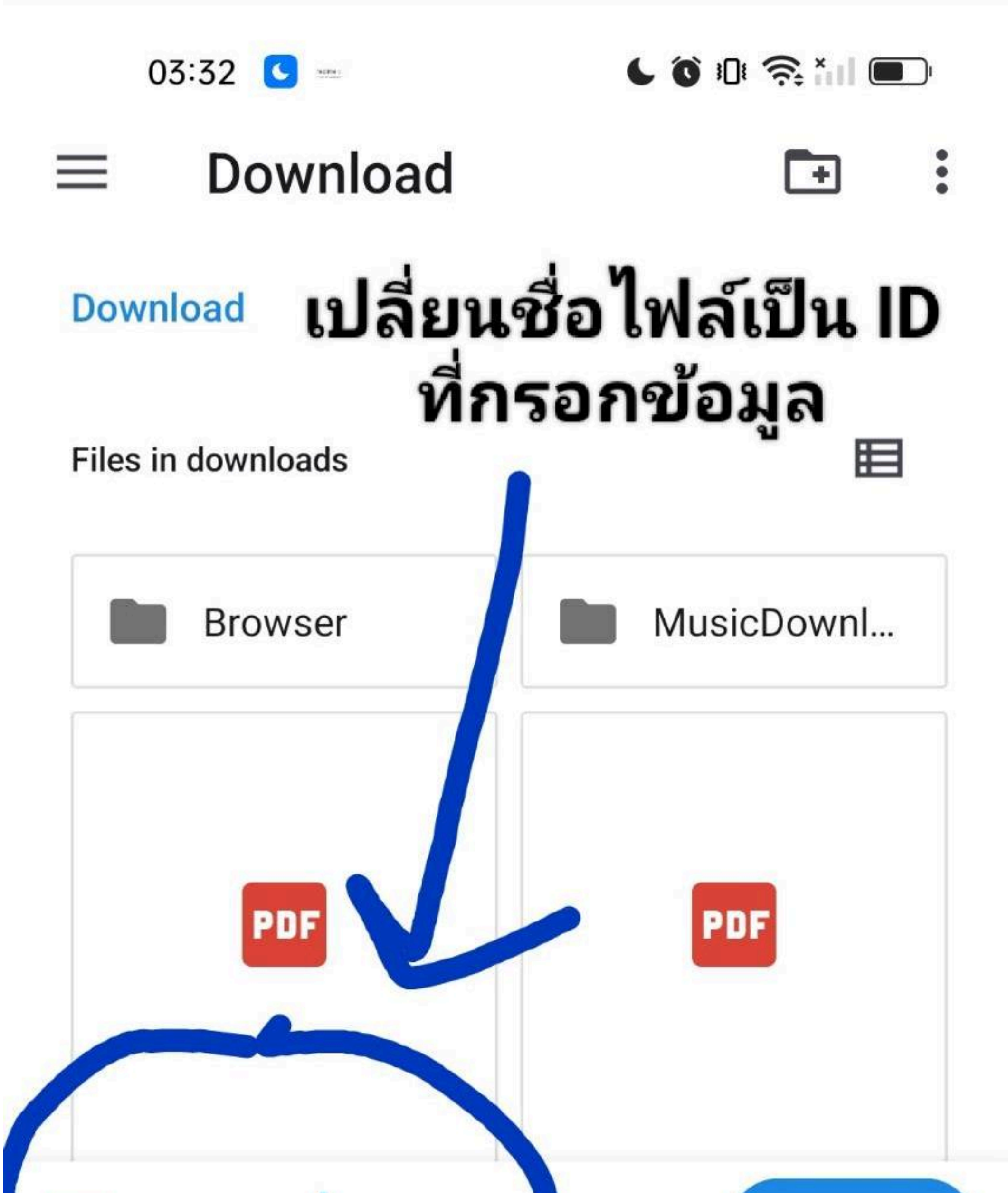
03:32
Download
Download
เปลี่ยนชื่อไฟล์เป็น ID
ที่กรอกข้อมูล
Files in downloads
Browser
MusicDownl...
PDF
PDF

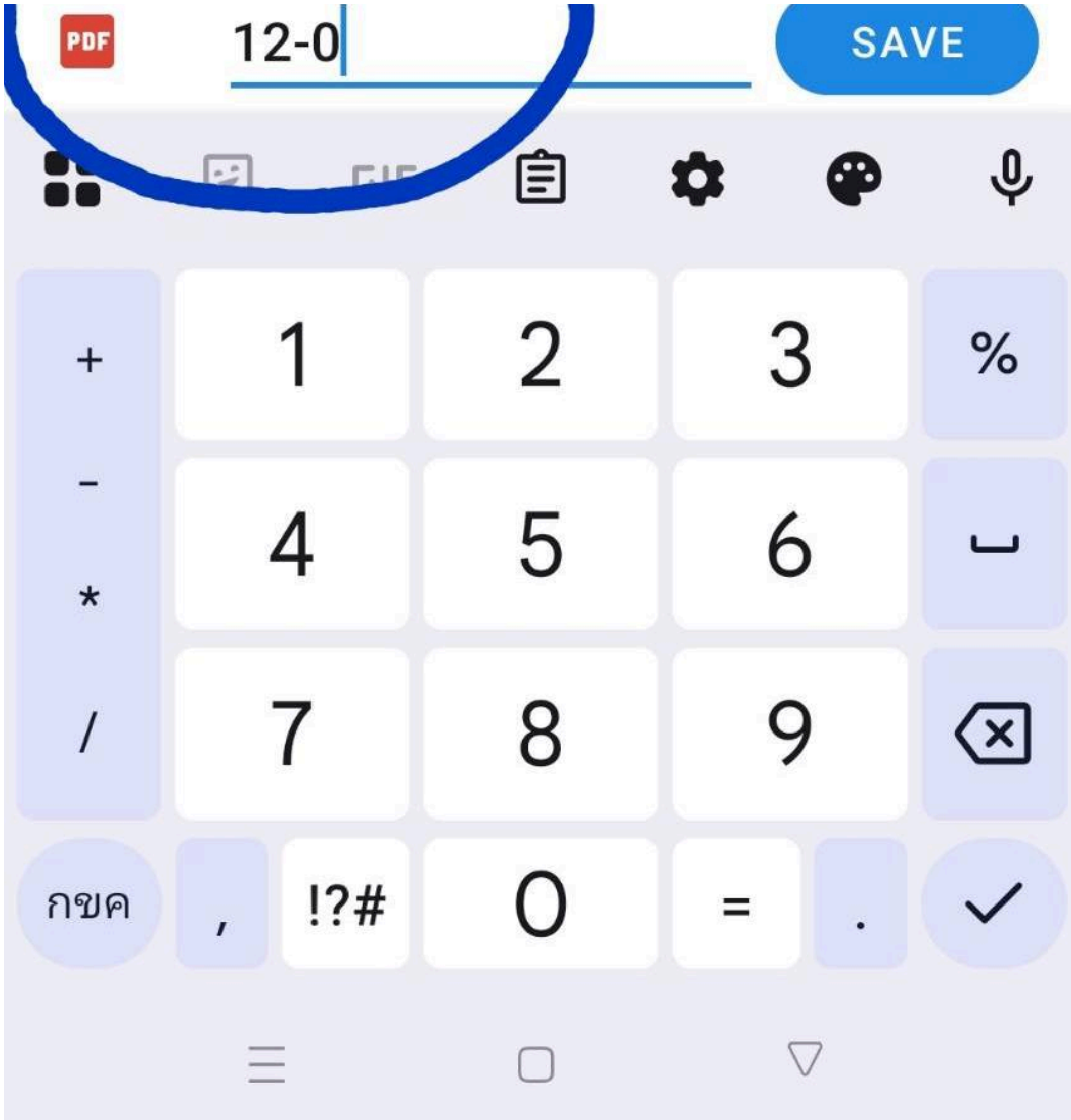
12-0
SAVE
+
-
*
/
1
2
3
%
4
5
6
7
8
9
กขค
,
!?#
0
=
.

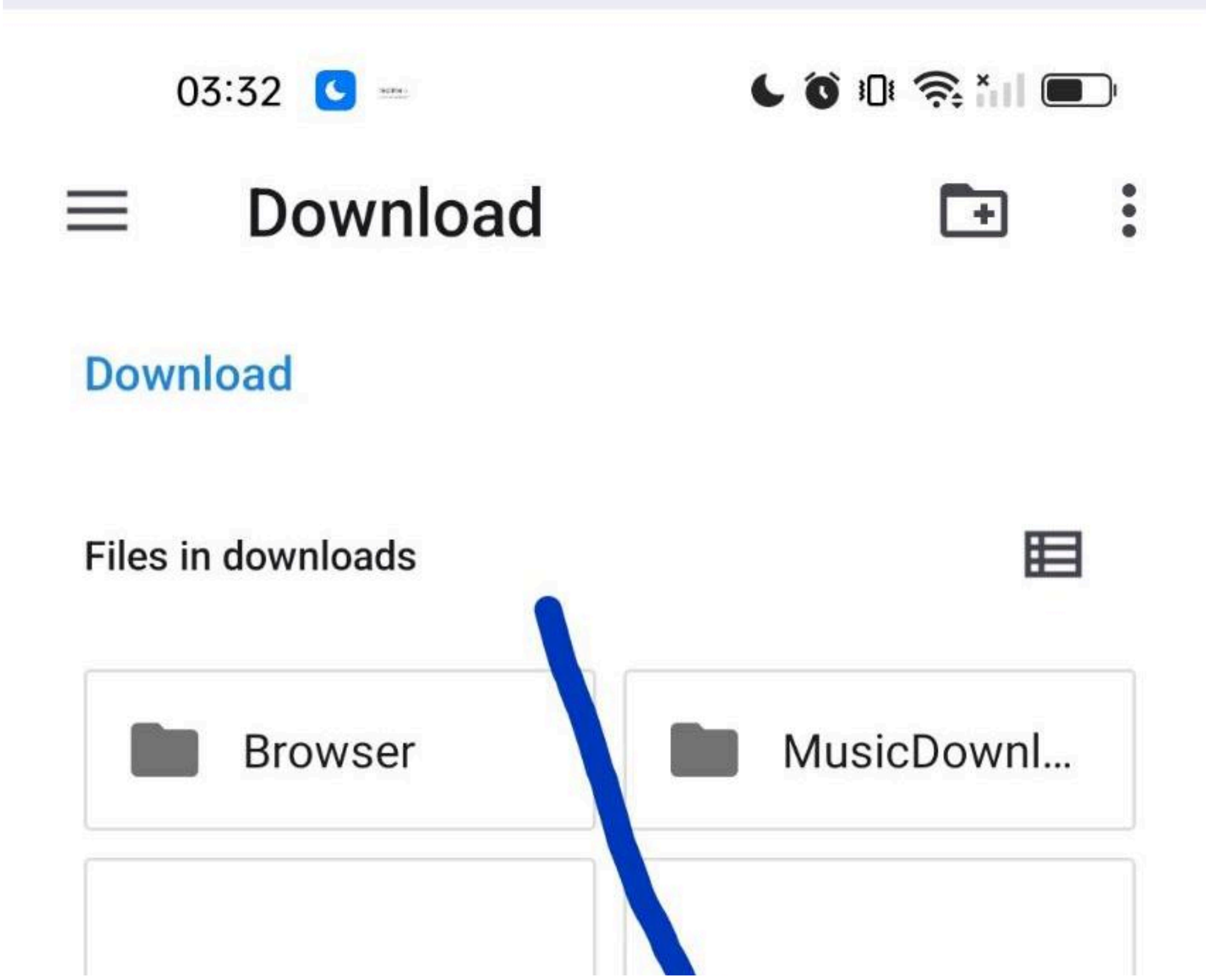
03:32
Download
Download
Files in downloads
Browser
MusicDownl...

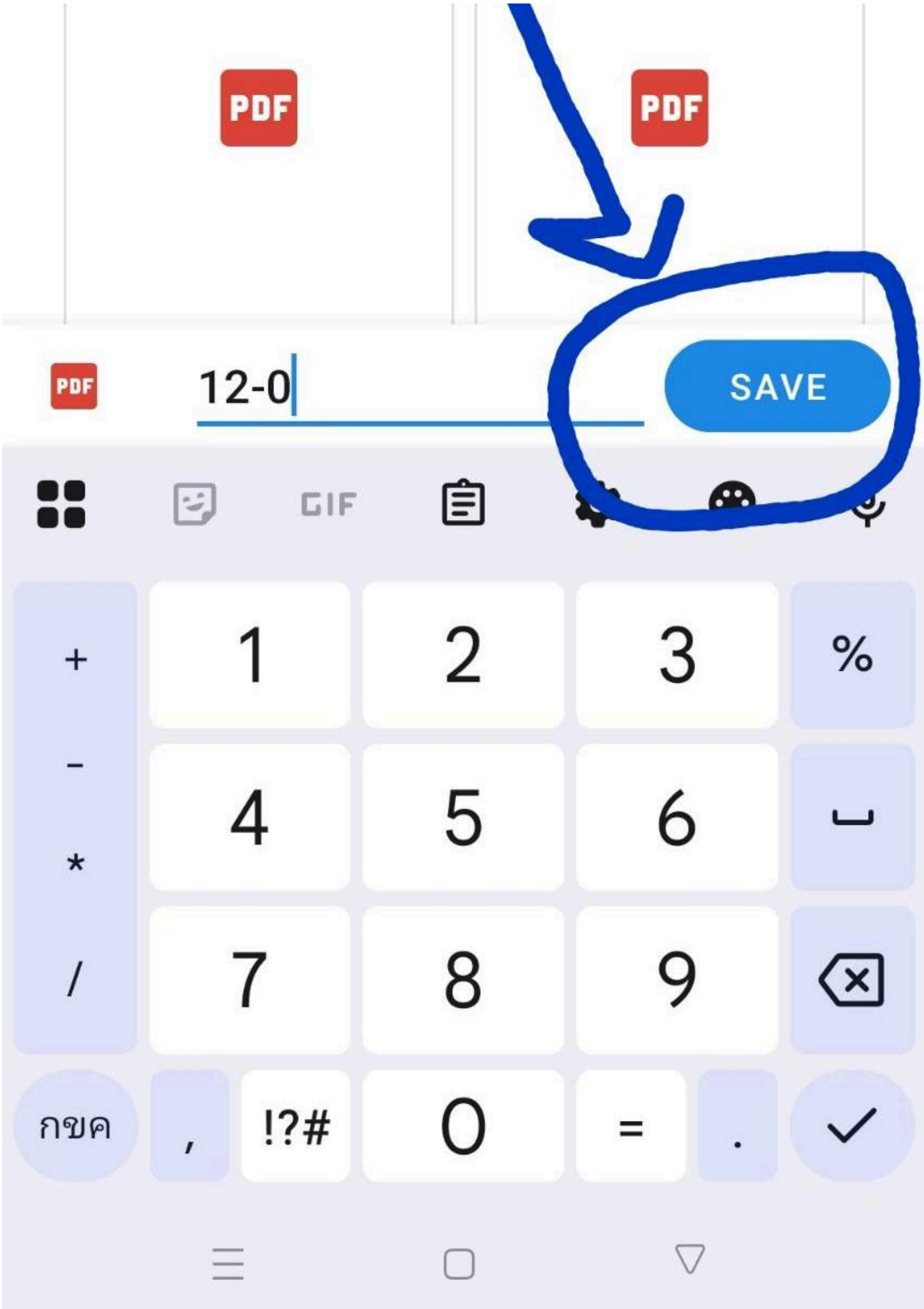
PDF
PDF
PDF
12-0
SAVE
GIF
+
1
2
3
%
-
4
5
6
*
/
7
8
9
กขค
,
!?#
0
=
.

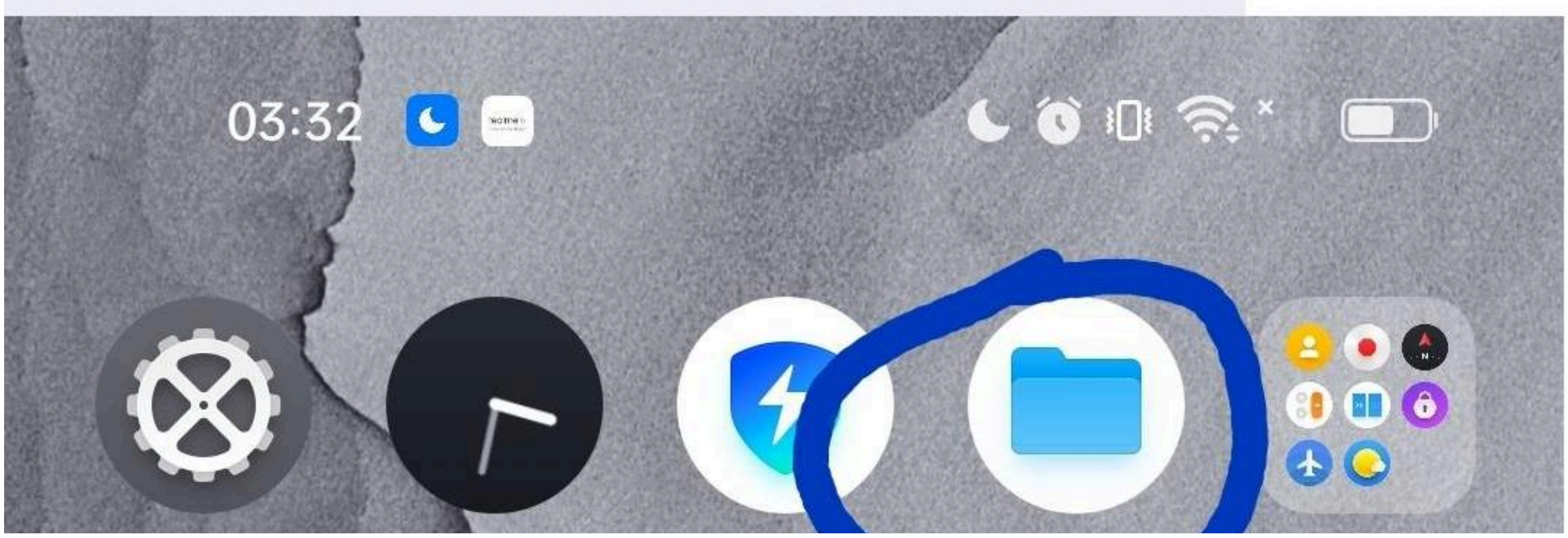
03:32

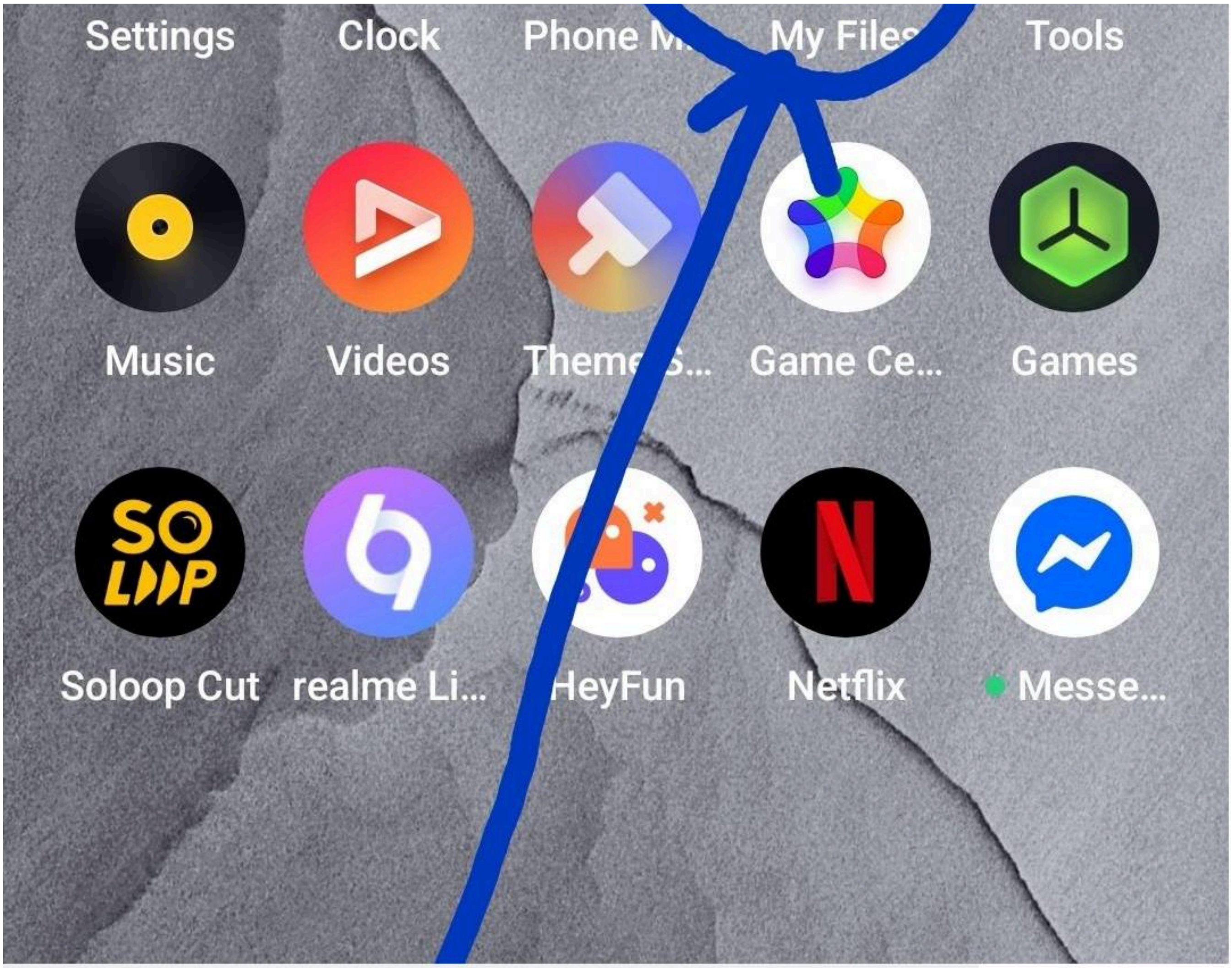
Settings
Clock
Phone M...
My Files
Tools
Music
Videos
Theme S...
Game Ce...
Games
Soloop Cut
realme Li...
HeyFun
Netflix
Messe...

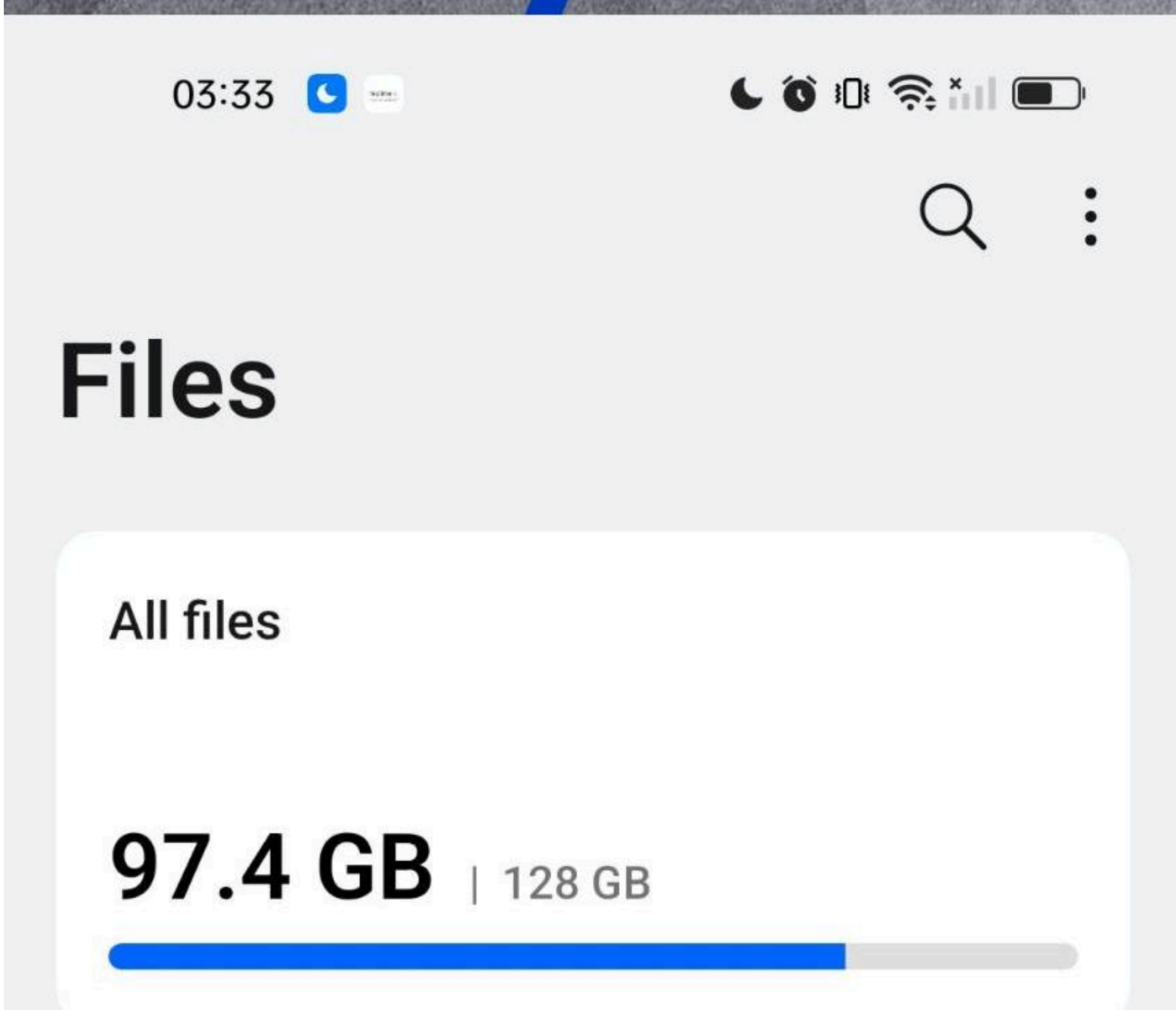
03:33
Files
All files
97.4 GB | 128 GB

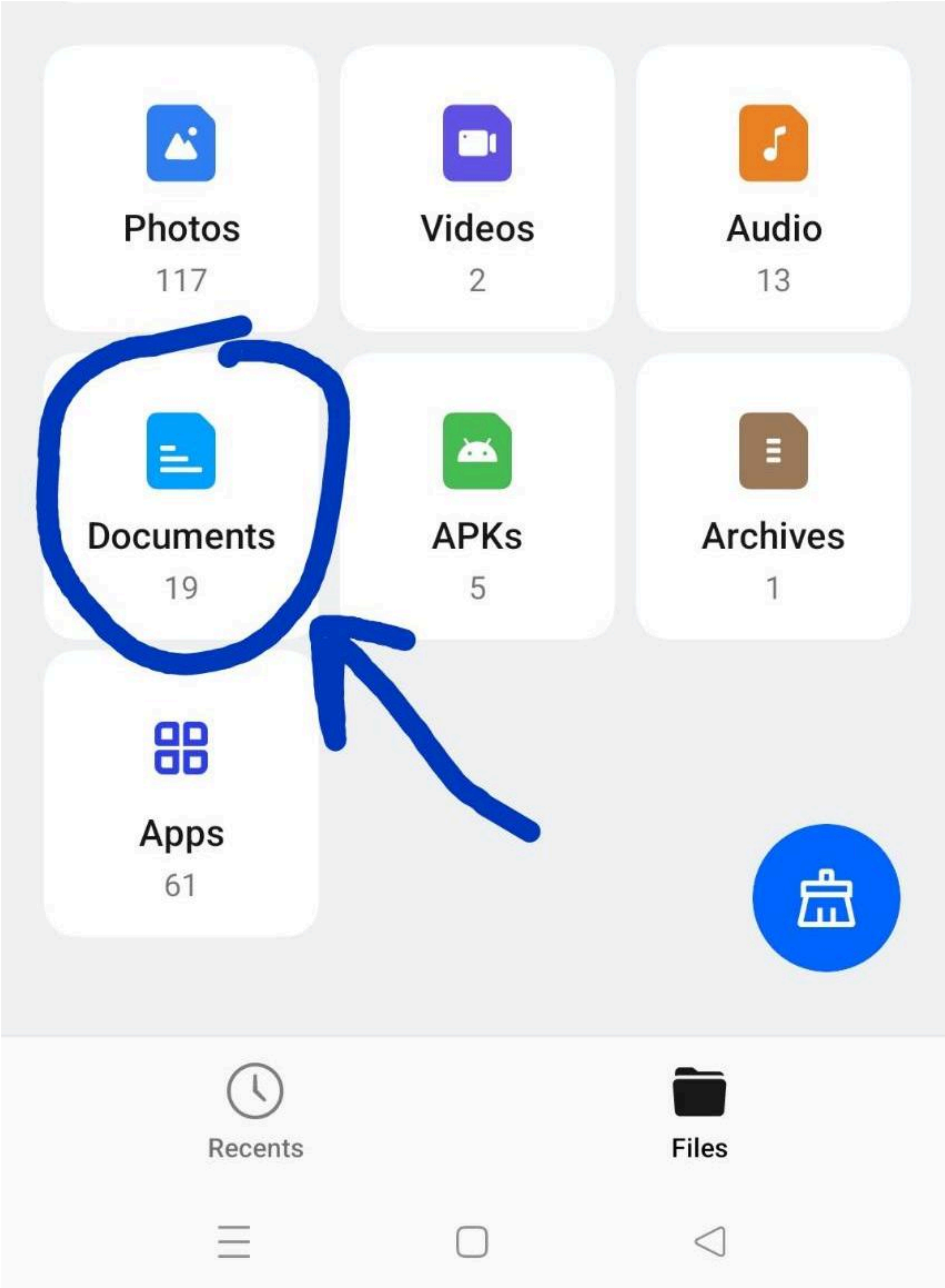
Photos
117
Videos
2
Audio
13
Documents
19
APKs
5
Archives
1
Apps
61
Recents
Files

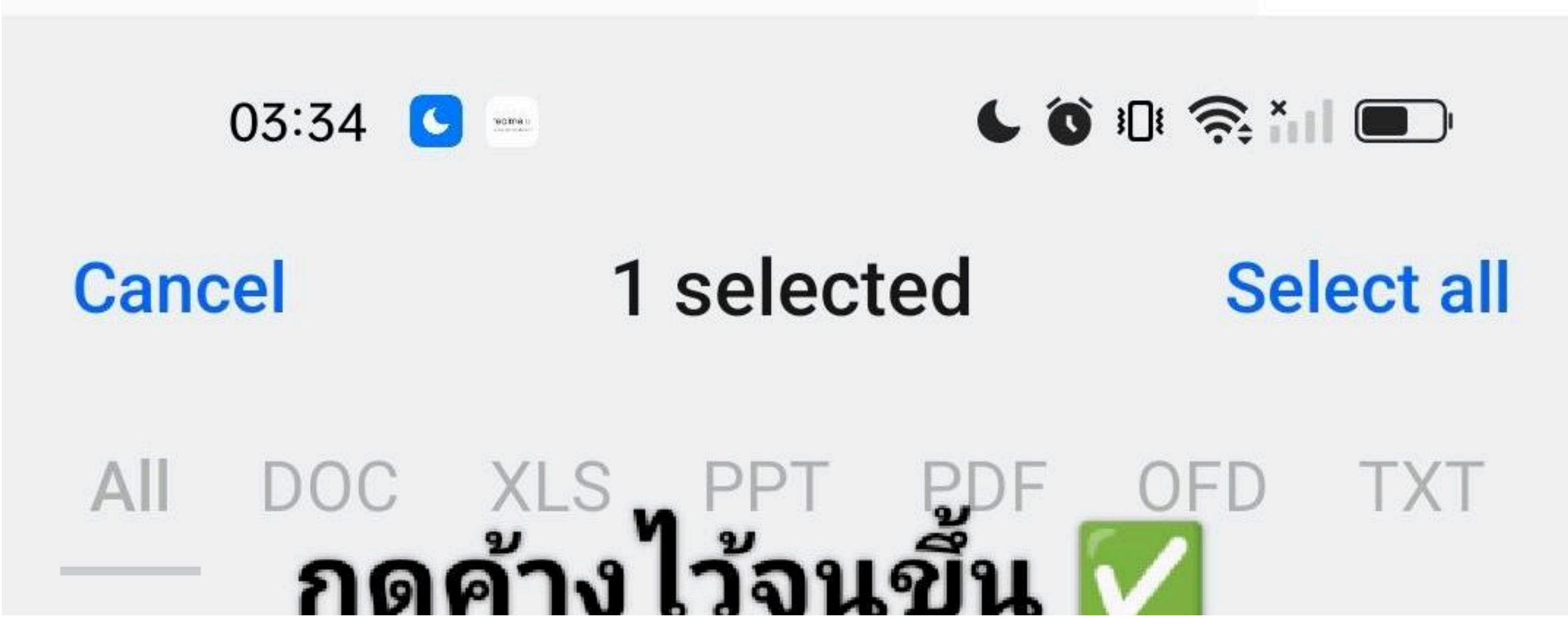
03:34
Cancel
1 selected
Select all
All
DOC
XLS
PPT
PDF
OFD
TXT
กดค้างไว้จนขึ้น ✅

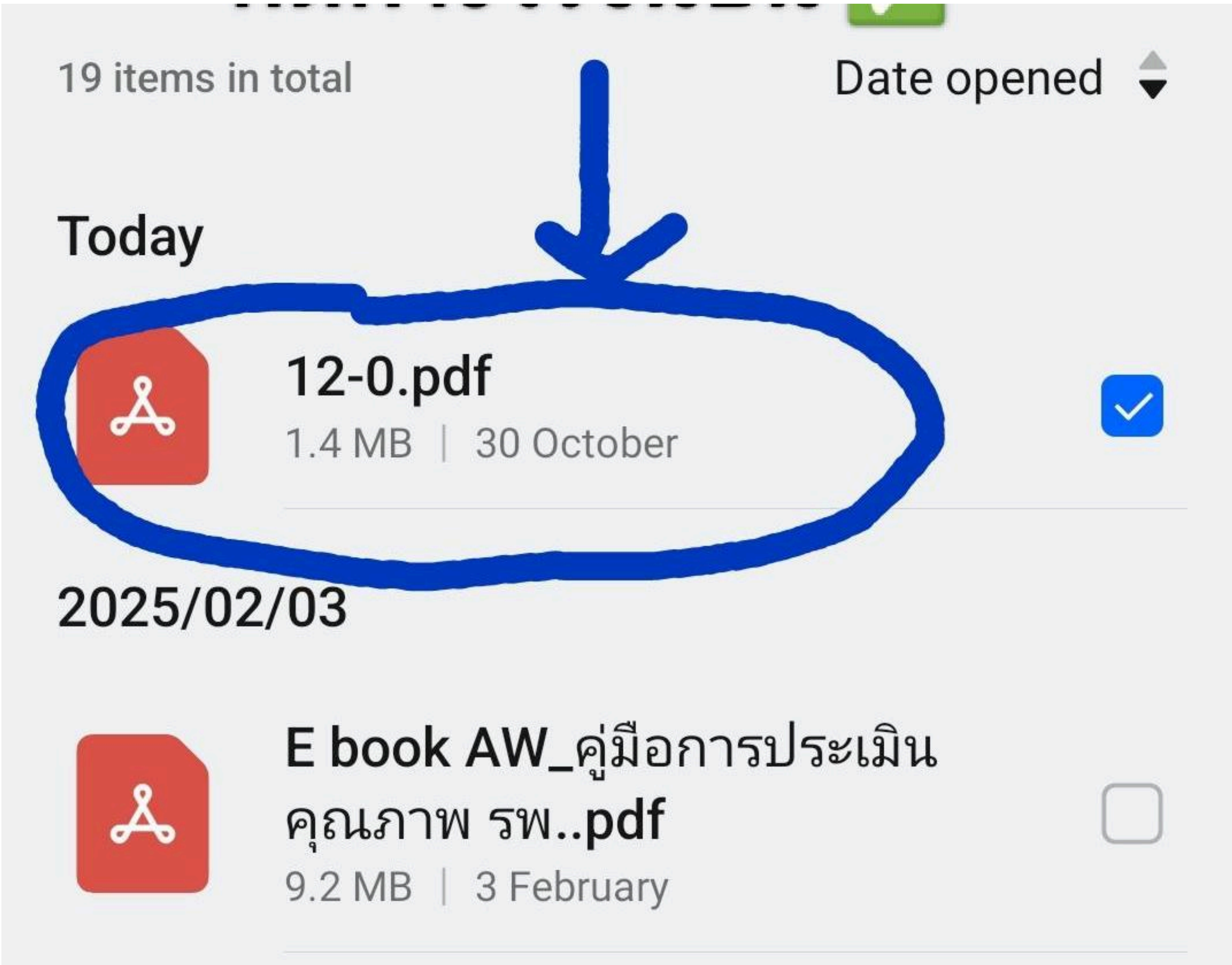
19 items in total
Date opened
Today
12-0.pdf
1.4 MB | 30 October
2025/02/03
E book AW_คู่มือการประเมินคุณภาพ รพ..pdf
9.2 MB | 3 February

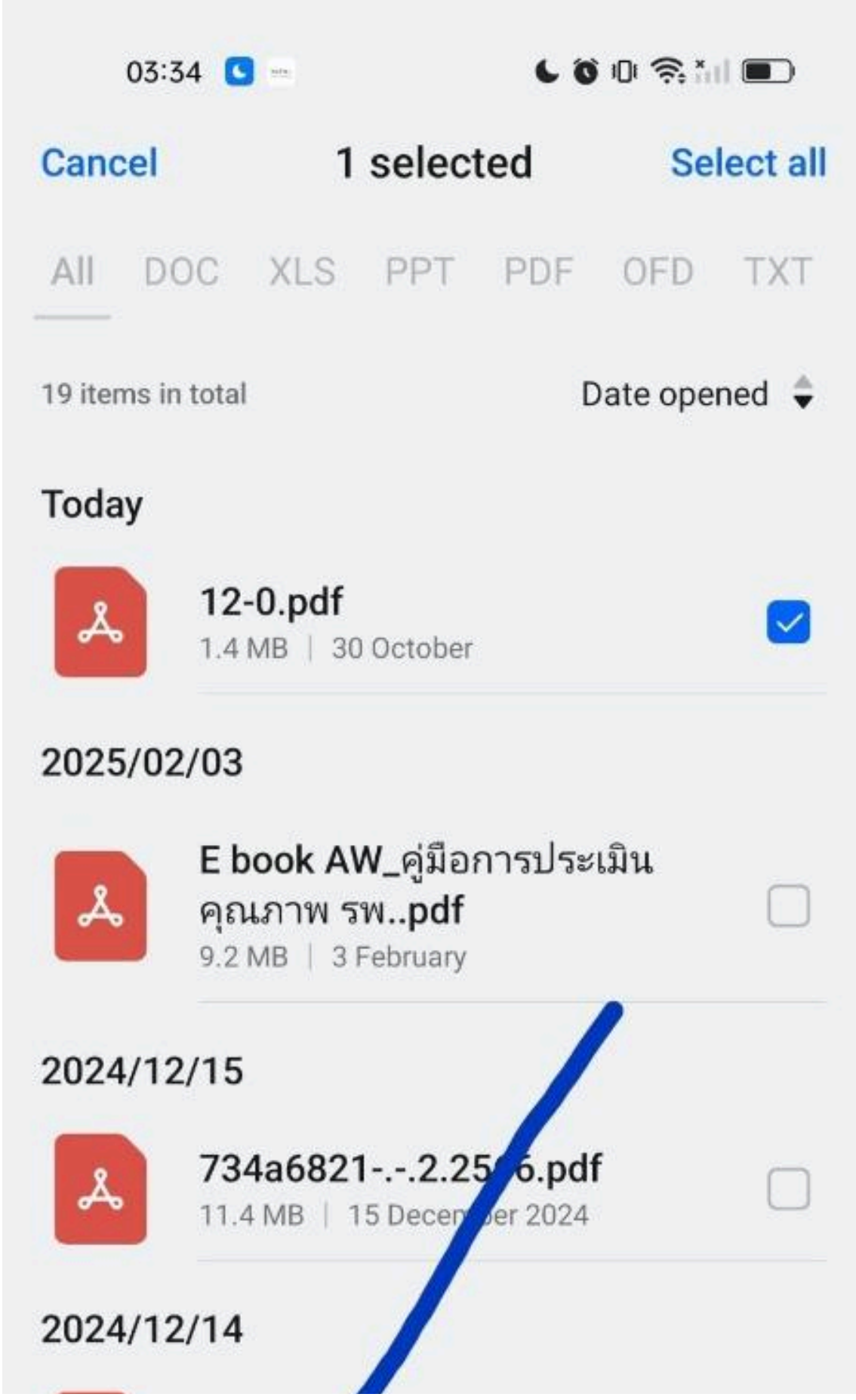
03:34
Cancel
1 selected
Select all
All DOC XLS PPT PDF OFD TXT
19 items in total
Date opened
Today
12-0.pdf
1.4 MB | 30 October
2025/02/03
E book AW_คู่มือการประเมินคุณภาพ รพ..pdf
9.2 MB | 3 February
2024/12/15
11.4 MB
2024/12/14

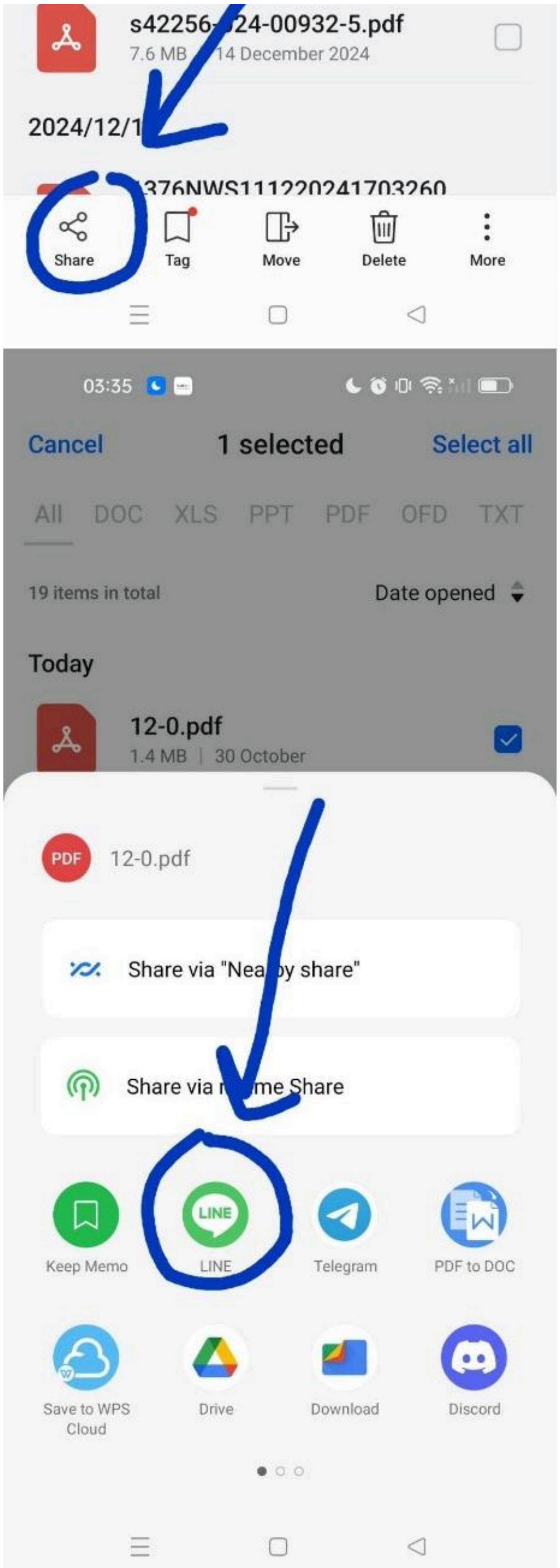

Submit

Powered by REDCap

DEPARTMENT OF EPIDEMIOLOGY
FACULTY OF MEDICINE
PRINCE OF SONGKLA UNIVERSITY

child_home

โปรดกรอกข้อมูลที่จำเป็นให้ครบ

ก่อนกรอกข้อมูลกรุณาตรวจสอบว่าท่านไม่ได้กรอกผ่าน LINE ซึ่งจะบันทึกข้อมูลไม่ได้ !!!

- 1. ปกติเมื่อแสกน QR code จะปรากฏ link ให้กด ดังภาพ

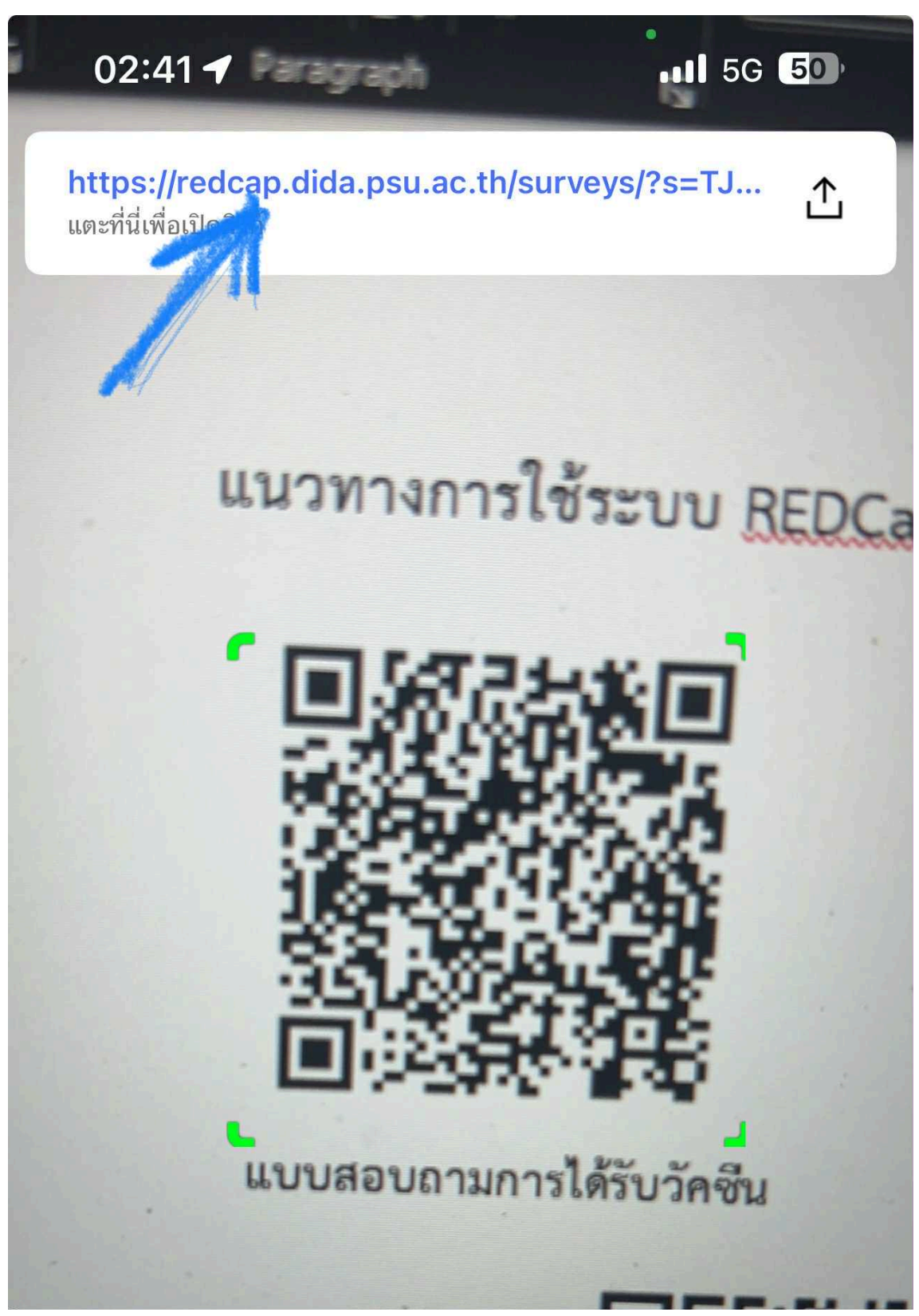

- 2. กดปุ่มจุด 3 จุดขวาล่าง เพื่อเปิดแบบกรอกข้อมูลใน Default browser

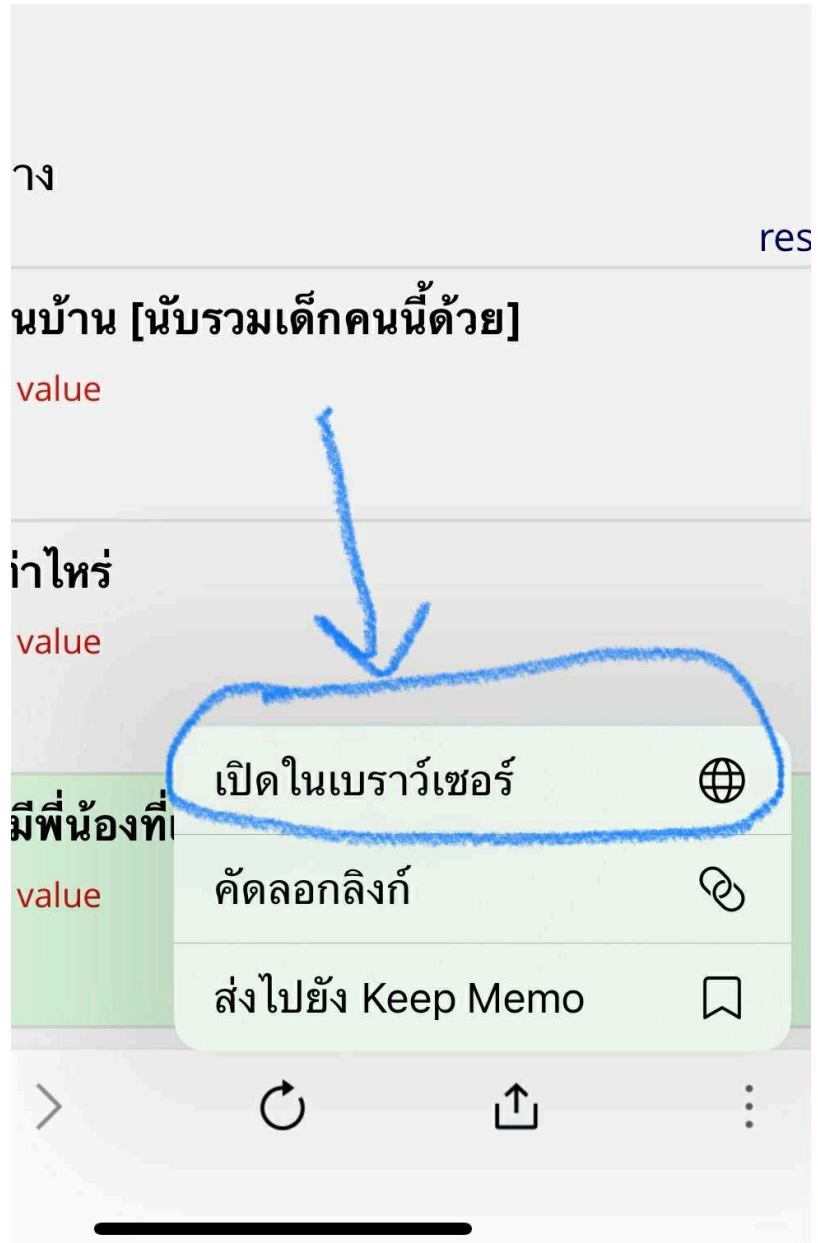

- 3. สำหรับมือถือ
  - # iPhone ควรเปิดใน Safari
  - # Android ควรเปิดใน Chrome
  - **หากไม่ใช่ให้ copy url ไปเปิดใน browser ที่ถูกต้อง**

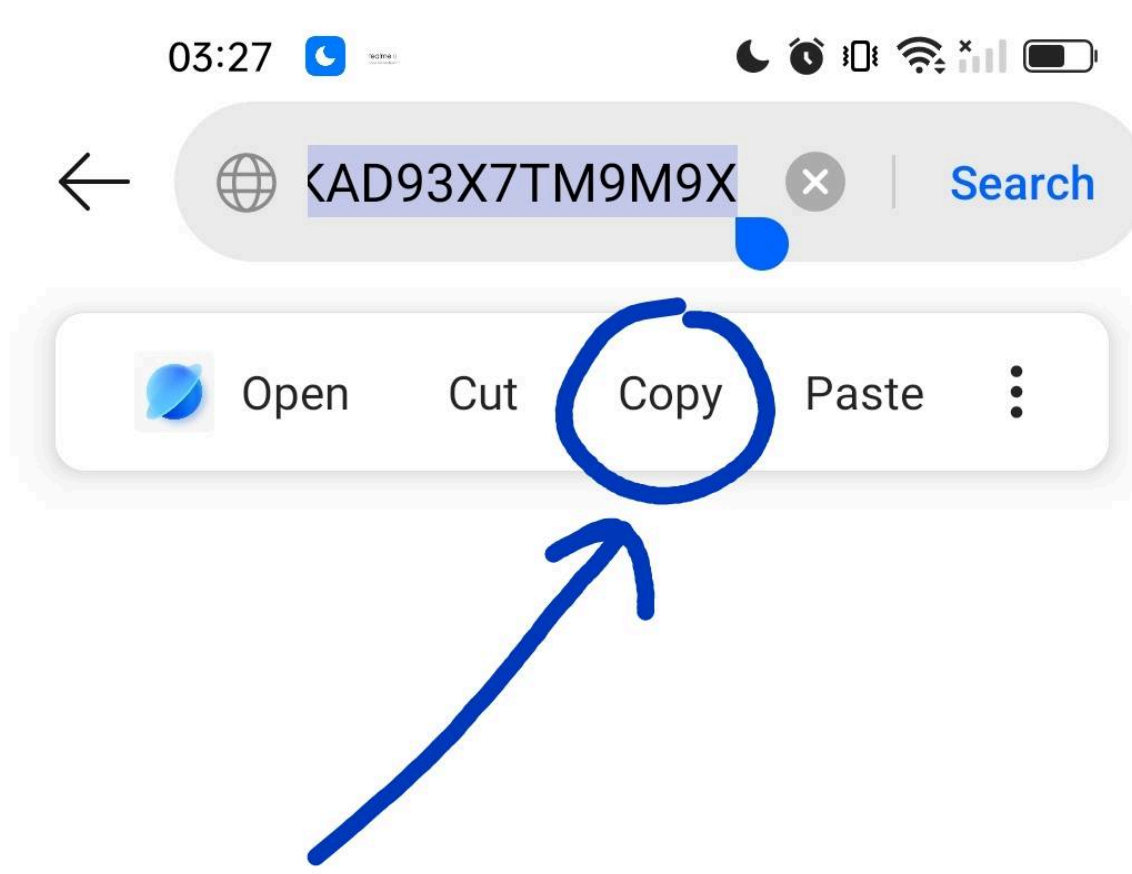

เลือก ID

* must provide value

วันเกิด

* must provide value

[ ] D-M-Y

เพศ

* must provide value

- ชาย
- หญิง

มีโรคประจำตัวตั้งแต่กำเนิดหรือไม่ [โรคที่พึ่งเป็นหลังคลอด 3 เดือน ไม่นับ]

* must provide value

- ไม่มี
- มี

ตอนแรกคลอดได้รับวัคซีนหรือไม่

* must provide value

- ไม่
- ใช่
- จำไม่ได้/ไม่แน่ใจ

คลอดที่โรงพยาบาลอะไร

* must provide value

ตอนอายุ 1-3 เดือน ได้รับวัคซีนหรือไม่

* must provide value

- ไม่
- ได้

มีสมุดบันทึกการฉีดวัคซีนมั้ย (เล่มสีชมพู)

* must provide value

- ไม่มี
- มี

การให้นมบุตรช่วง 3 เดือนแรกเกิด

* must provide value

- นมแม่
- นมผง
- ทั้ง 2 อย่าง

มีพี่น้องกี่คนในบ้าน [นับรวมเด็กคนนี้ด้วย]

* must provide value

เป็นลูกคนที่เท่าไหร่

* must provide value

ตอนแรกเกิด มีพี่น้องที่เข้าโรงเรียนแล้วกี่คน

* must provide value

ในบ้านมีเด็กอายุ <=12 ปี กี่คน

* must provide value

ตอนอายุ 0-3 เดือน เคยป่วยและรับการรักษาที่โรงพยาบาลเอกชนมั๊ย

* must provide value

○ ไม่
○ ใช่

บันทึก PDF ก่อนกดปุ่ม submit !!!

# - สำหรับ iPhone

02:45
◀ LINE
5G 49

redcap.dida.psu.ac.th

ไม่

ไม่ได้

ได้

res

มีสมุดบันทึกการฉีดวัคซีนมั้ย (เล่มสีชมพู)

* must provide value

- (●) ไม่มี
- ( ) มี

res

การให้นมบุตรช่วง 3 เดือนแรกเกิด

* must provide value

- (●) นมแม่
- ( ) นมผง
- ( ) ทั้ง 2 อย่าง

res

มีพี่น้องกี่คนในบ้าน [นับรวมเด็กคนนี้ด้วย]

* must provide value

1

เป็นลูกคนที่เท่าไหร่

* must provide value

1

ตอนแรกเกิด มีพี่น้องที่เข้าโรงเรียนแล้วกี่คน

* must provide value

0

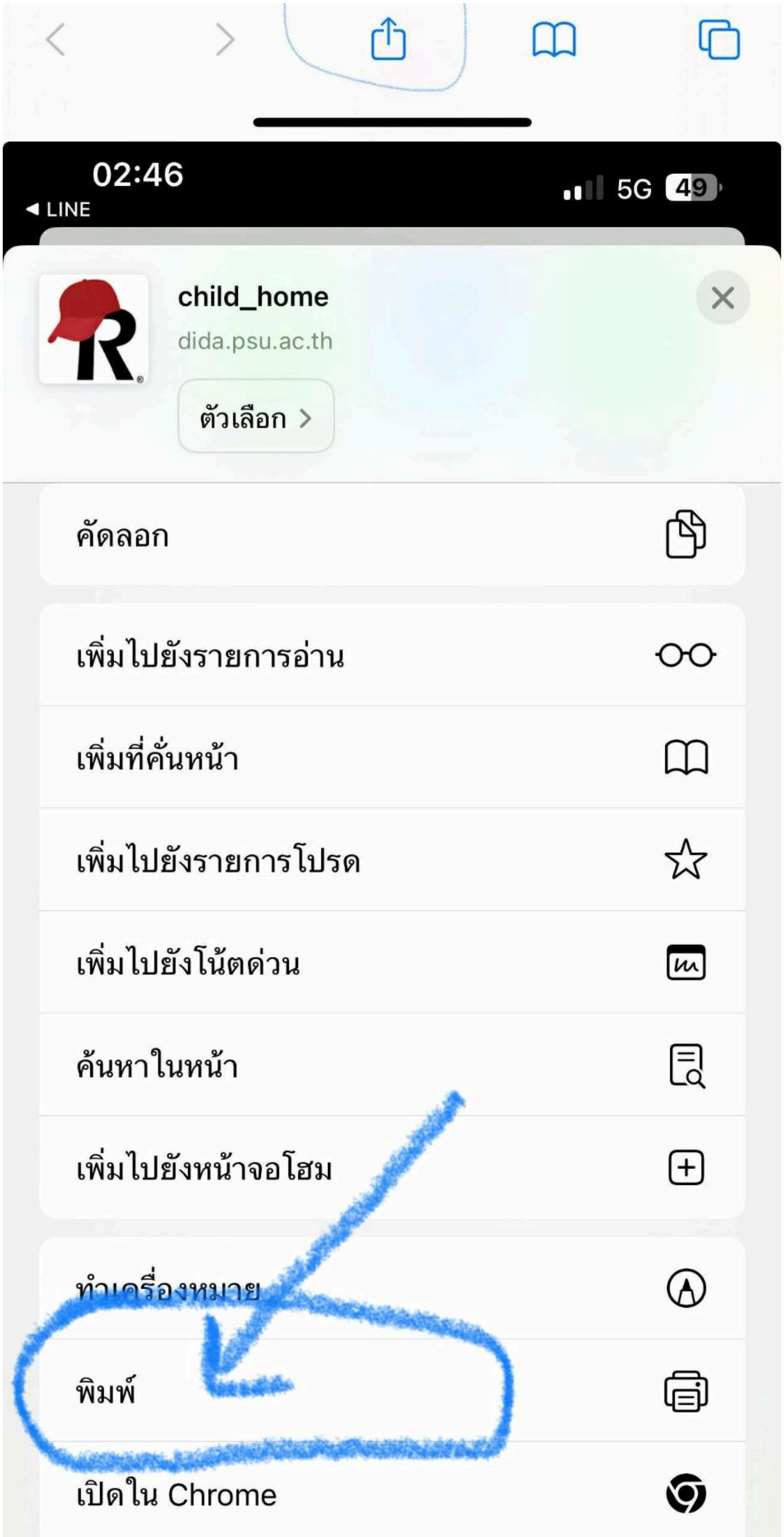
02:46
◀ LINE
5G 49
child_home
dida.psu.ac.th
ตัวเลือก >
คัดลอก
เพิ่มไปยังรายการอ่าน
เพิ่มที่คั่นหน้า
เพิ่มไปยังรายการโปรด
เพิ่มไปยังโน้ตด่วน
ค้นหาในหน้า
เพิ่มไปยังหน้าจอโฮม
ทำเครื่องหมาย
พิมพ์
เปิดใน Chrome

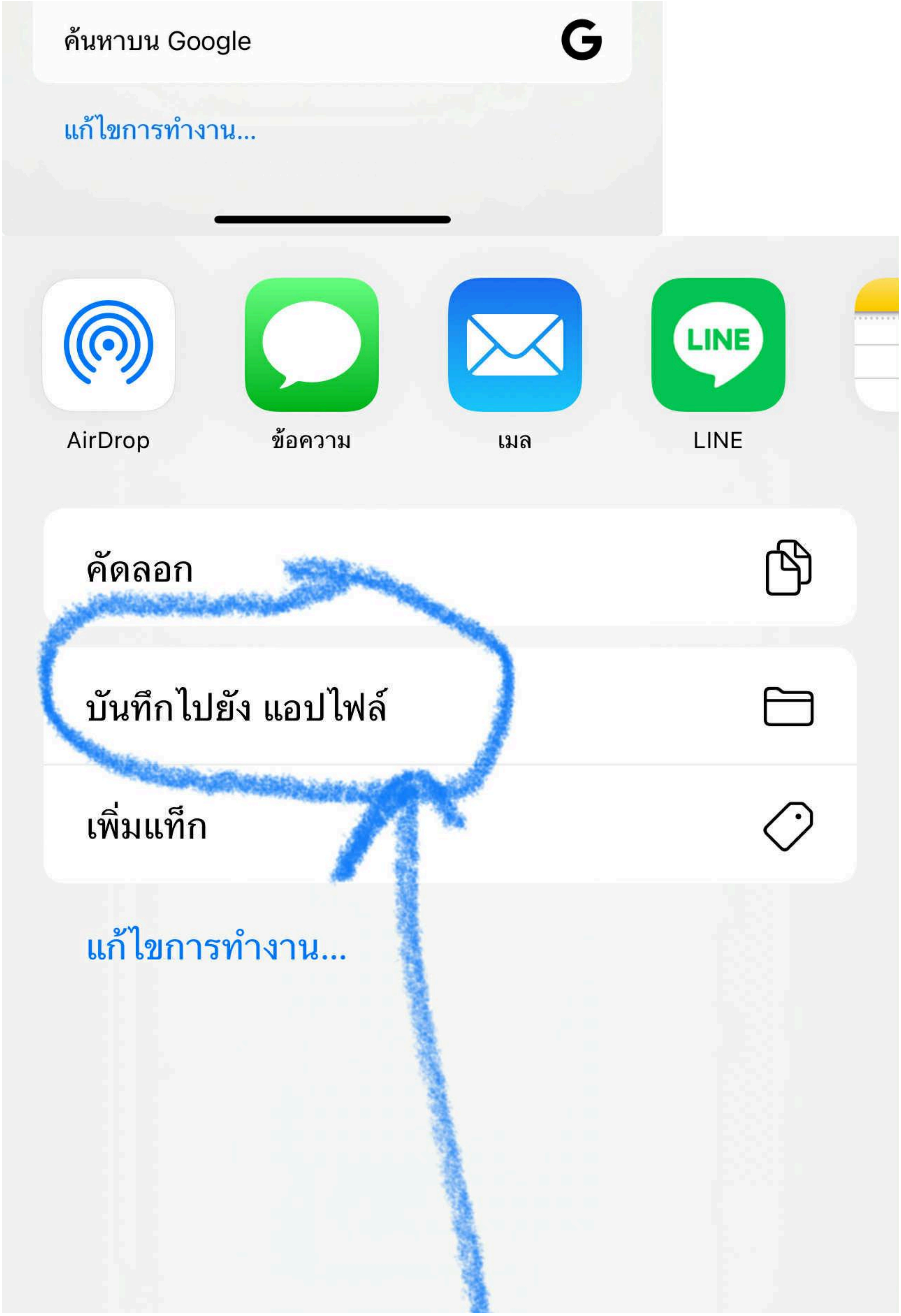
ค้นหาบน Google
แก้ไขการทำงาน...
AirDrop
ข้อความ
เมล
LINE
คัดลอก
บันทึกไปยัง แอปไฟล์
เพิ่มแท็ก
แก้ไขการทำงาน...

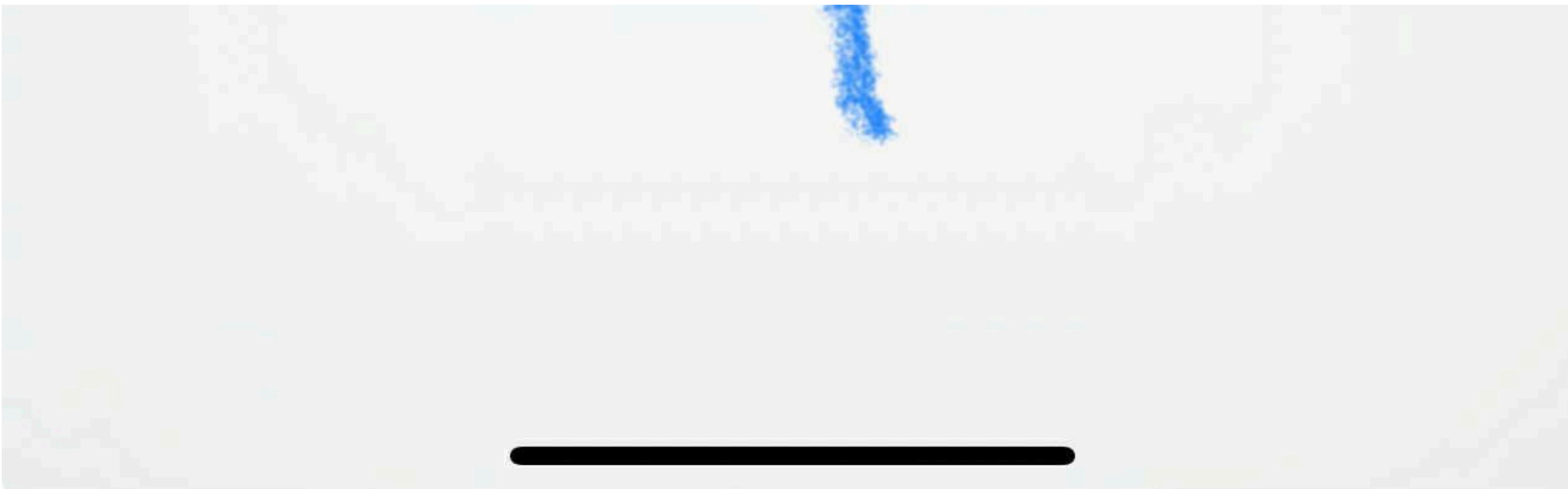

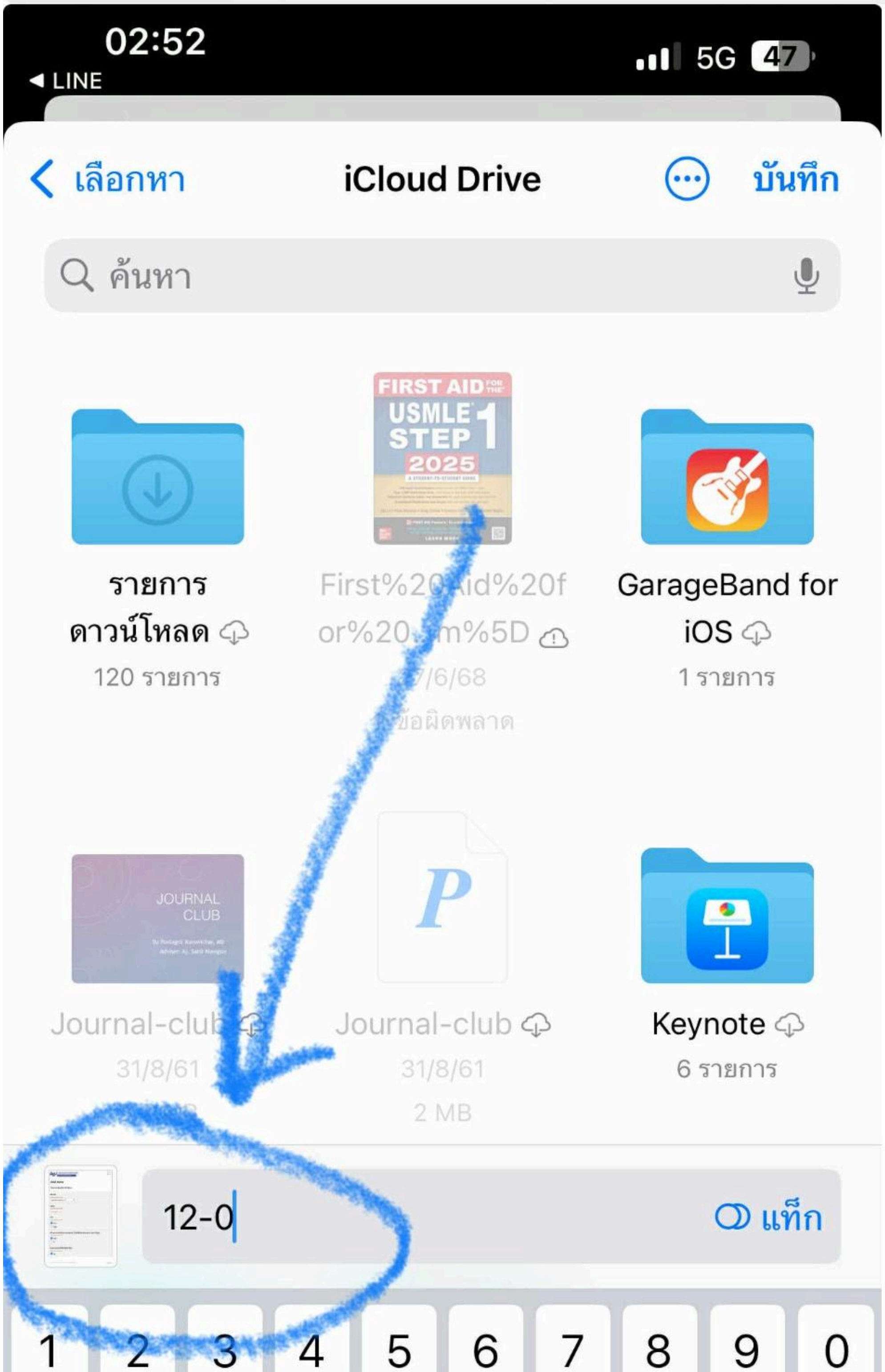
02:52
◀ LINE
5G 47
เลือกหา
iCloud Drive
บันทึก
ค้นหา
รายการ
ดาวน์โหลด
120 รายการ
GarageBand for
iOS
1 รายการ
Journal-club
31/8/61
Journal-club
31/8/61
2 MB
Keynote
6 รายการ
12-0
แท็ก
1 2 3 4 5 6 7 8 9 0

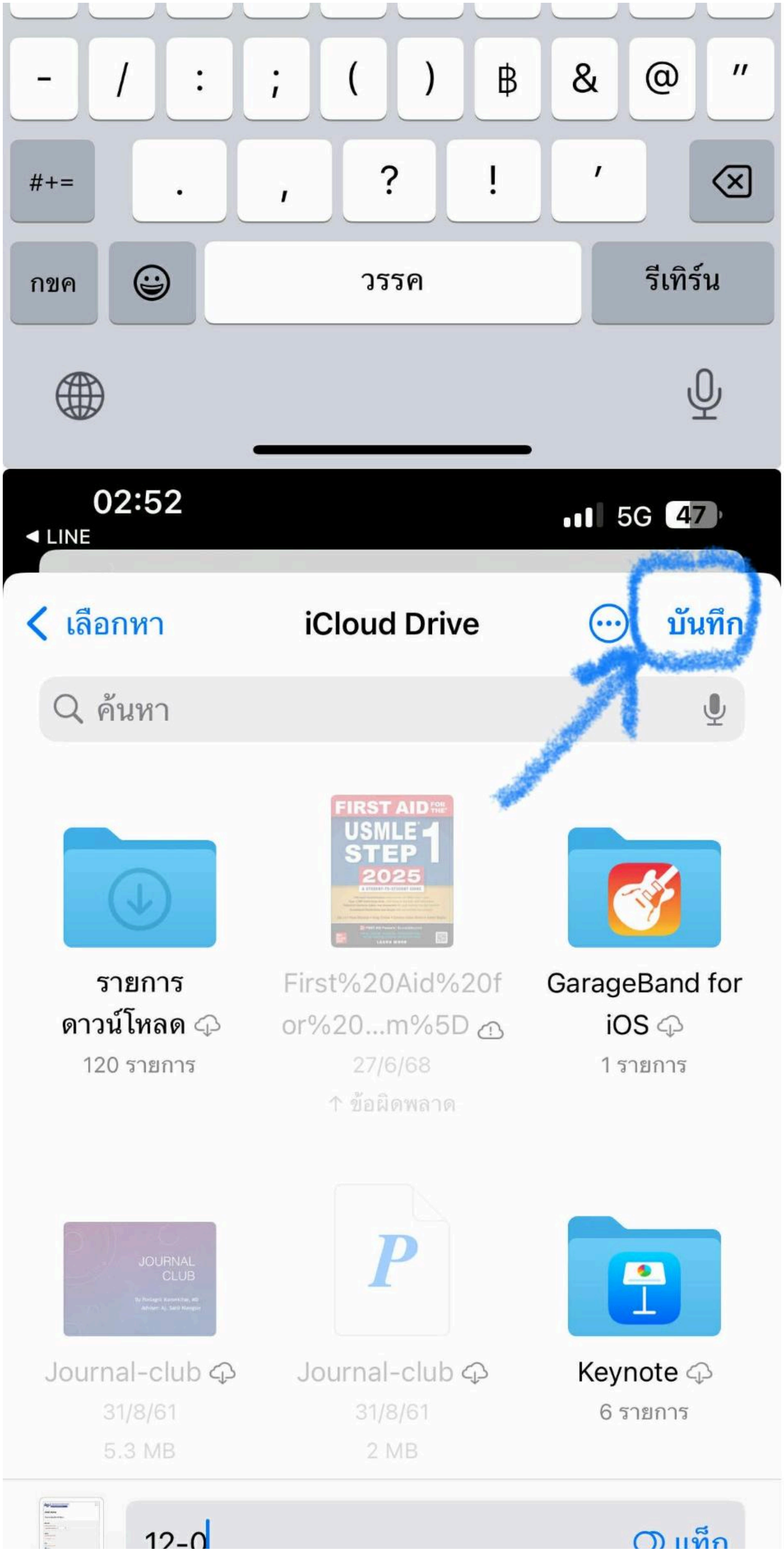
#+=
กขค
วรรค
รีเทิร์น
02:52
◀ LINE
5G 47
เลือกหา
iCloud Drive
บันทึก
ค้นหา
รายการ
ดาวน์โหลด
120 รายการ
First%20Aid%20f
or%20...m%5D
27/6/68
↑ ข้อผิดพลาด
GarageBand for
iOS
1 รายการ
Journal-club
31/8/61
5.3 MB
Journal-club
31/8/61
2 MB
Keynote
6 รายการ
12-0
แท็ก

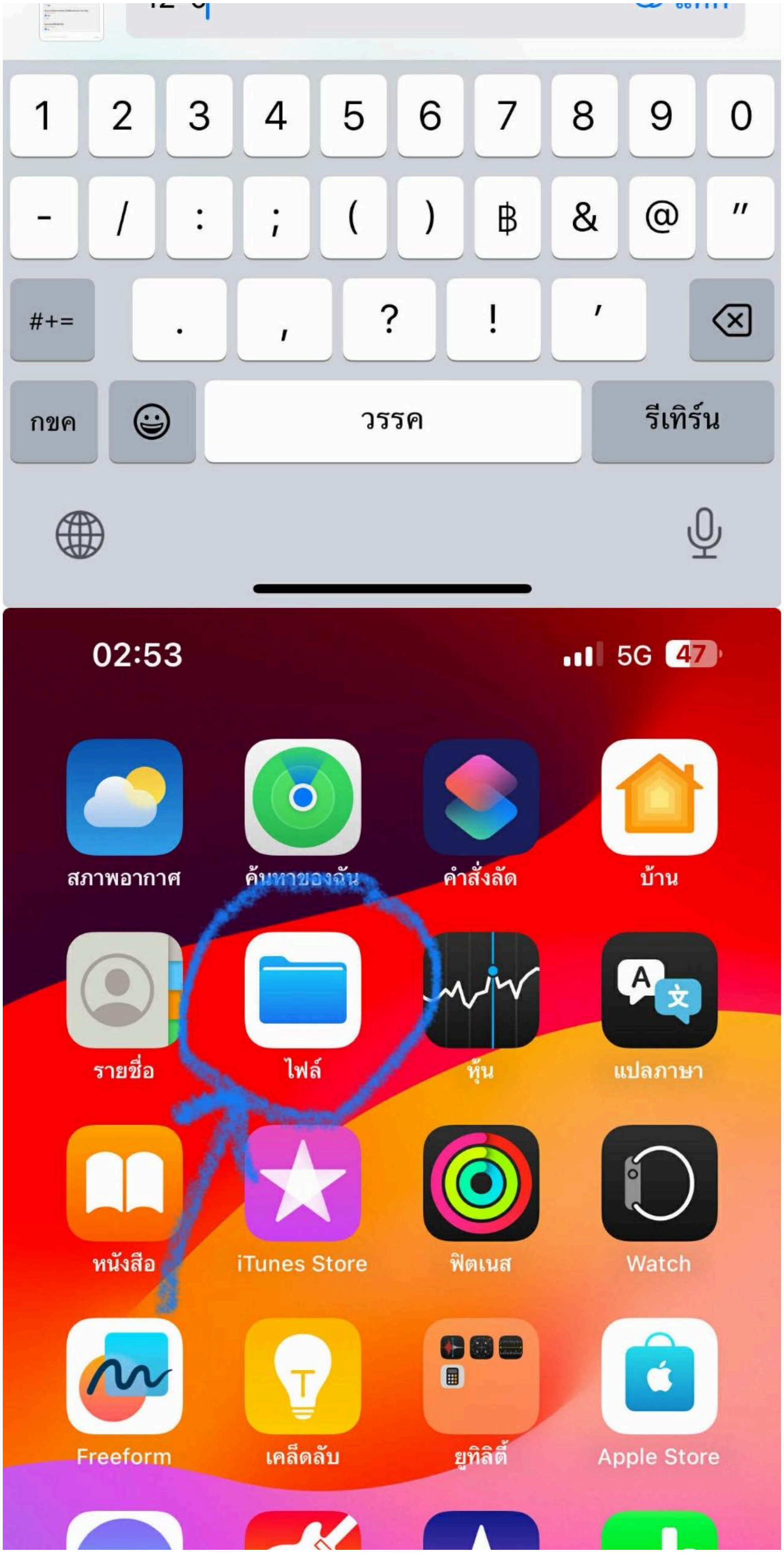
1 2 3 4 5 6 7 8 9 0
- / : ; ( ) ฿ & @ "
#+= . , ? ! '
กขค
วรรค
รีเทิร์น
02:53
5G 47
สภาพอากาศ
ค้นหาของฉัน
คำสั่งลัด
บ้าน
รายชื่อ
ไฟล์
หุ้น
แปลภาษา
หนังสือ
iTunes Store
ฟิตเนส
Watch
Freeform
เคล็ดลับ
ยูทิลิตี้
Apple Store

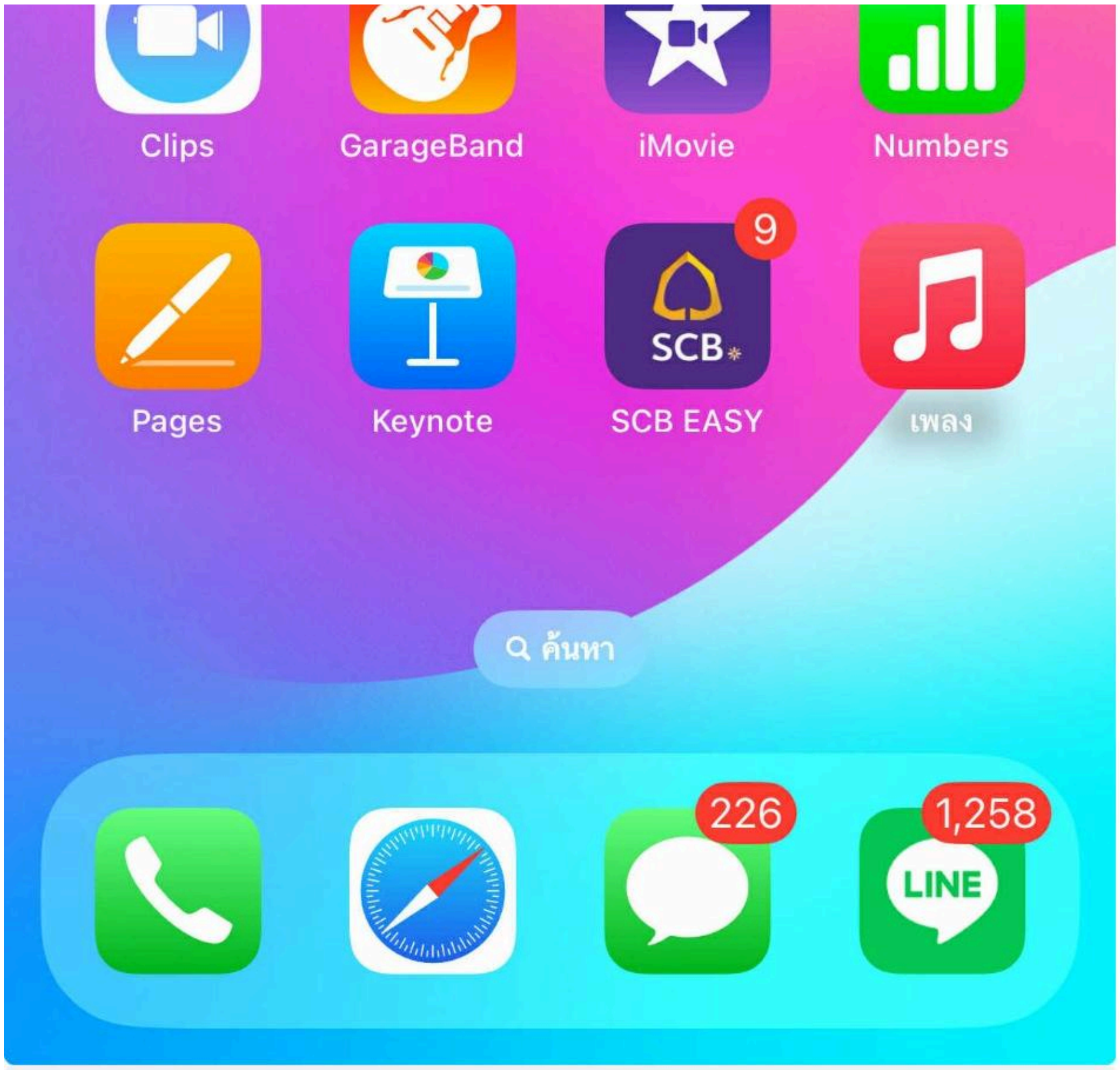

02:53 5G 47

12-0 เสร็จสิ้น

Department of Epidemiology
Faculty of Medicine, Prince of Songkla University
WHO Collaborating Centre for Research and Training on Epidemiology

child_home

โปรดกรอกข้อมูลที่จำเป็นให้ครบ

เลือก ID
* must provide value
แวฟาxxxxx แวมาxx, 2-7

วันเกิด
* must provide value
10-10-202 D-M-Y

เพศ
* must provide value
ชาย

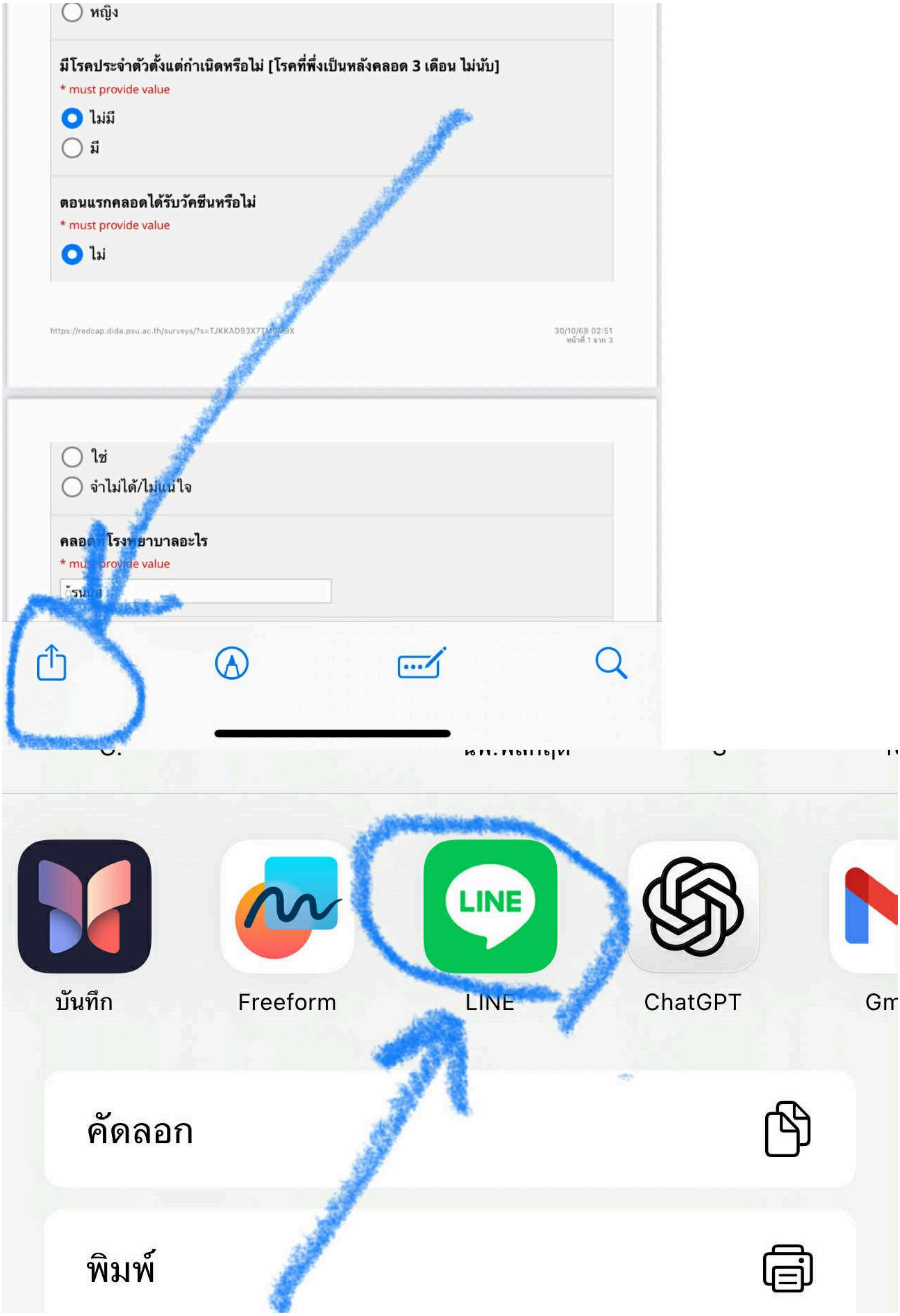
หญิง
มีโรคประจำตัวตั้งแต่กำเนิดหรือไม่ [โรคที่พึ่งเป็นหลังคลอด 3 เดือน ไม่นับ]
* must provide value
ไม่มี
มี
ตอนแรกคลอดได้รับวัคซีนหรือไม่
* must provide value
ไม่
30/10/68 02:51
ใช่
จำไม่ได้/ไม่แน่ใจ
บันทึก
Freeform
LINE
ChatGPT
คัดลอก
พิมพ์

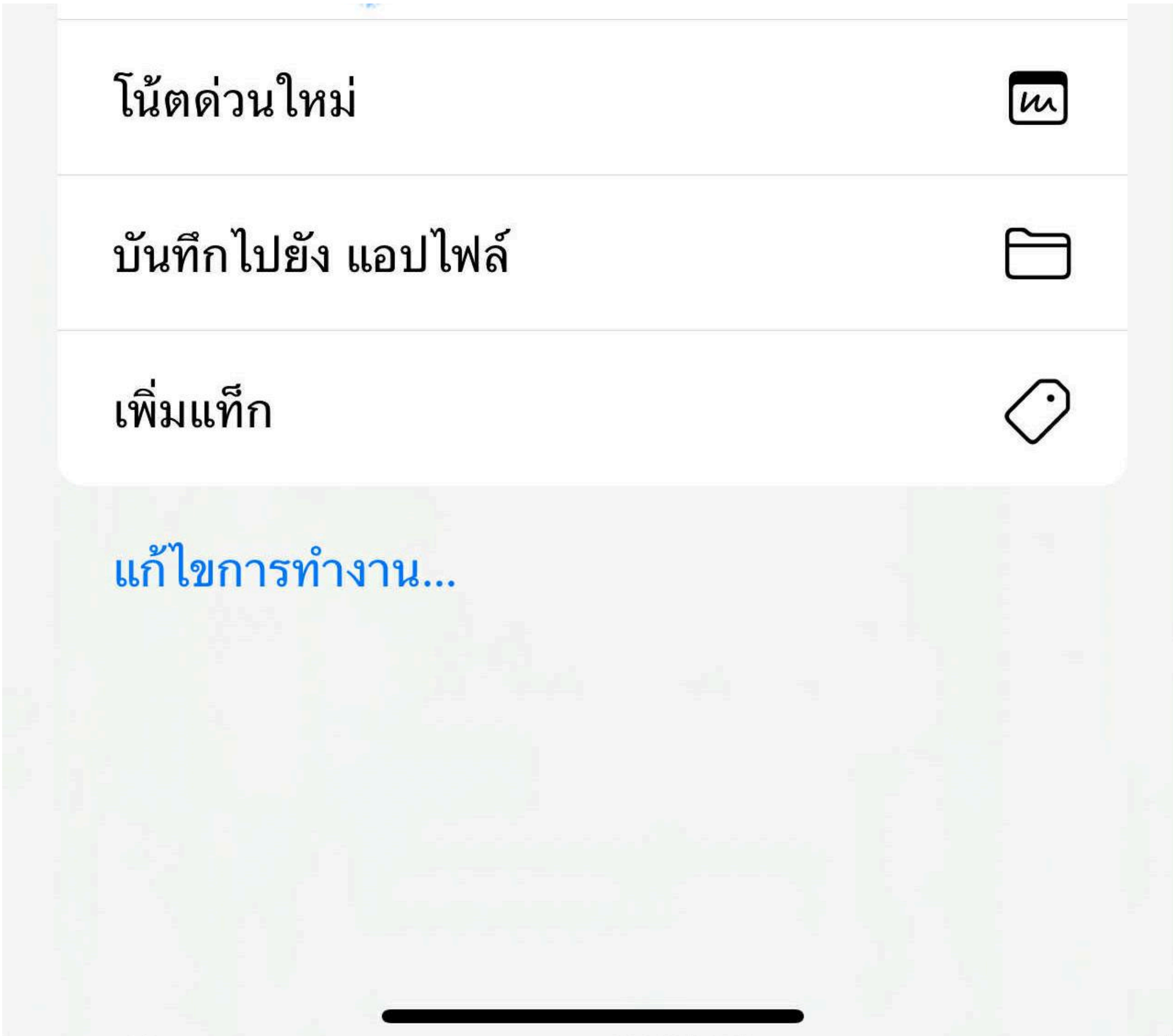

- สำหรับ Android

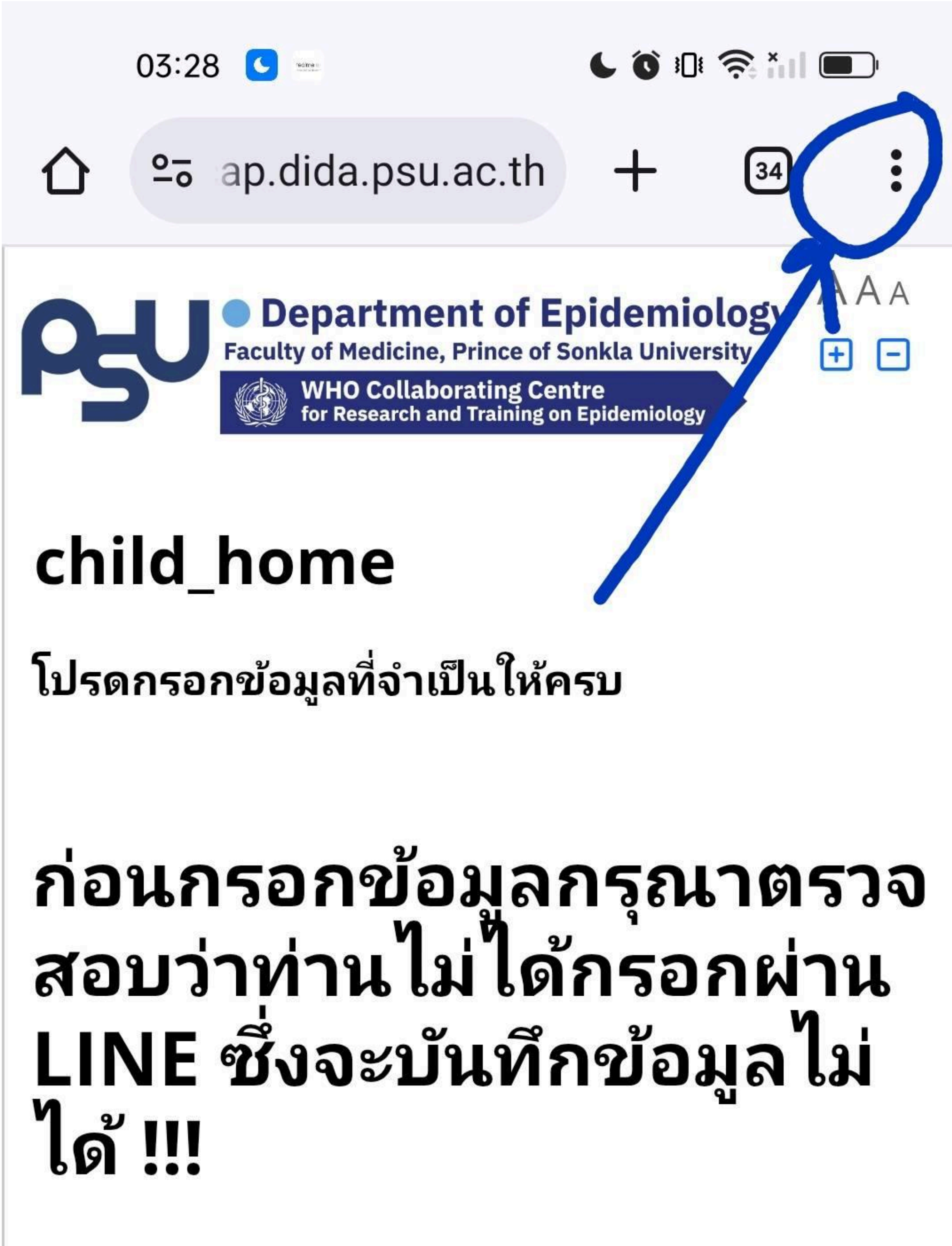
03:28
ap.dida.psu.ac.th
34
Department of Epidemiology
Faculty of Medicine, Prince of Sonkla University
WHO Collaborating Centre
for Research and Training on Epidemiology
child_home
โปรดกรอกข้อมูลที่จำเป็นให้ครบ
ก่อนกรอกข้อมูลกรุณาตรวจสอบว่าท่านไม่ได้กรอกผ่าน LINE ซึ่งจะบันทึกข้อมูลไม่ได้ !!!

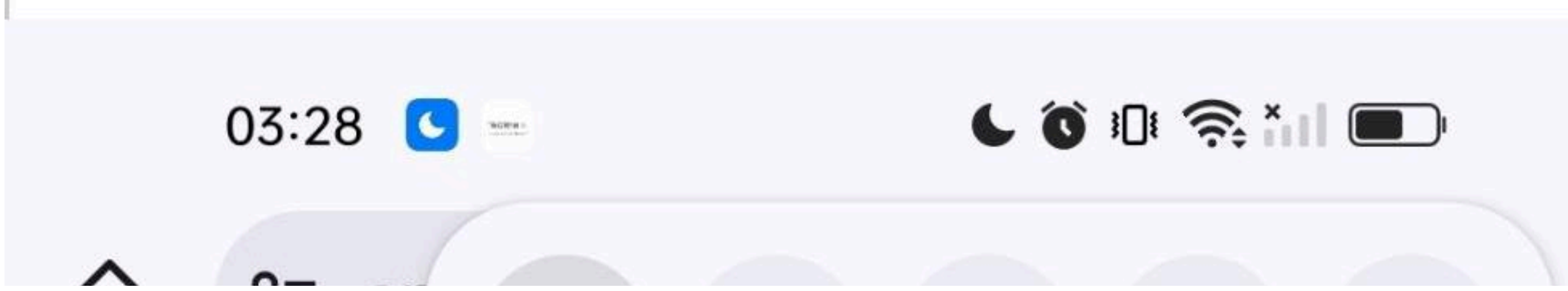
03:28

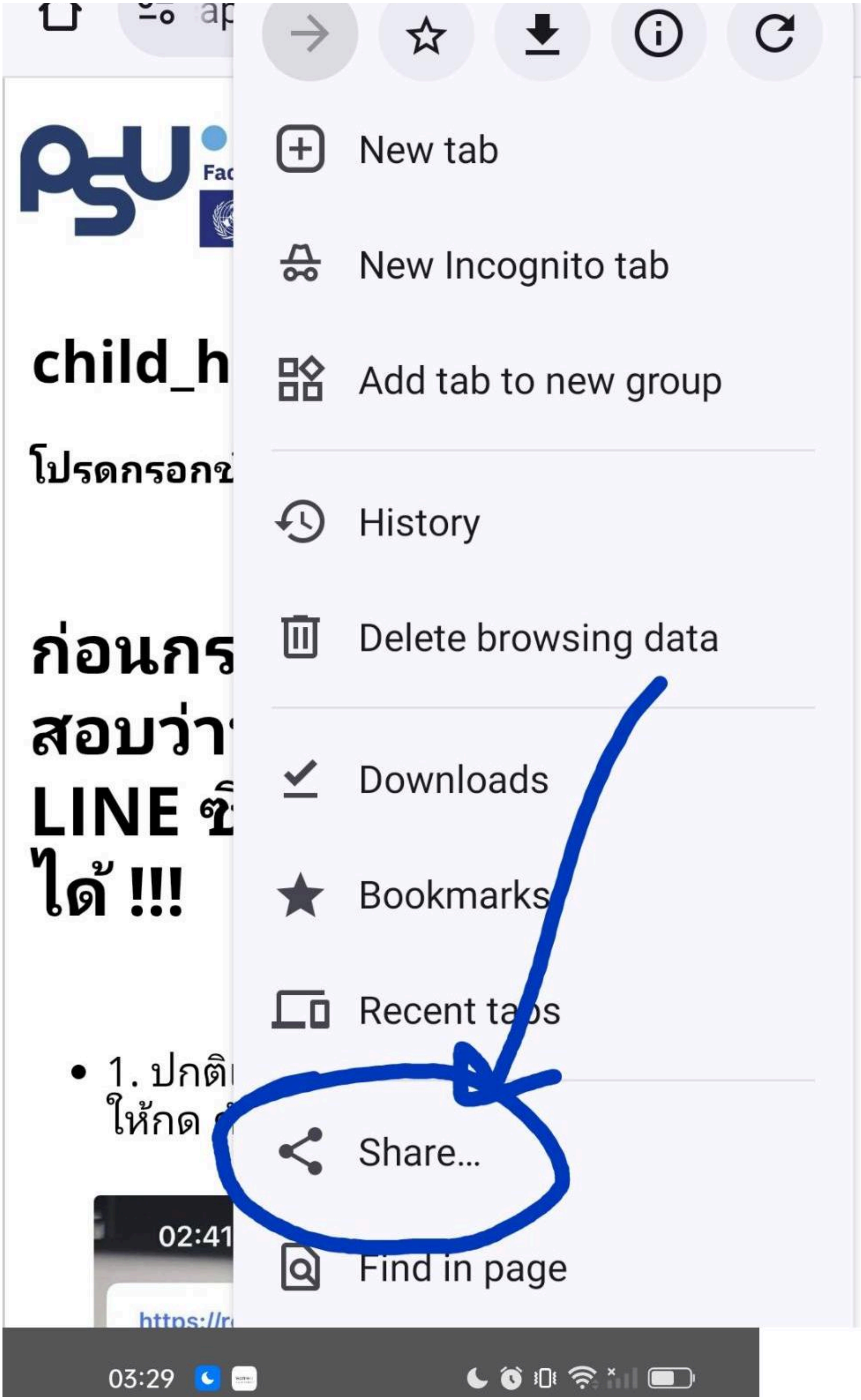
New tab
New Incognito tab
Add tab to new group
History
Delete browsing data
Downloads
Bookmarks
Recent tabs
Share...
Find in page
child_h
โปรดกรอก
ก่อนกร
สอบว่า
LINE ซ
ได้ !!!
1. ปกติ
ให้กด
02:41
03:29

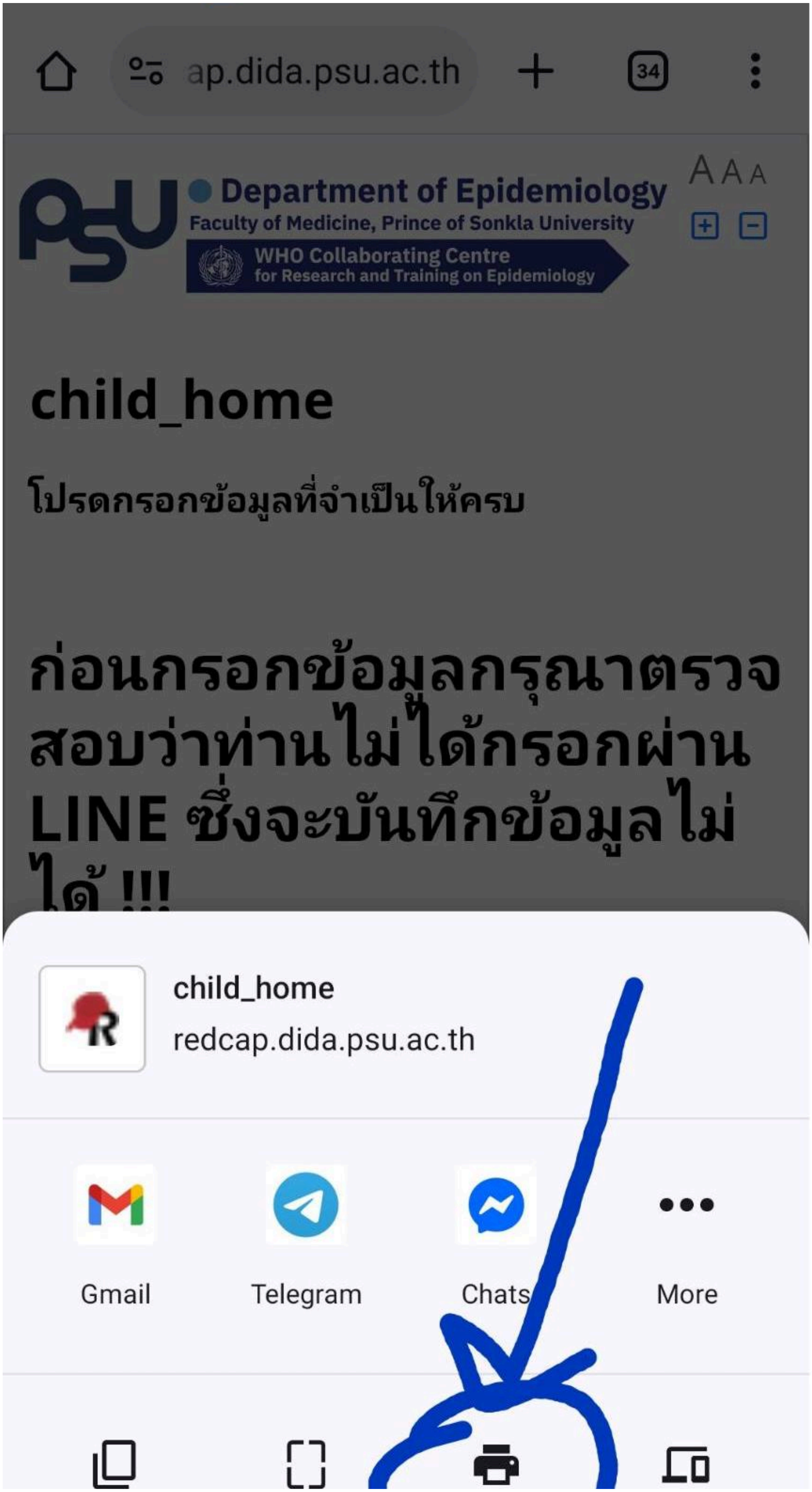
ap.dida.psu.ac.th
34
AAA
Department of Epidemiology
Faculty of Medicine, Prince of Sonkla University
WHO Collaborating Centre
for Research and Training on Epidemiology
child_home
โปรดกรอกข้อมูลที่จำเป็นให้ครบ
ก่อนกรอกข้อมูลกรุณาตรวจสอบว่าท่านไม่ได้กรอกผ่าน LINE ซึ่งจะบันทึกข้อมูลไม่ได้ !!!
child_home
redcap.dida.psu.ac.th
Gmail
Telegram
Chats
More

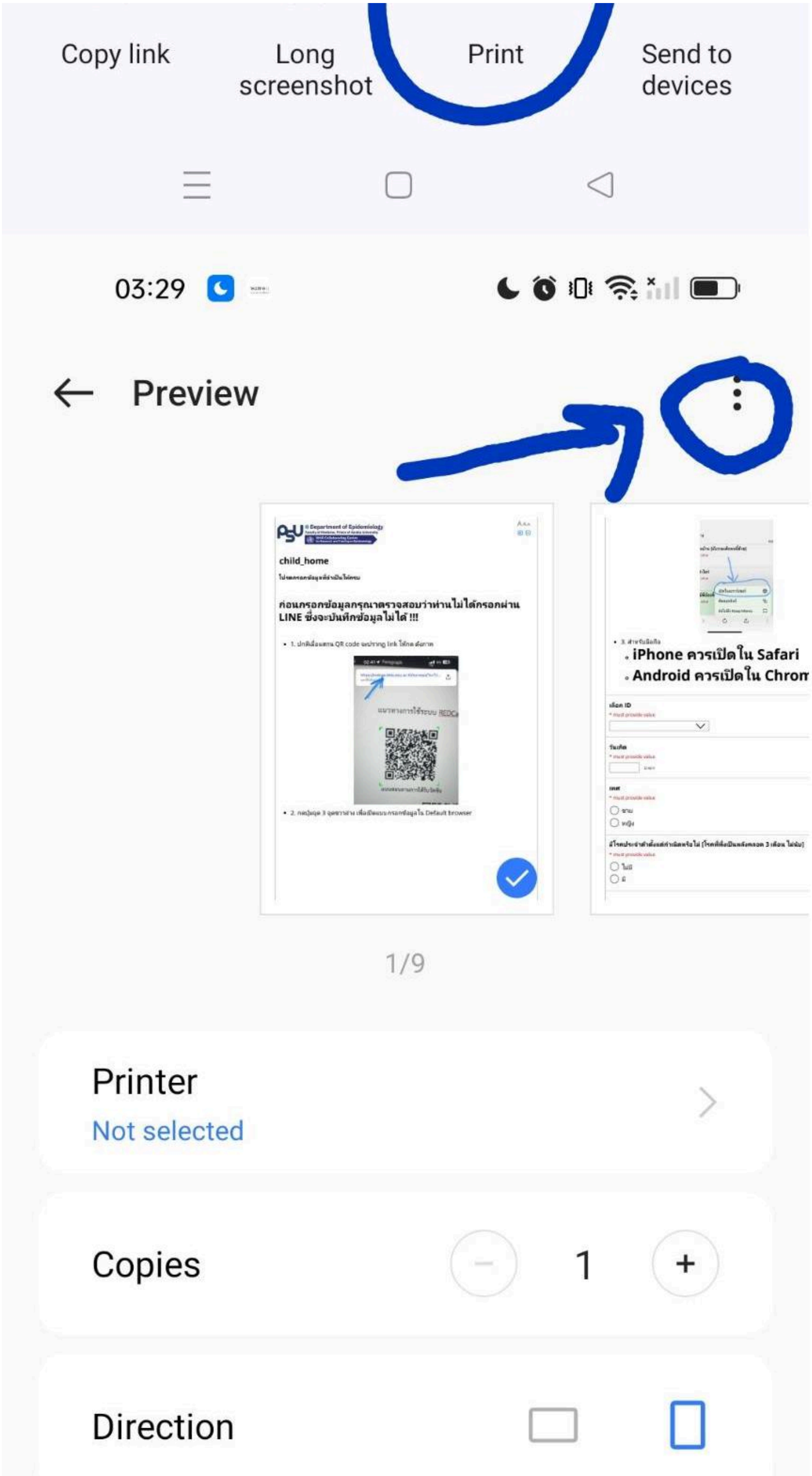
Copy link
Long screenshot
Print
Send to devices
03:29
Preview
child_home
ก่อนกรอกข้อมูลกรุณาตรวจสอบว่าท่านไม่ได้กรอกผ่าน LINE ซึ่งจะบันทึกข้อมูลไม่ได้ !!!
iPhone ควรเปิดใน Safari
Android ควรเปิดใน Chrom
1/9
Printer
Not selected
Copies
1
Direction

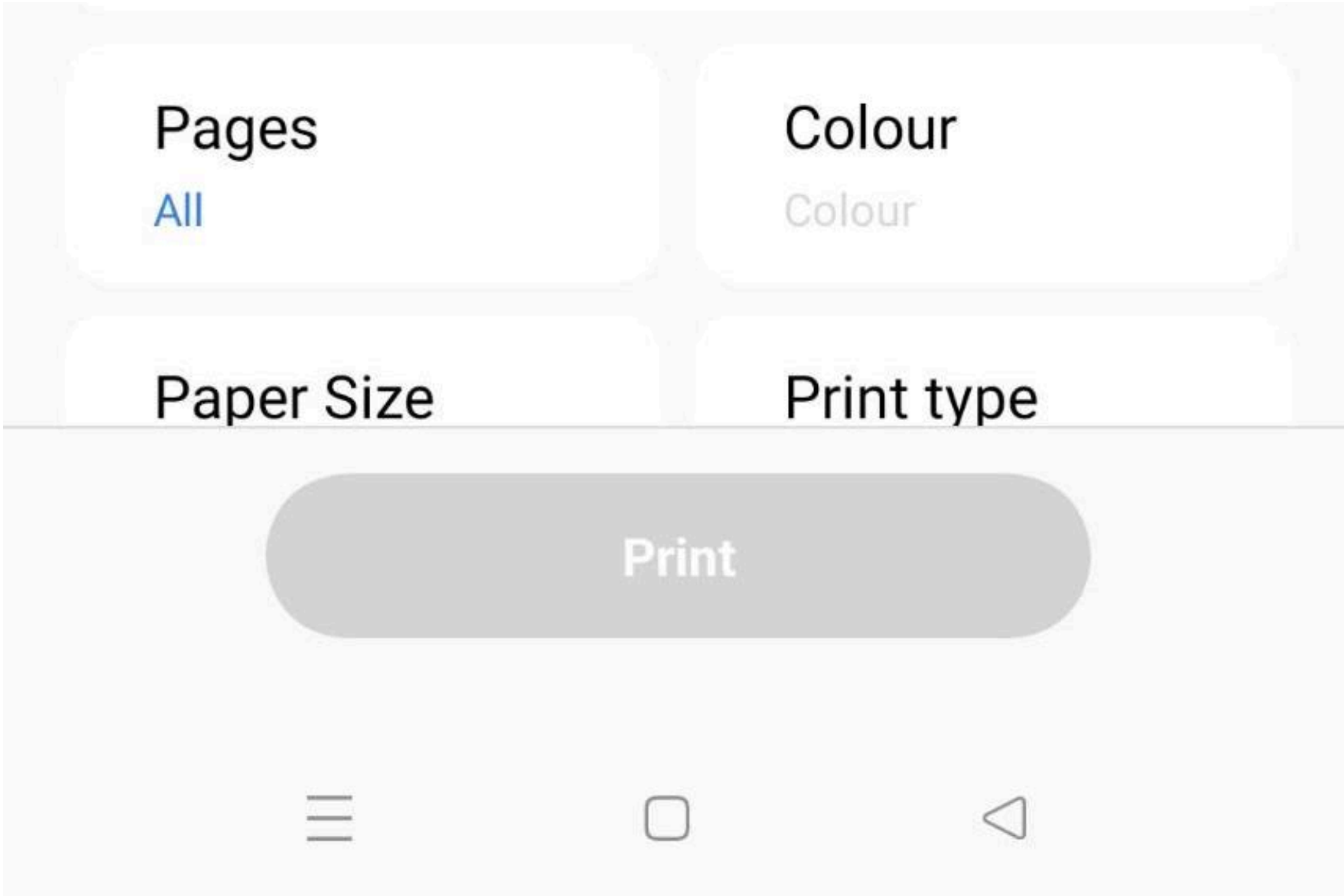
Pages
All
Colour
Colour
Paper Size
Print type
Print

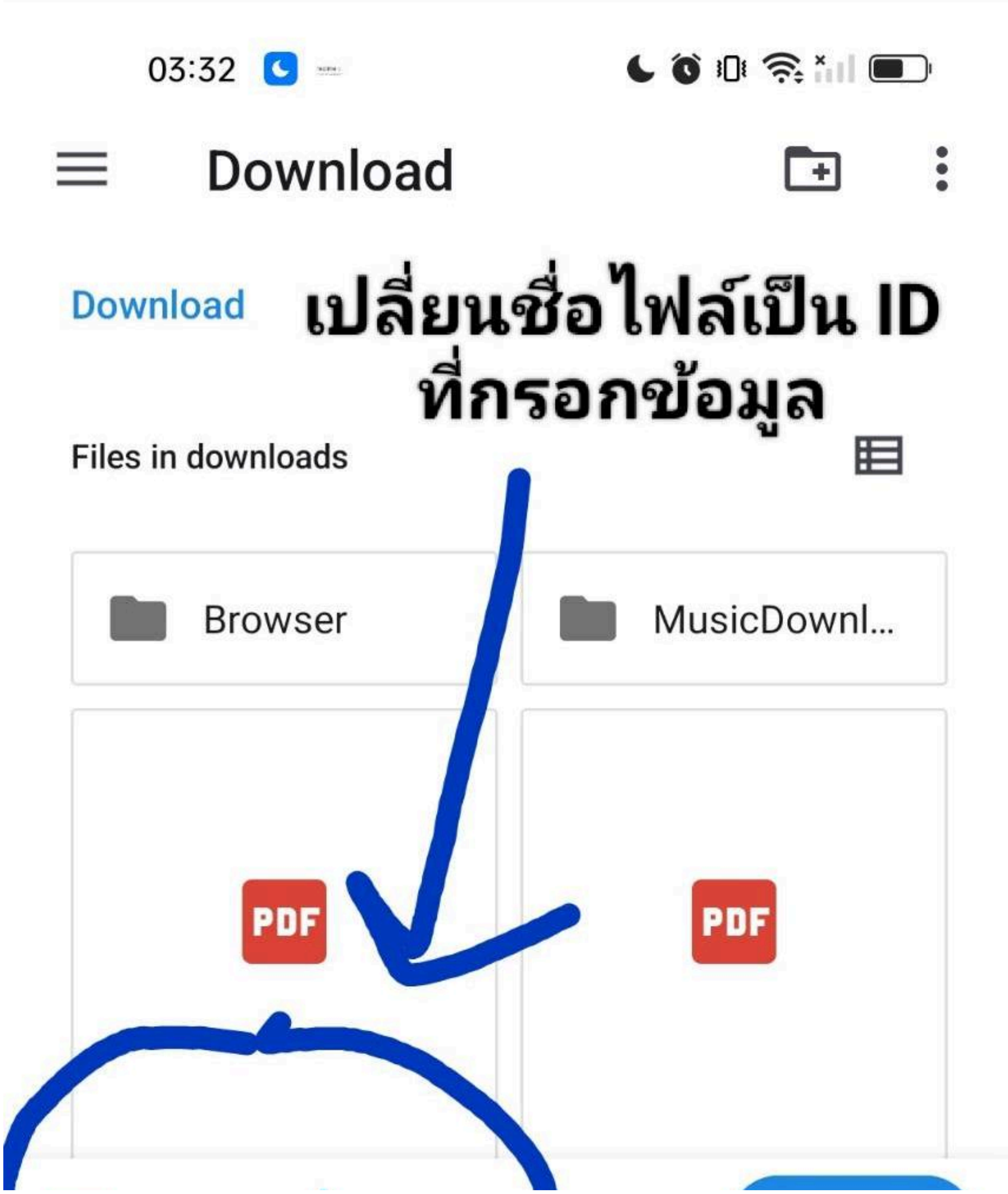
03:32
Download
Download
เปลี่ยนชื่อไฟล์เป็น ID
ที่กรอกข้อมูล
Files in downloads
Browser
MusicDownl...
PDF
PDF

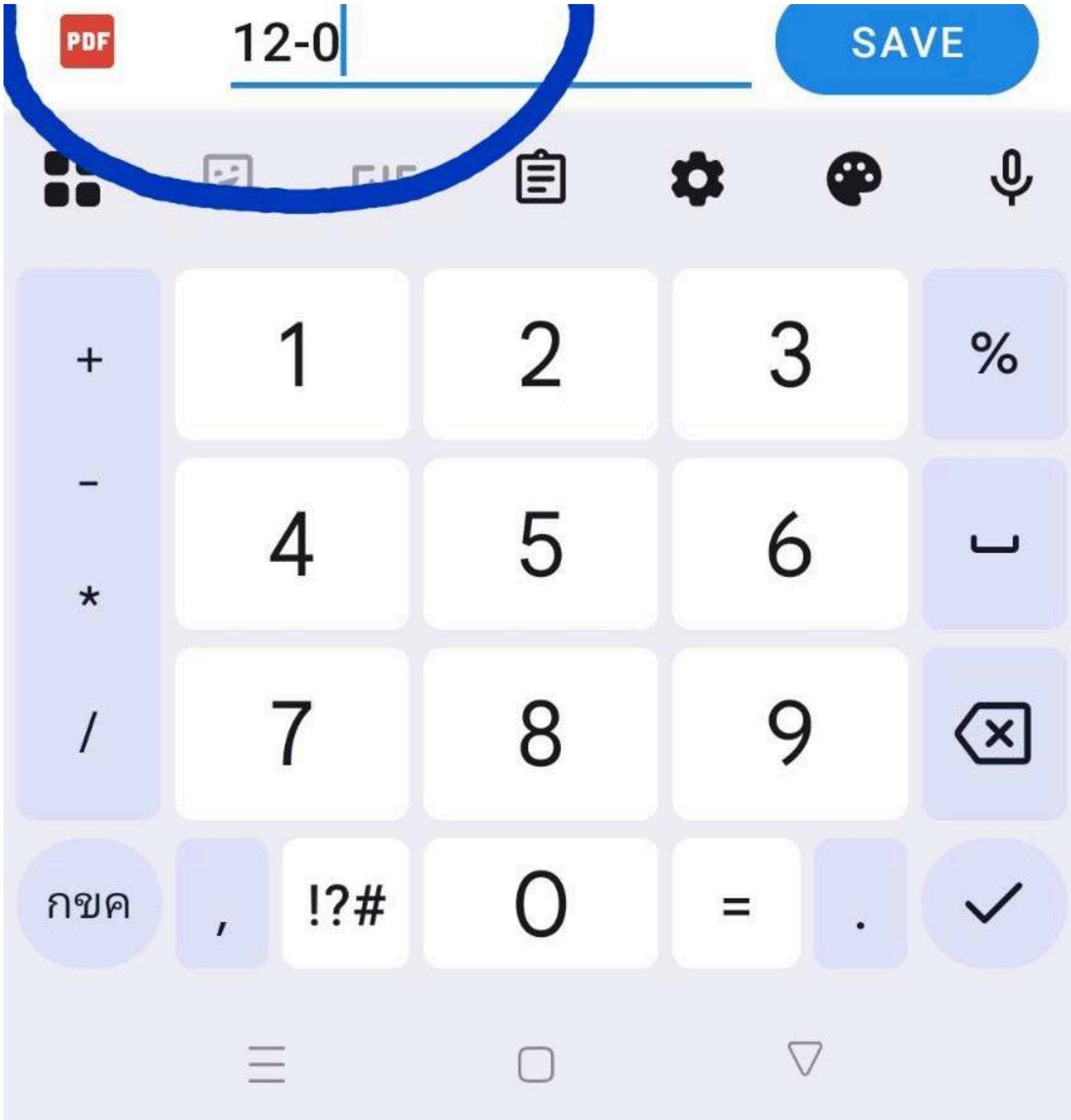
PDF
12-0
SAVE
1
2
3
%
4
5
6
7
8
9
กขค
,
!?#
0
=
.

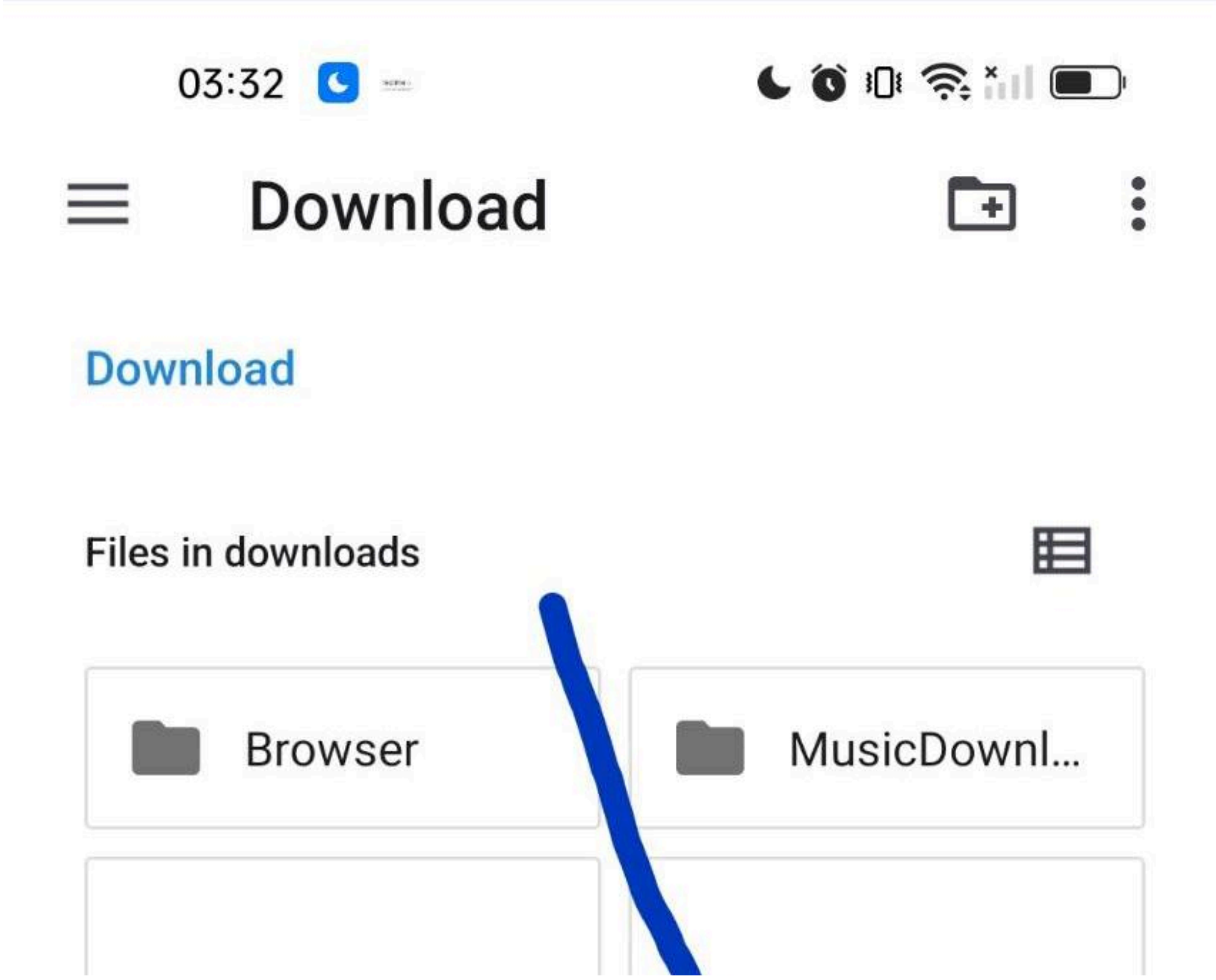
03:32
Download
Download
Files in downloads
Browser
MusicDownl...

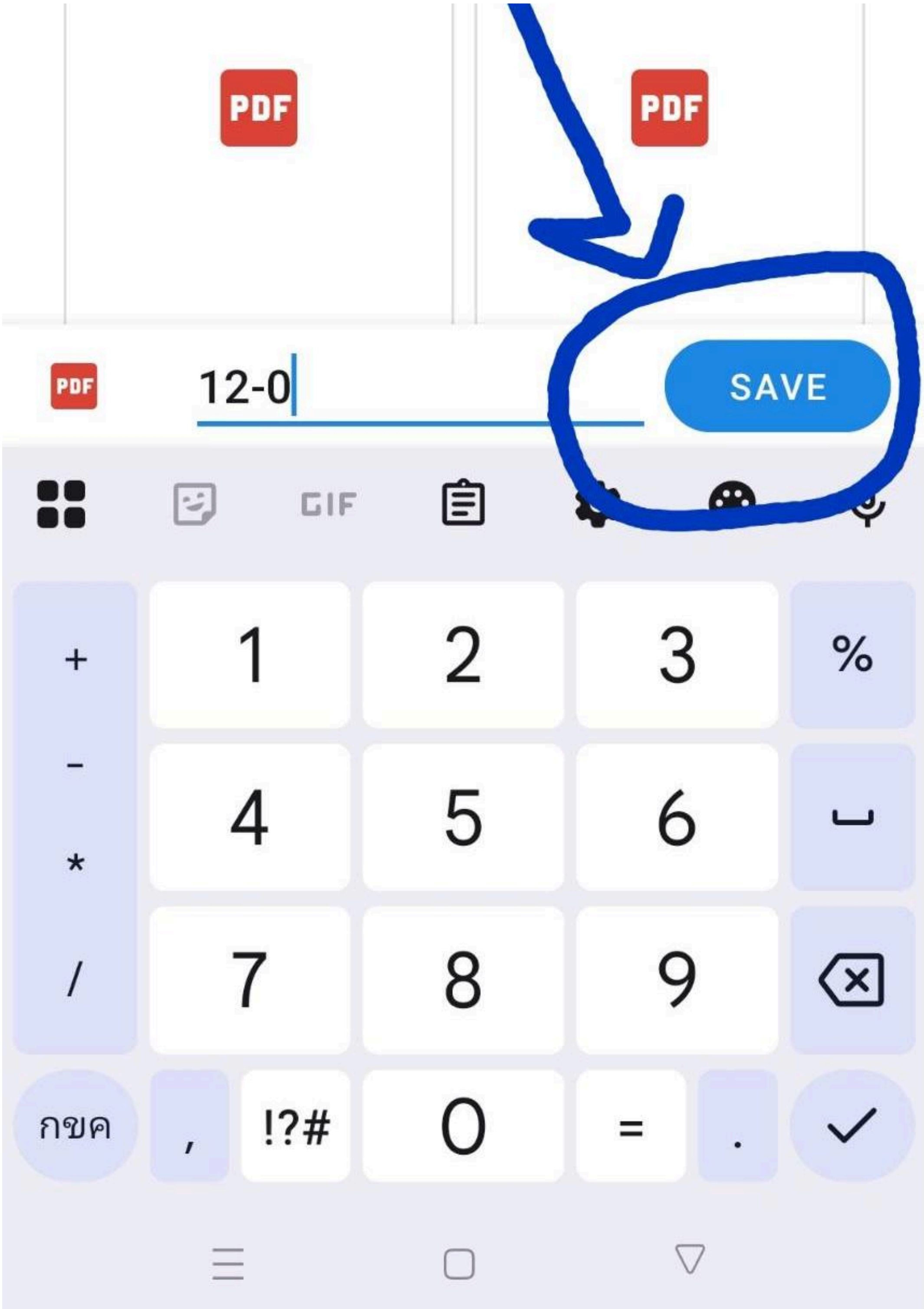
PDF
PDF
PDF
12-0
SAVE
GIF
+ 1 2 3 %
- 4 5 6
* 7 8 9
/
กขค , !?# 0 = .

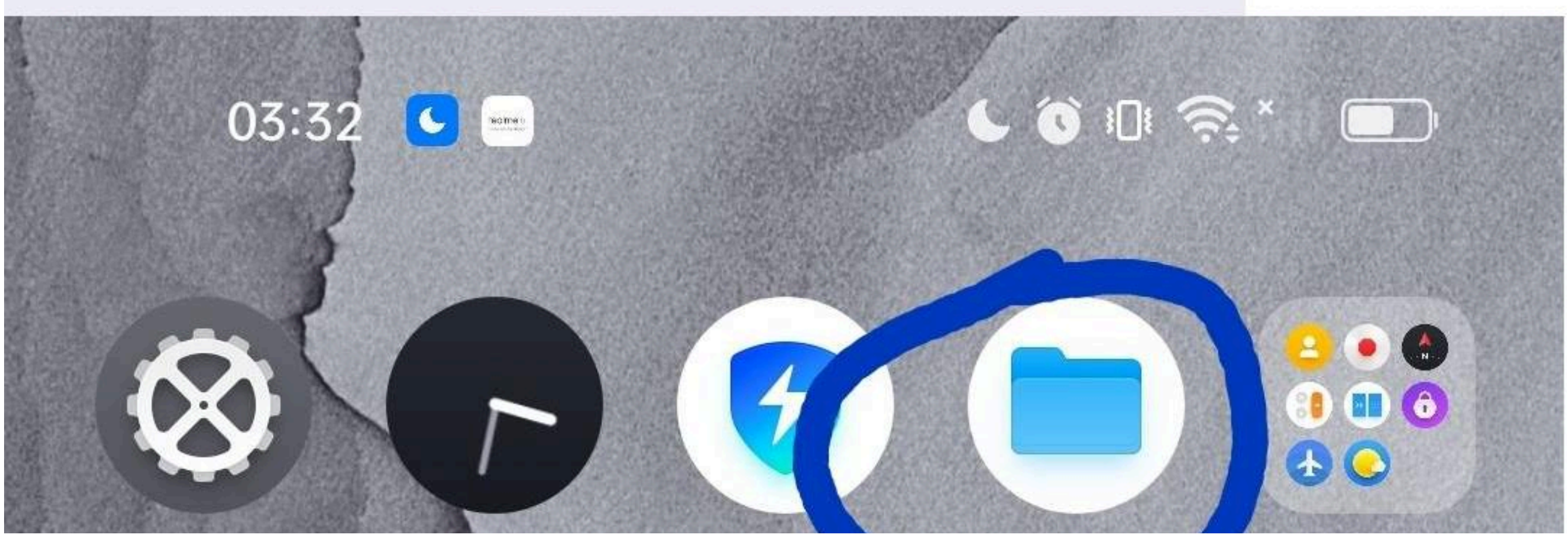
03:32

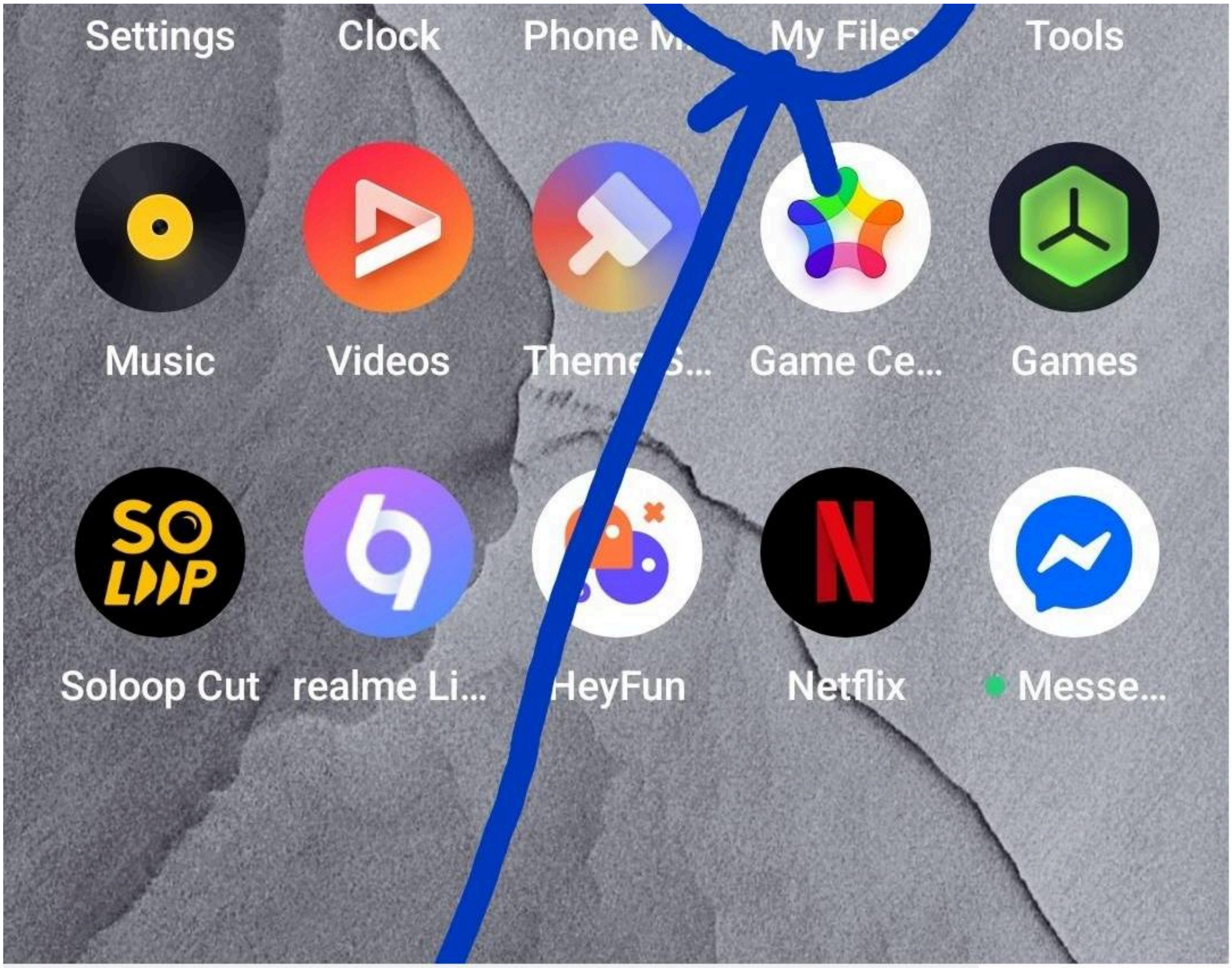
Settings
Clock
Phone M...
My Files
Tools
Music
Videos
Theme S...
Game Ce...
Games
Soloop Cut
realme Li...
HeyFun
Netflix
Messe...

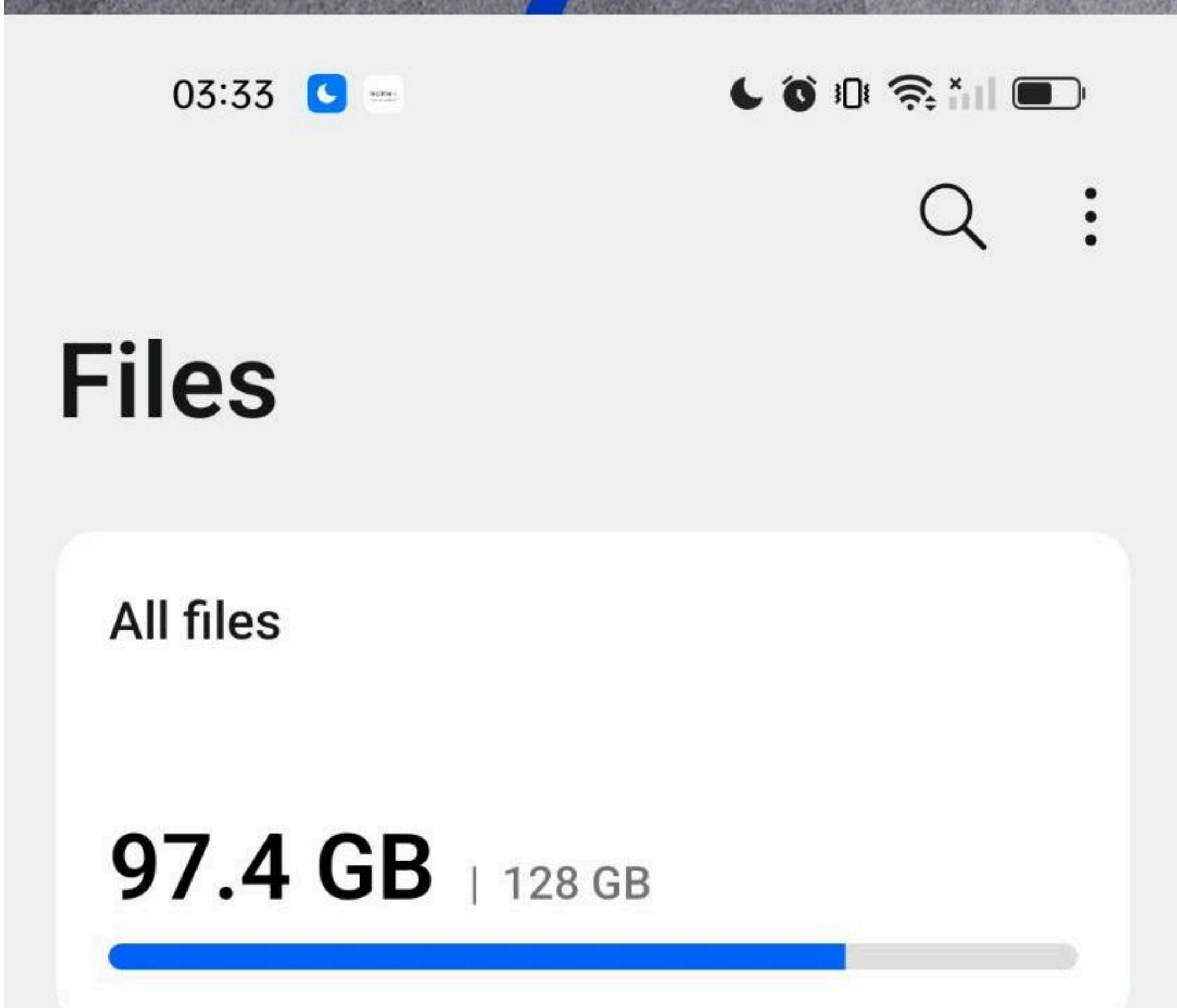
03:33
Files
All files
97.4 GB | 128 GB

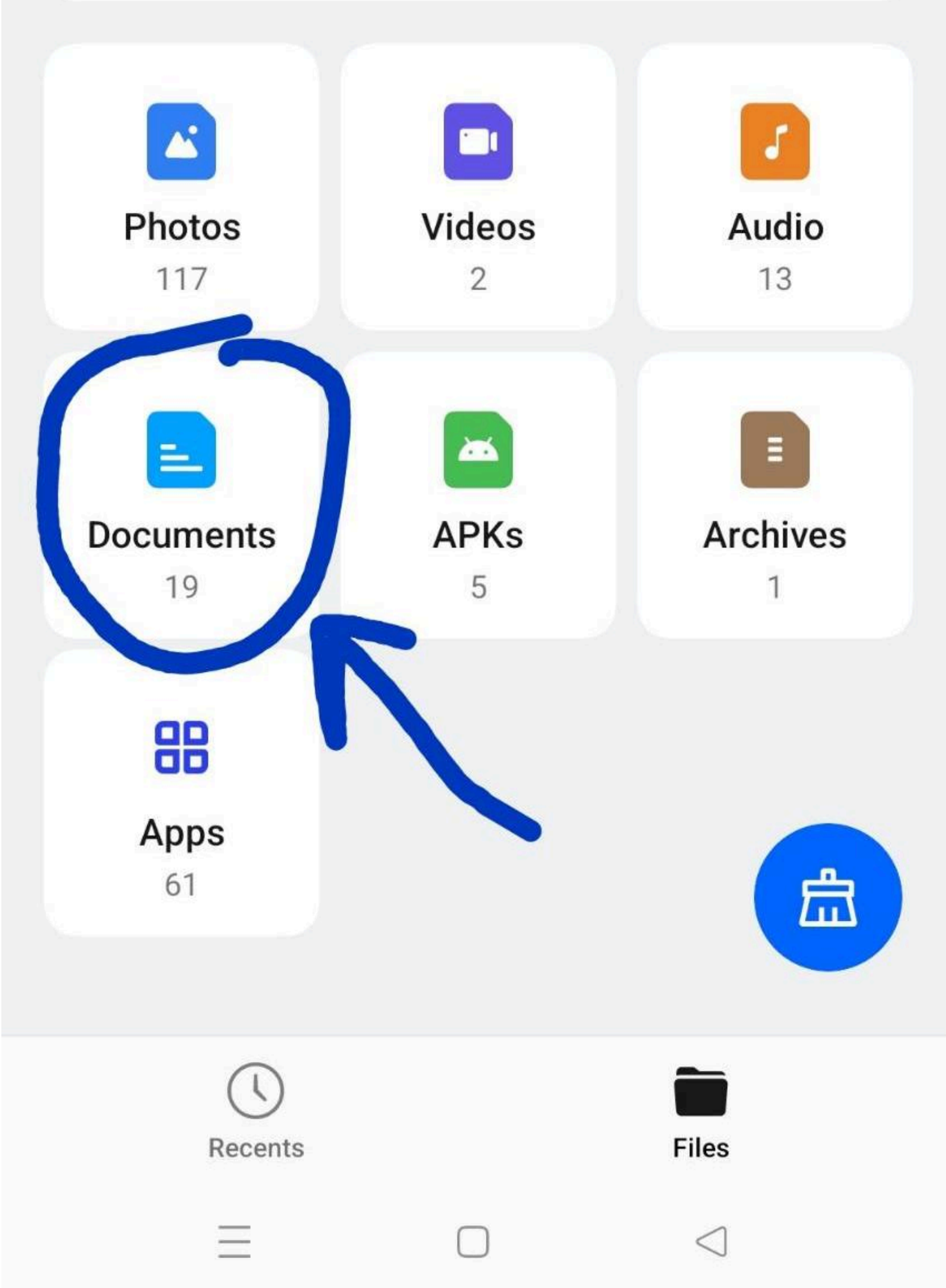
Photos
117
Videos
2
Audio
13
Documents
19
APKs
5
Archives
1
Apps
61
Recents
Files

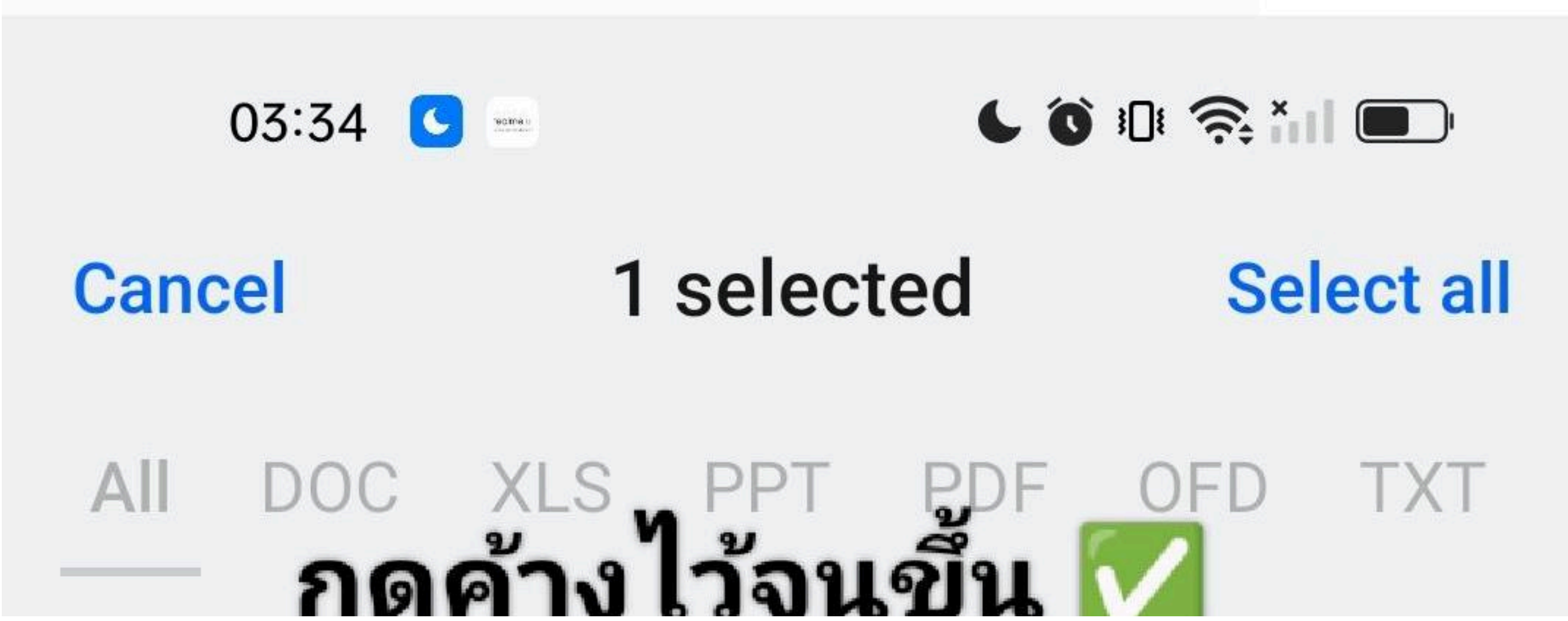
03:34
Cancel
1 selected
Select all
All
DOC
XLS
PPT
PDF
OFD
TXT
กดค้างไว้จนขึ้น ✅

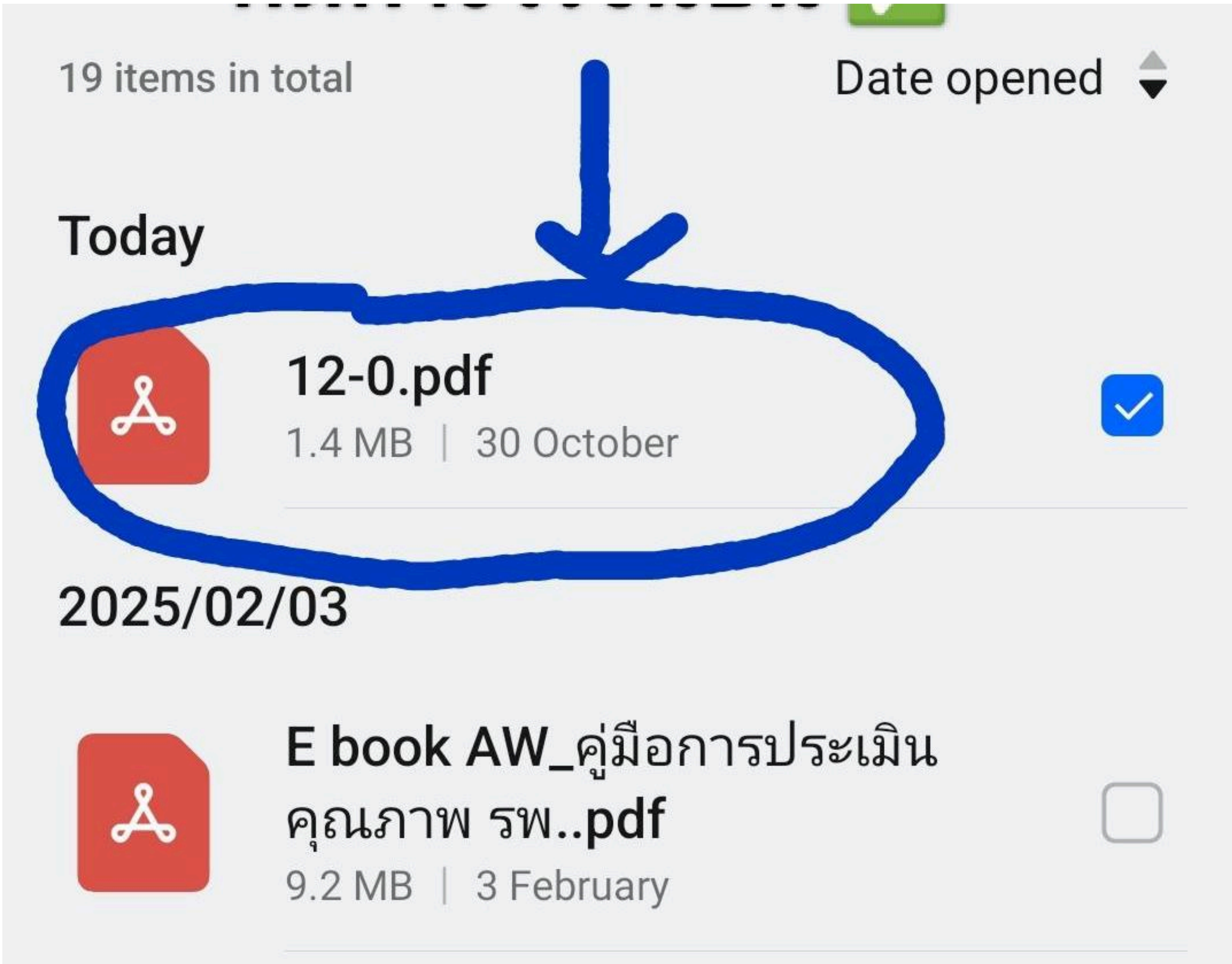
19 items in total
Date opened
Today
12-0.pdf
1.4 MB | 30 October
2025/02/03
E book AW_คู่มือการประเมินคุณภาพ รพ..pdf
9.2 MB | 3 February

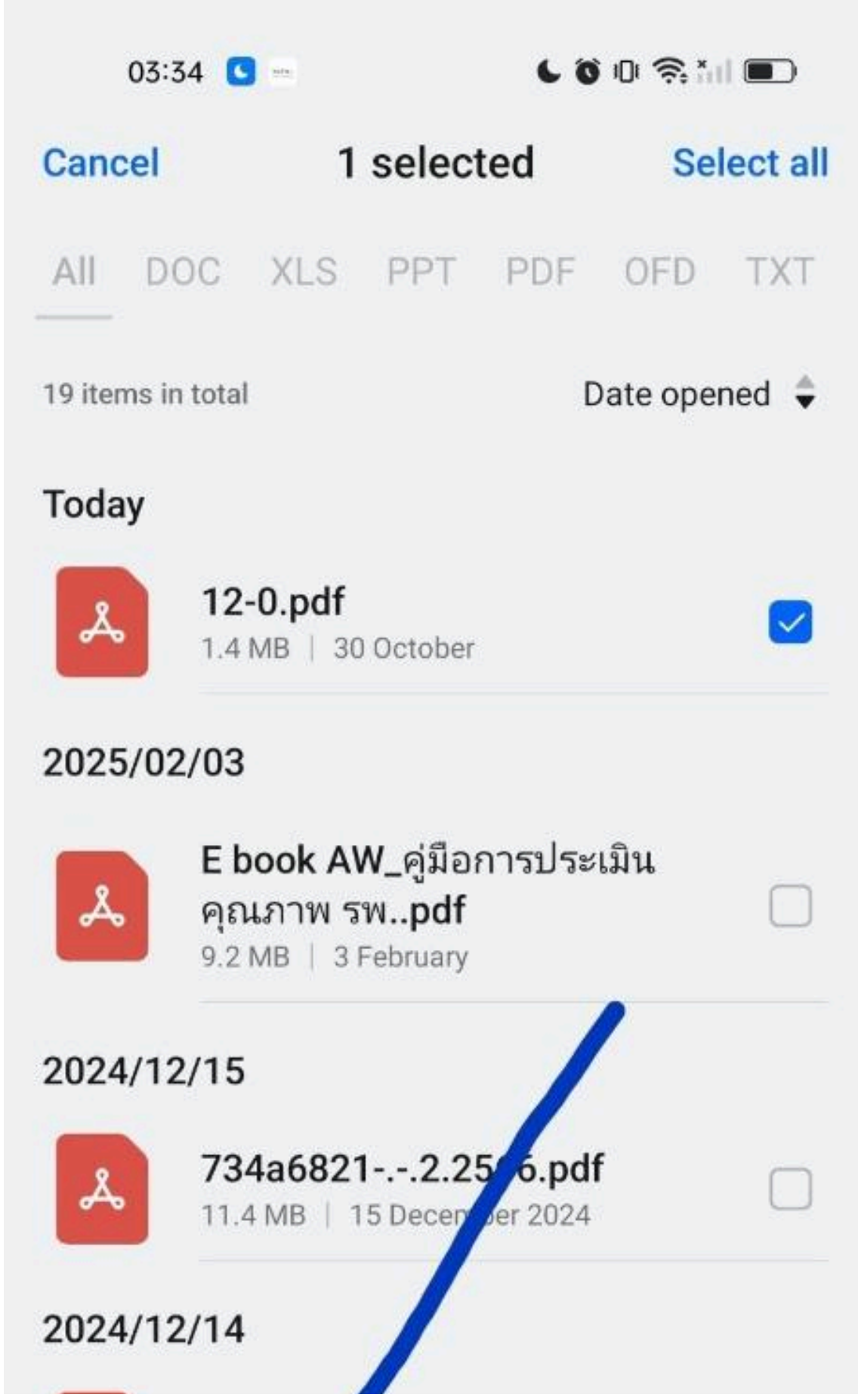
03:34
Cancel
1 selected
Select all
All DOC XLS PPT PDF OFD TXT
19 items in total
Date opened
Today
12-0.pdf
1.4 MB | 30 October
2025/02/03
E book AW_คู่มือการประเมินคุณภาพ รพ..pdf
9.2 MB | 3 February
2024/12/15
11.4 MB
2024/12/14

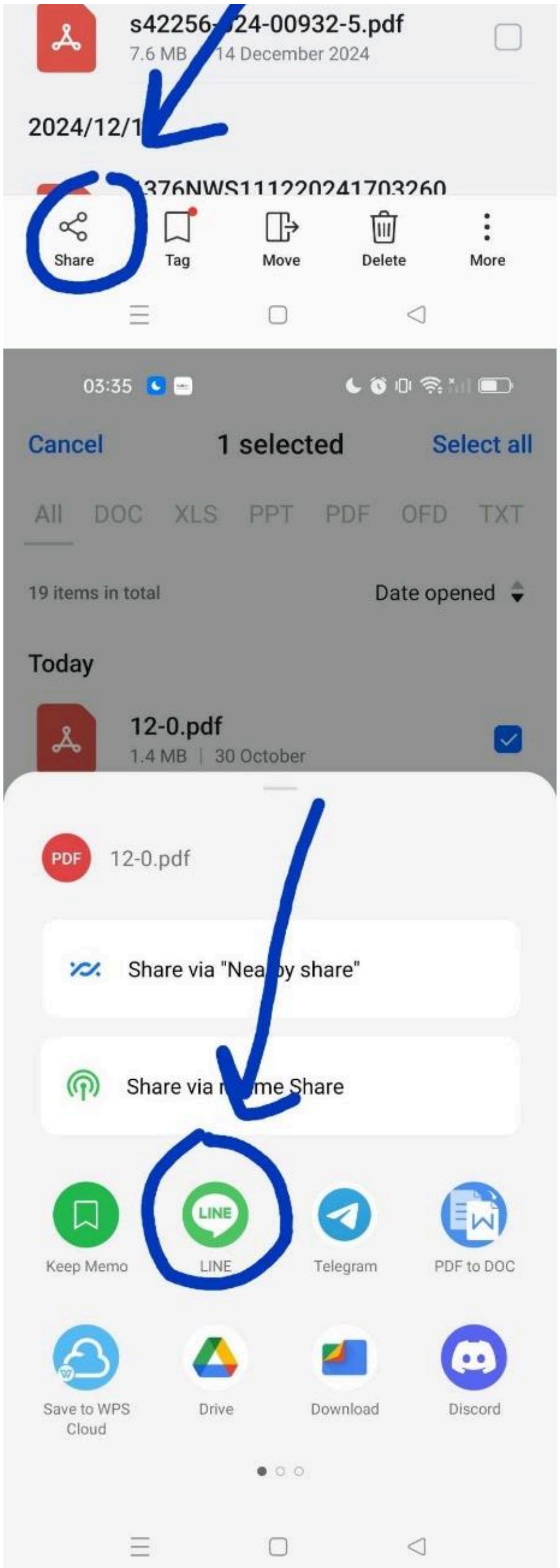

Submit

Powered by REDCap
