# Supplementary material for: Real-world effectiveness of maternal genetically inactivated acellular pertussis vaccination in protecting infants under three months of age: a case-control study using concurrent community-based and test-negative controls
Source: Infection. 2026 Jul 27;54(5):2469–83. doi: 10.1007/s15010-026-02868-x (PMC13727804; doi:10.1007/s15010-026-02868-x)
Supplement: Supplementary file 2 [file 15010_2026_2868_MOESM1_ESM.docx]

**Online Resources**

**Online Resource 1. Distribution of estimated propensity scores and inverse probability of treatment weights for cases versus community-matched controls.**

**Online Resource 2. Distribution of estimated propensity scores and inverse probability of treatment weights for cases versus test-negative controls.**

**Online Resource 3. E-value curves for the minimum association required to explain away vaccine effectiveness against symptomatic pertussis**

**Online Resource 4. E-value curves for the minimum association required to explain away vaccine effectiveness against laboratory-confirmed pertussis**

 **Online Resource 1. Distribution of estimated propensity scores and inverse probability of treatment weights for cases versus community-matched controls**

 **Online Resource 2. Distribution of estimated propensity scores and inverse probability of treatment weights for cases versus test-negative controls**

**Online Resource 3. E-value curves for the minimum association required to explain away vaccine effectiveness against symptomatic pertussis**
OR, odds ratio; CI, confidence interval; Q, quarter of the year.

**Online Resource 4. E-value curves for the minimum association required to explain away vaccine effectiveness against laboratory-confirmed pertussis**
OR, odds ratio; CI, confidence interval; Q, quarter of the year.
